# Supplementary material for: Physical activity interventions for the cardiac population: A generic logic model based on intervention mapping
Source: PLoS One. 2025 May 14;20(5):e0322807. doi: 10.1371/journal.pone.0322807 (PMC12077670; doi:10.1371/journal.pone.0322807)
Supplement: S2 file — (PPTX) [file pone.0322807.s002.pptx]

## Slide 1
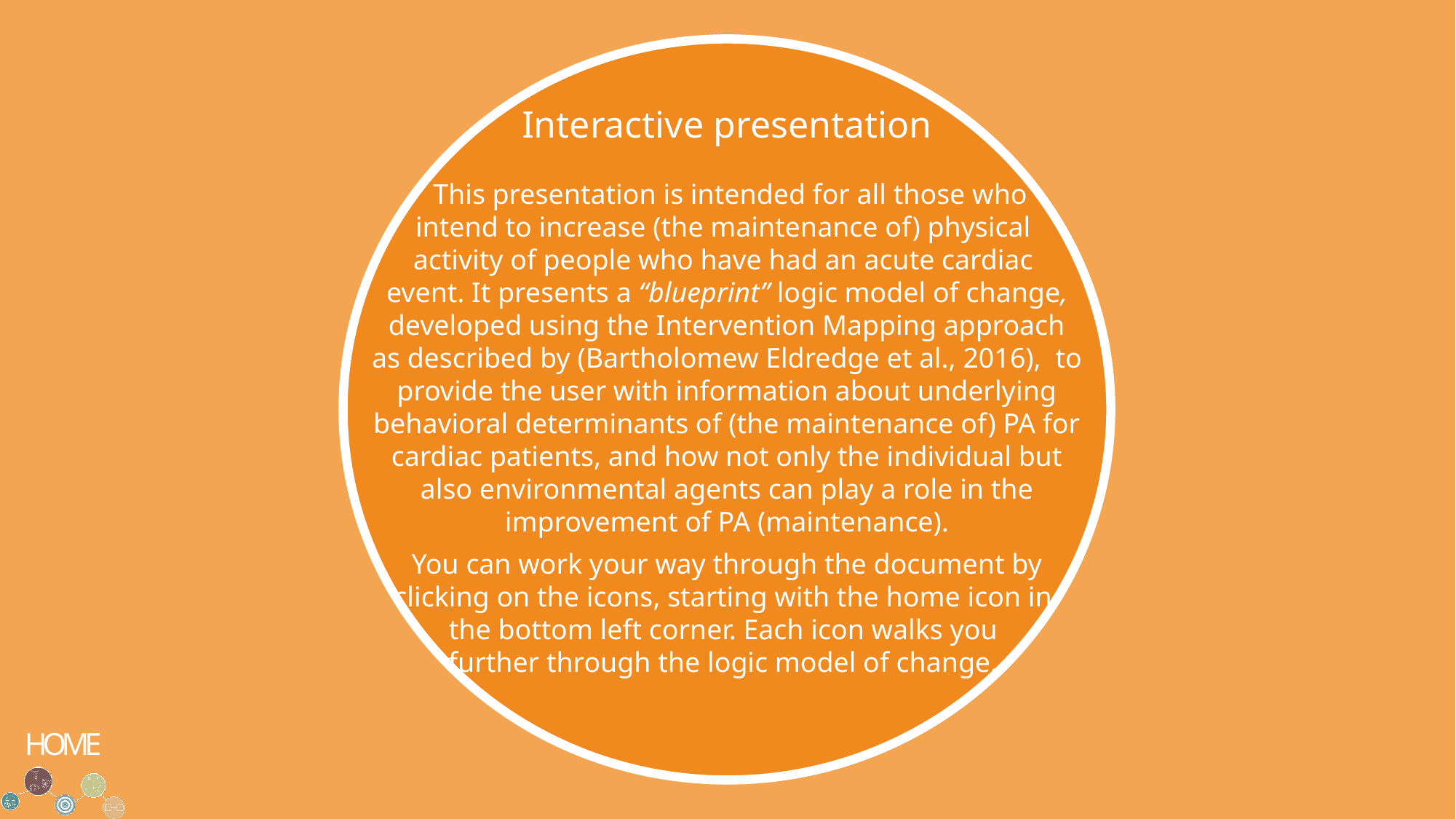

Explanation
Interactive presentation
 This presentation is intended for all those who
intend to increase (the maintenance of) physical
activity of people who have had an acute cardiac
event. It presents a “blueprint” logic model of change, developed using the Intervention Mapping approach as described by (Bartholomew Eldredge et al., 2016), to provide the user with information about underlying behavioral determinants of (the maintenance of) PA for cardiac patients, and how not only the individual but also environmental agents can play a role in the improvement of PA (maintenance).
You can work your way through the document by clicking on the icons, starting with the home icon in
the bottom left corner. Each icon walks you
further through the logic model of change.
HOME

## Slide 2
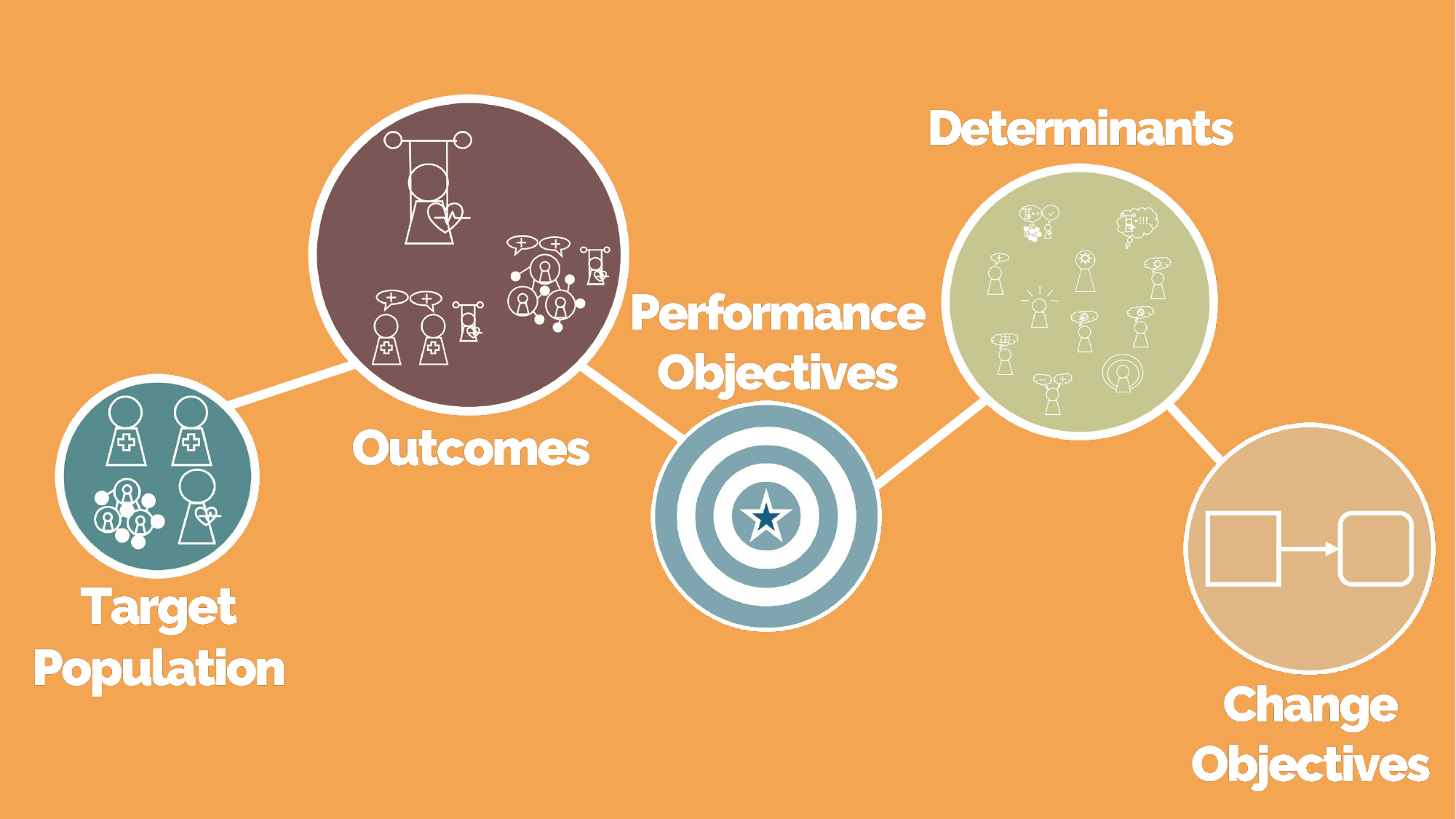

Homepage

## Slide 3
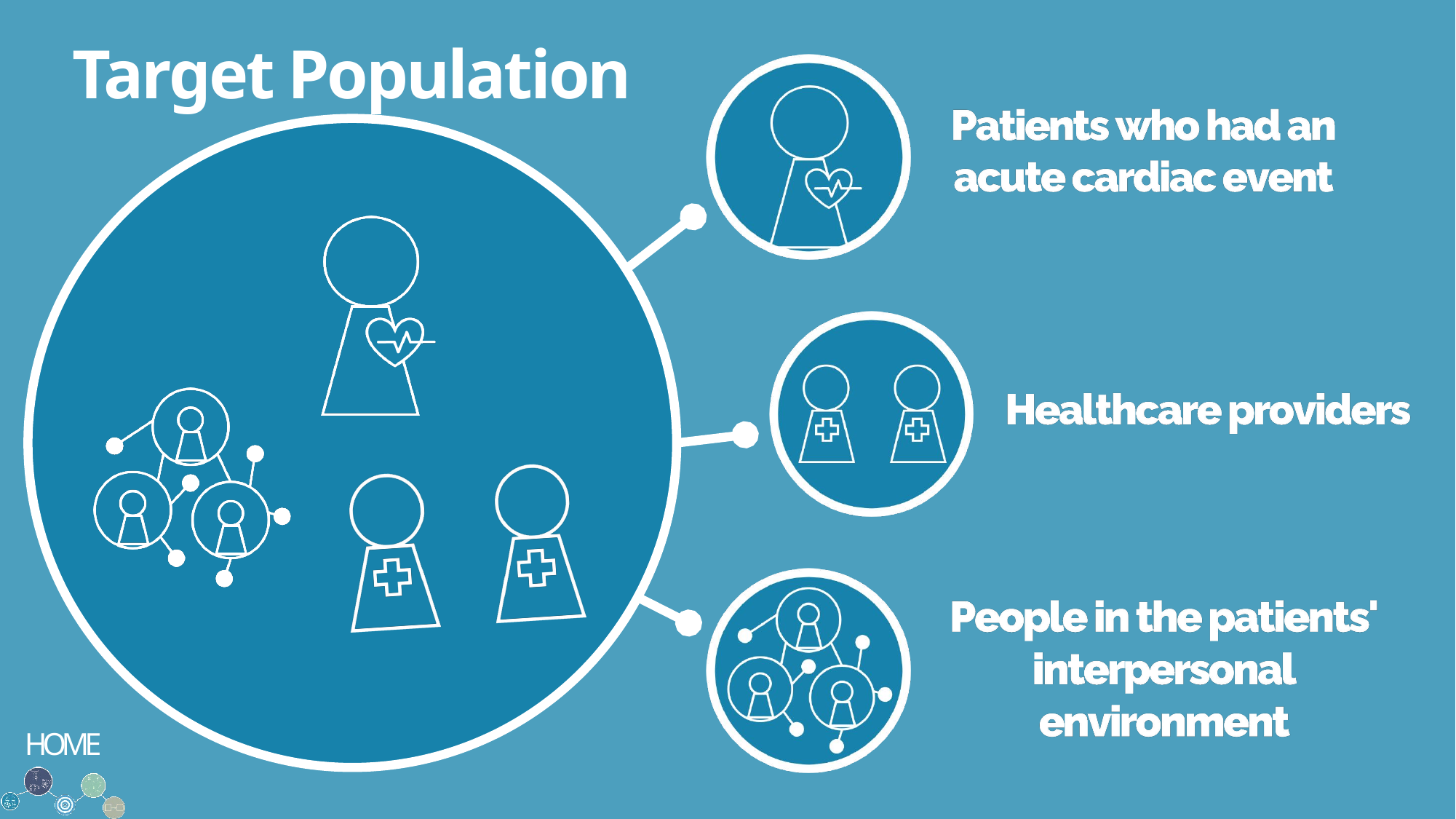

Target Population
Target Population
HOME

## Slide 4
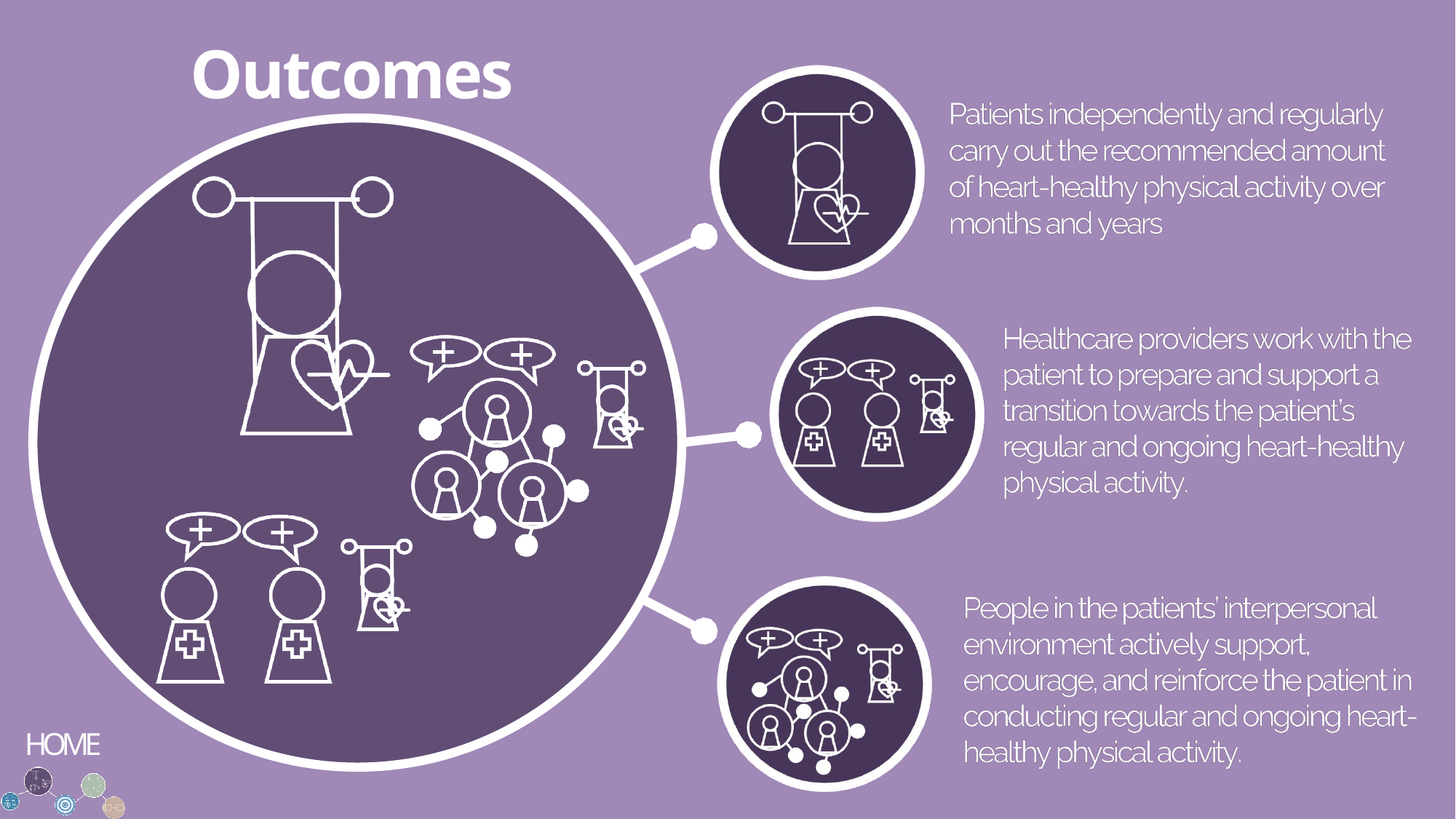

Behavioural and Environmental Outcomes
Outcomes
HOME

## Slide 5
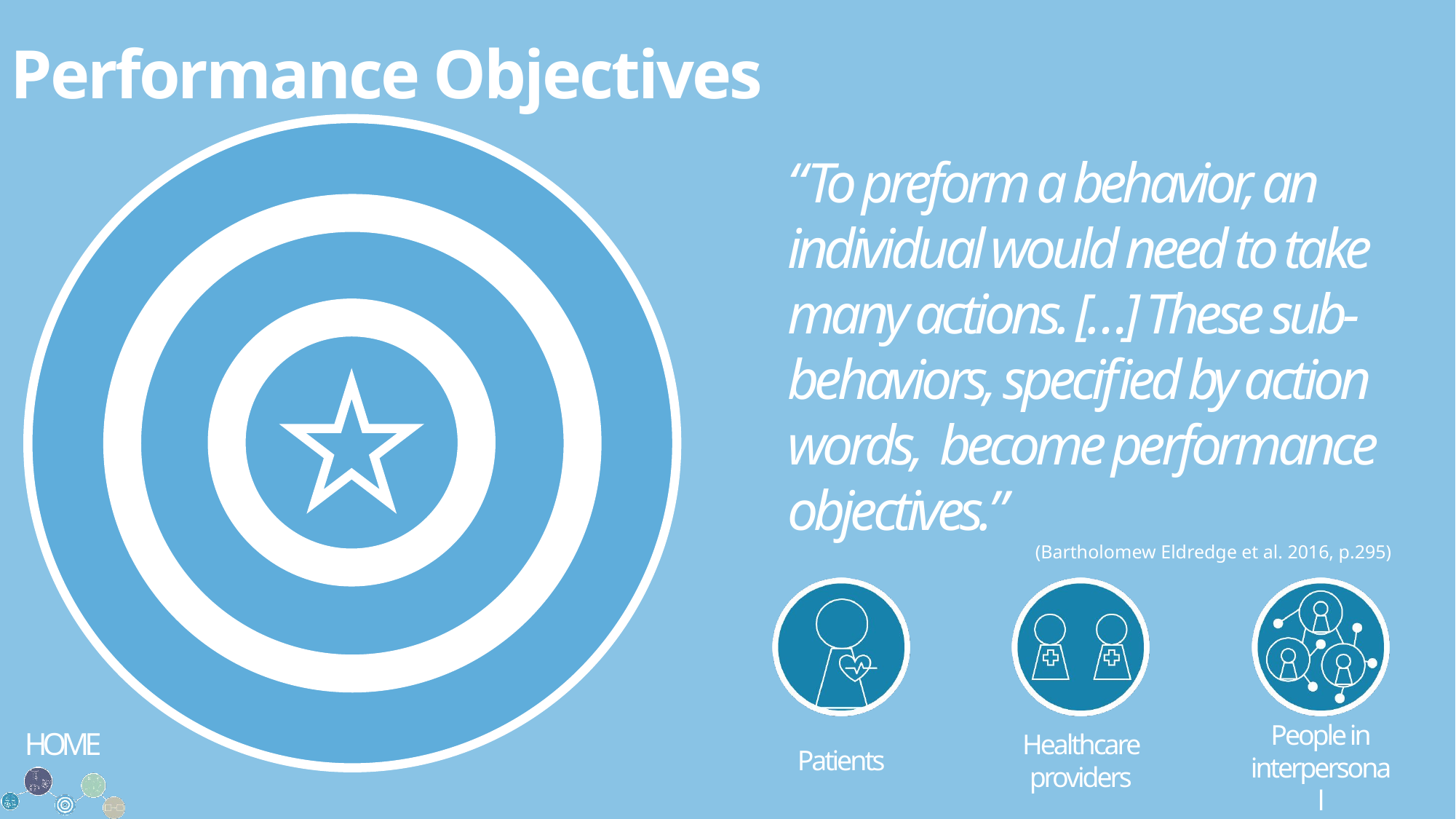

Performance Objectives
Performance Objectives
“To preform a behavior, an individual would need to take many actions. […] These sub-behaviors, specified by action words, become performance objectives.”
(Bartholomew Eldredge et al. 2016, p.295)
People in interpersonal environment
HOME
Healthcare providers
Patients

## Slide 6
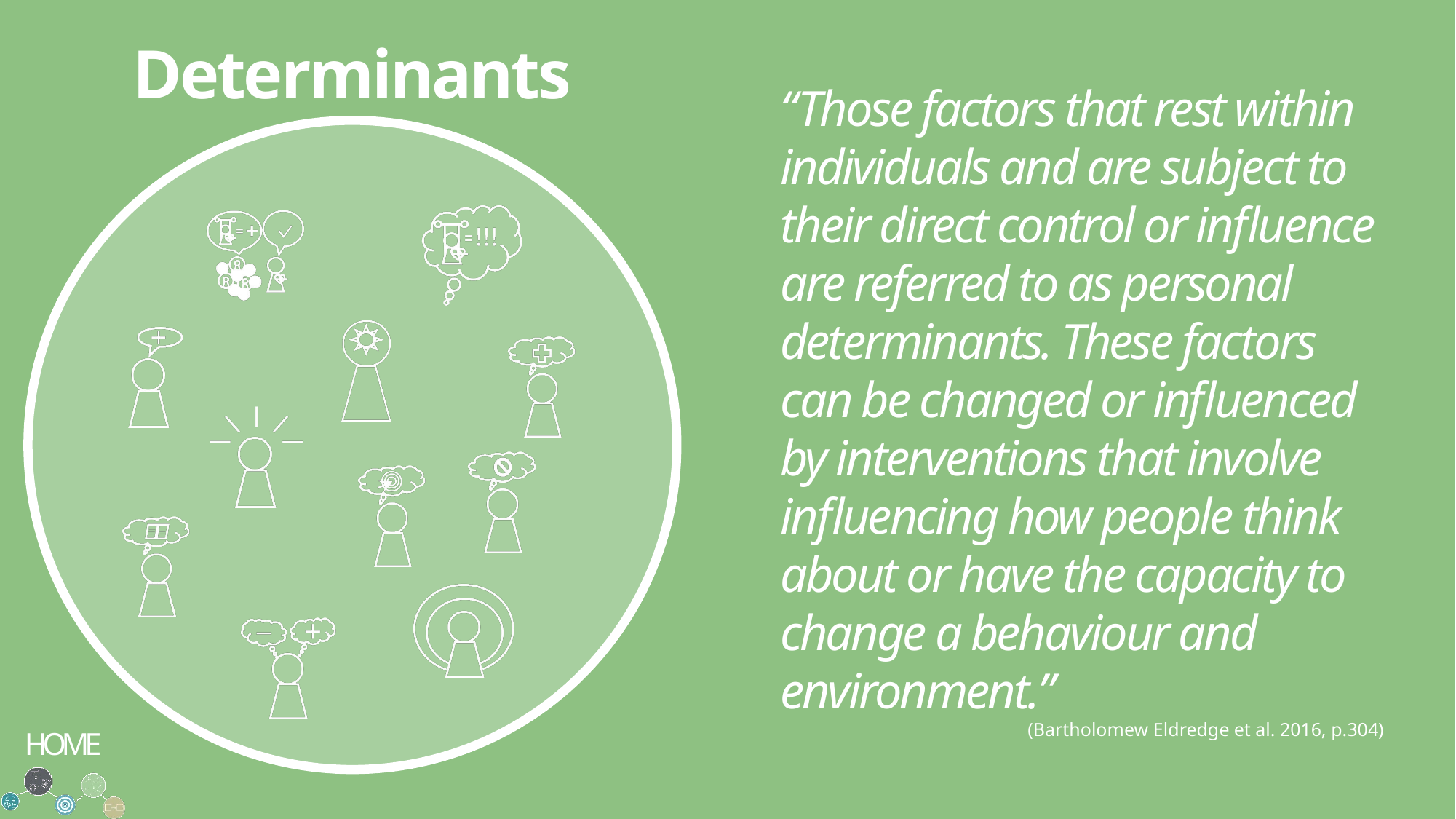

Determinants
Determinants
“Those factors that rest within individuals and are subject to their direct control or influence are referred to as personal determinants. These factors can be changed or influenced by interventions that involve influencing how people think about or have the capacity to change a behaviour and environment.”
(Bartholomew Eldredge et al. 2016, p.304)
HOME

## Slide 7
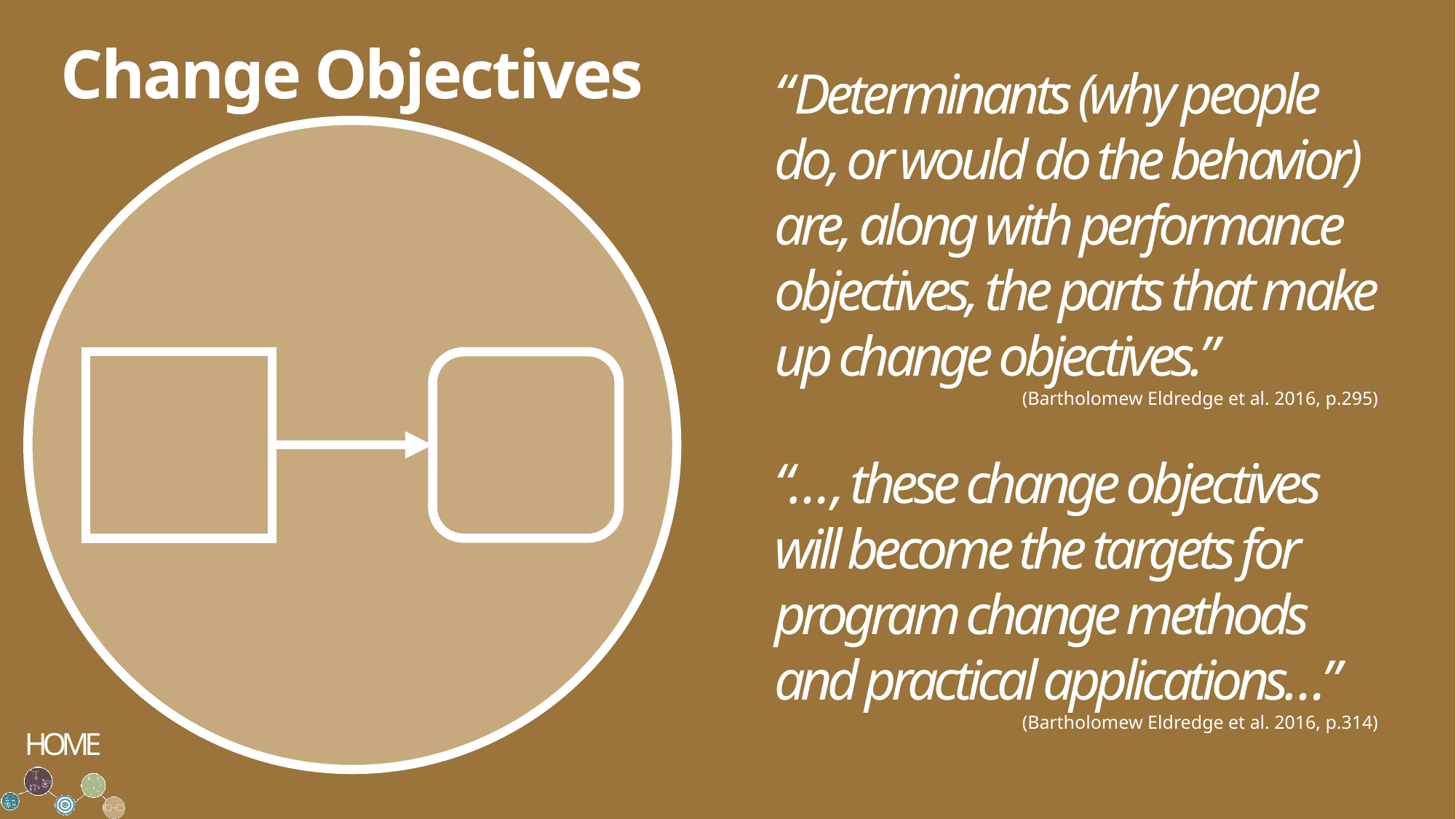

Change Objectives
Change Objectives
“Determinants (why people do, or would do the behavior) are, along with performance objectives, the parts that make up change objectives.”
(Bartholomew Eldredge et al. 2016, p.295)
“…, these change objectives will become the targets for program change methods and practical applications…”
(Bartholomew Eldredge et al. 2016, p.314)
HOME

## Slide 8
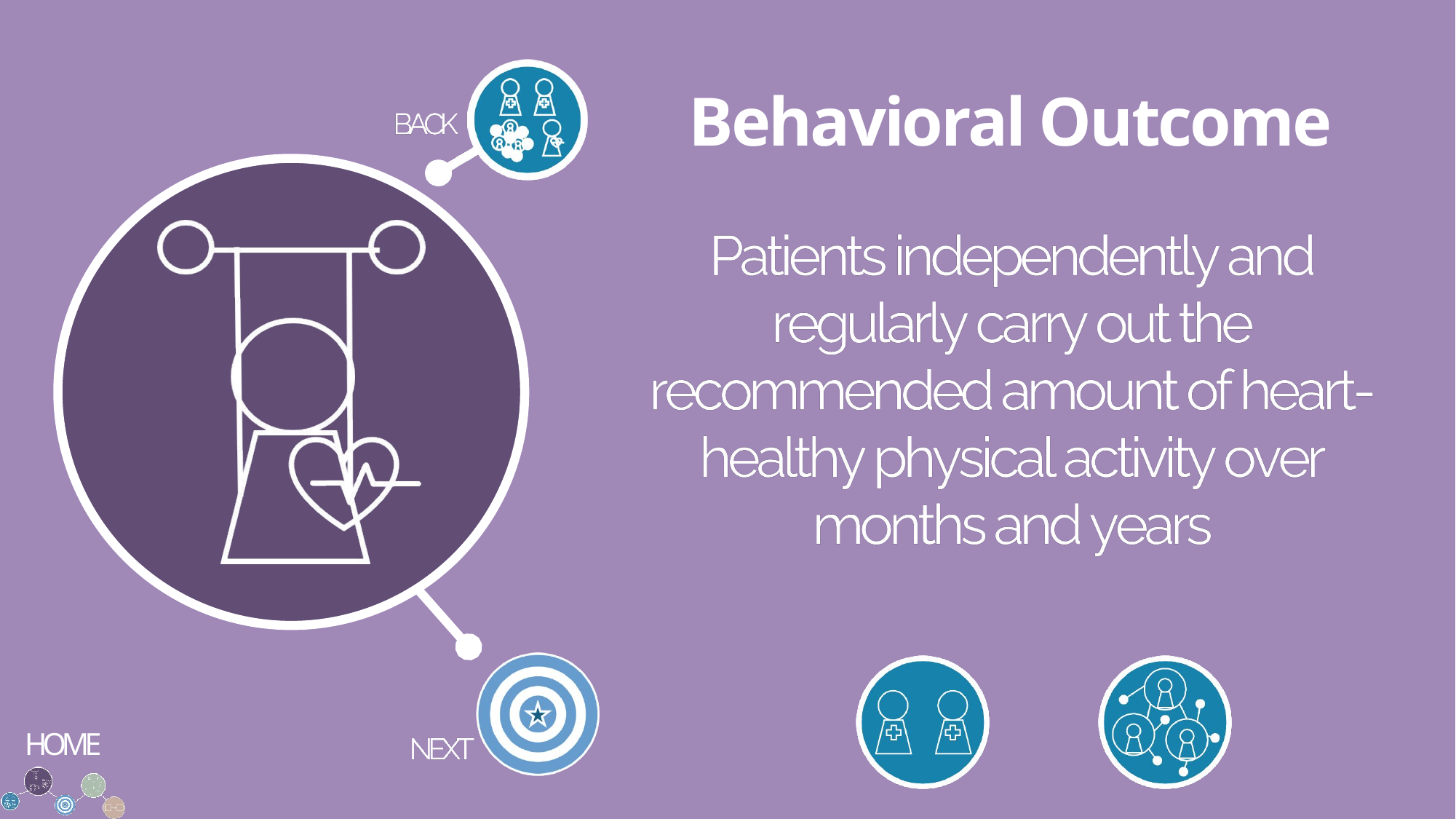

Individual behavioural outcome
Behavioral Outcome
HOME

## Slide 9
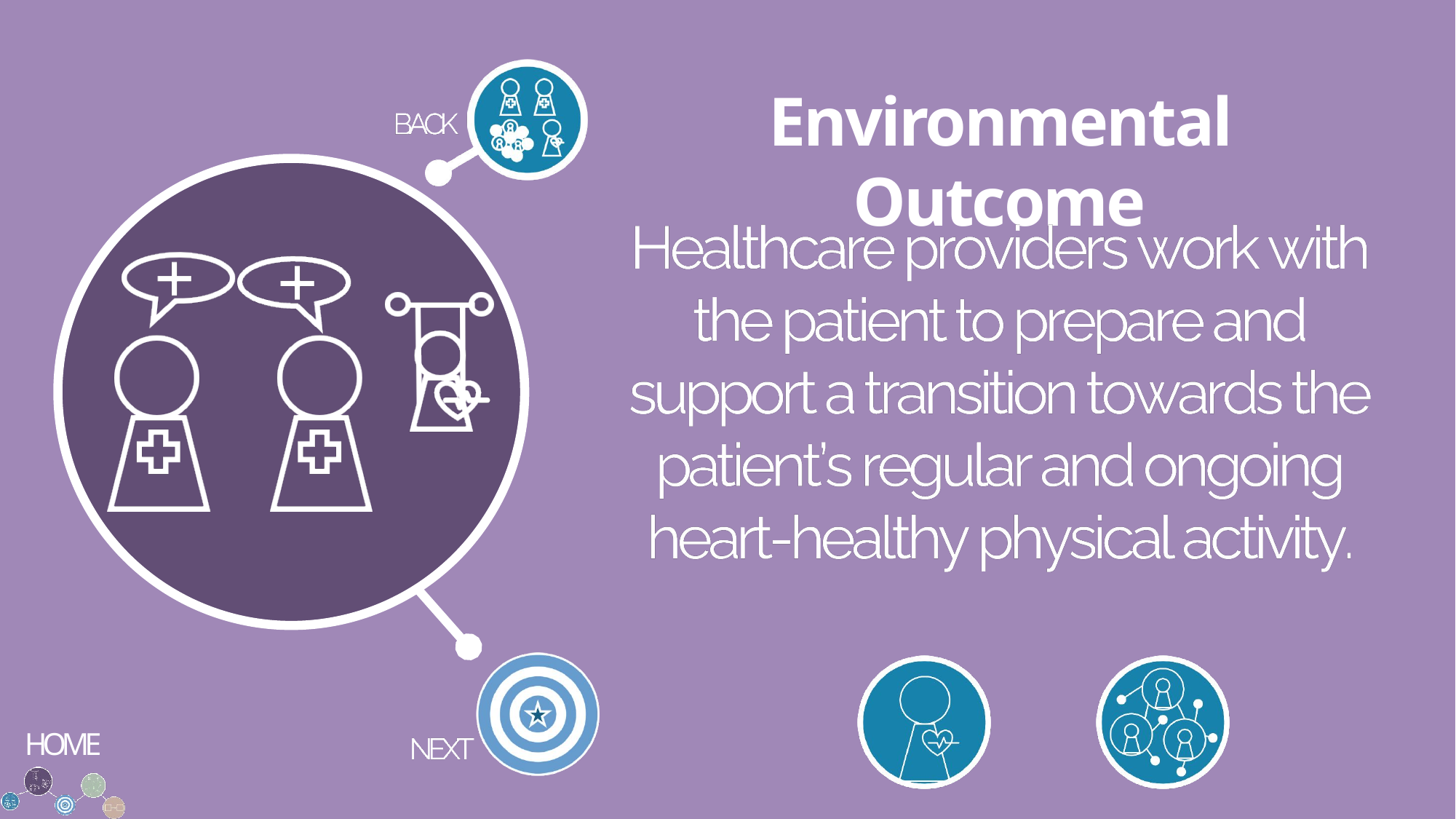

Environmental outcome for Healthcare Providers
Environmental Outcome
HOME

## Slide 10
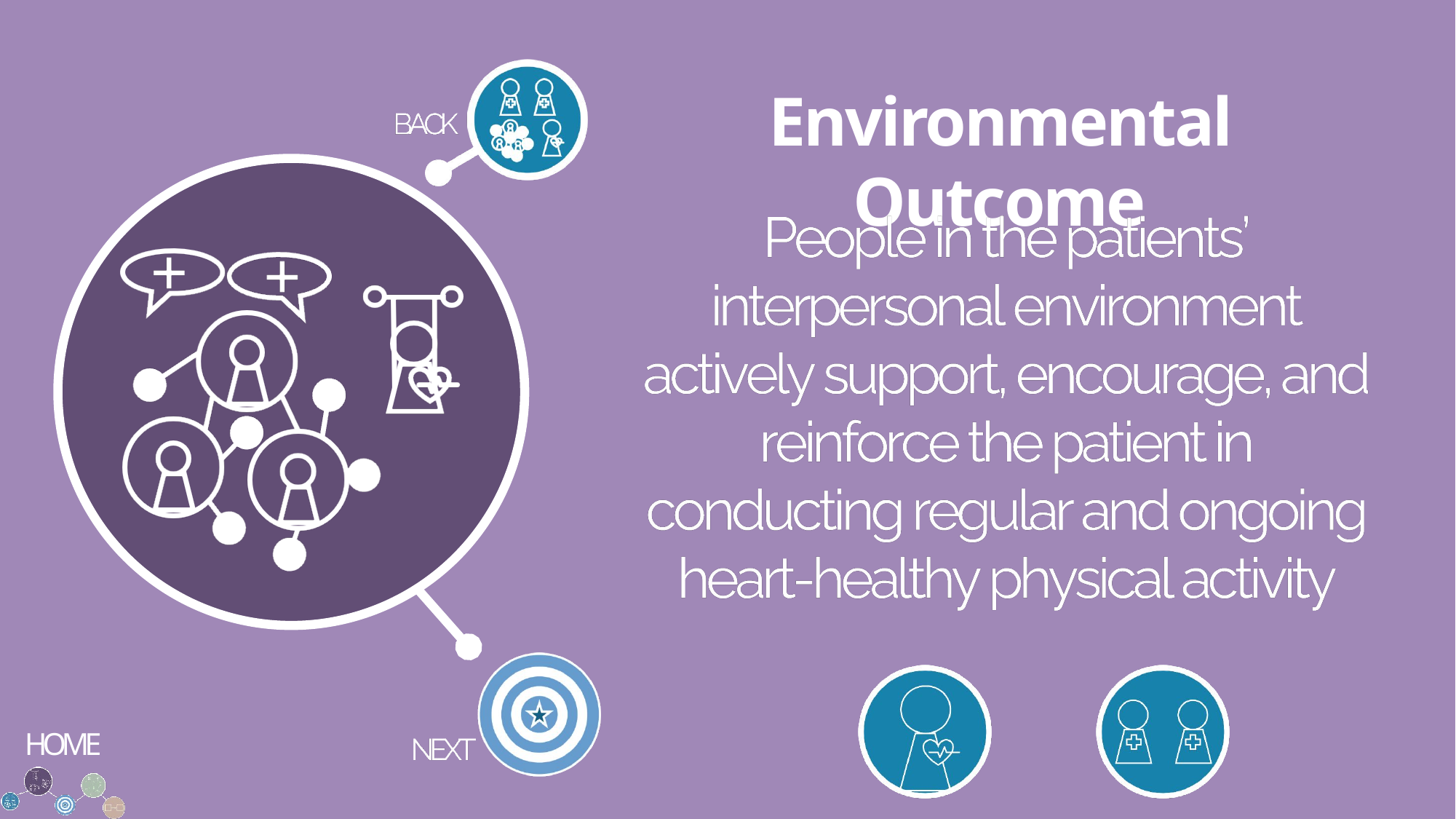

Environmental Outcome for People in the patients’ interpersonal environment
Environmental Outcome
HOME

## Slide 11
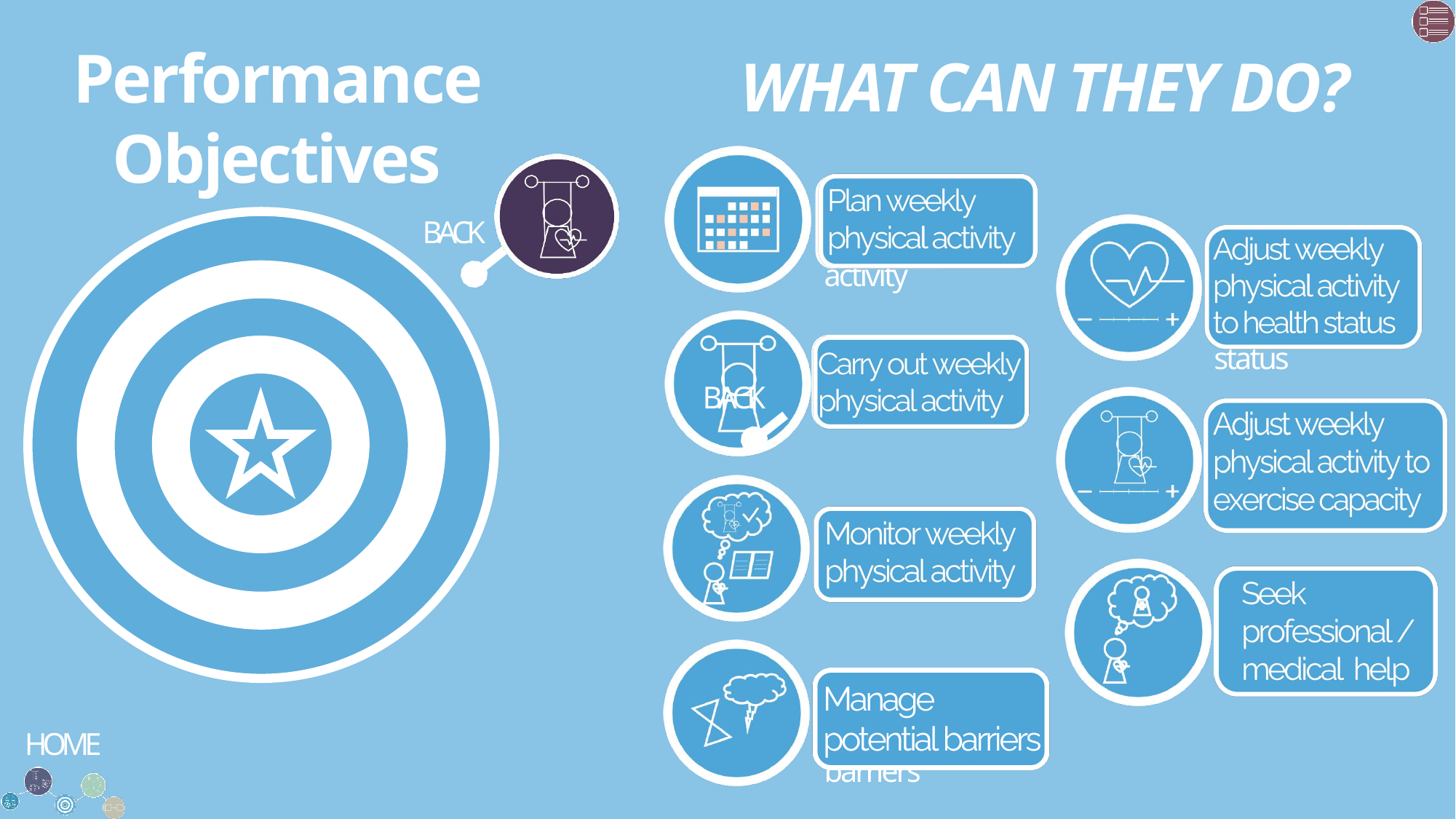

Performance Objectives for Patients
Performance Objectives
WHAT CAN THEY DO?
Plan weekly physical activity
BACK
Adjust weekly physical activity to health status
Carry out weekly physical activity
BACK
Adjust weekly physical activity to exercise capacity
Monitor weekly physical activity
Seek professional / medical help
Manage potential barriers
HOME

## Slide 12
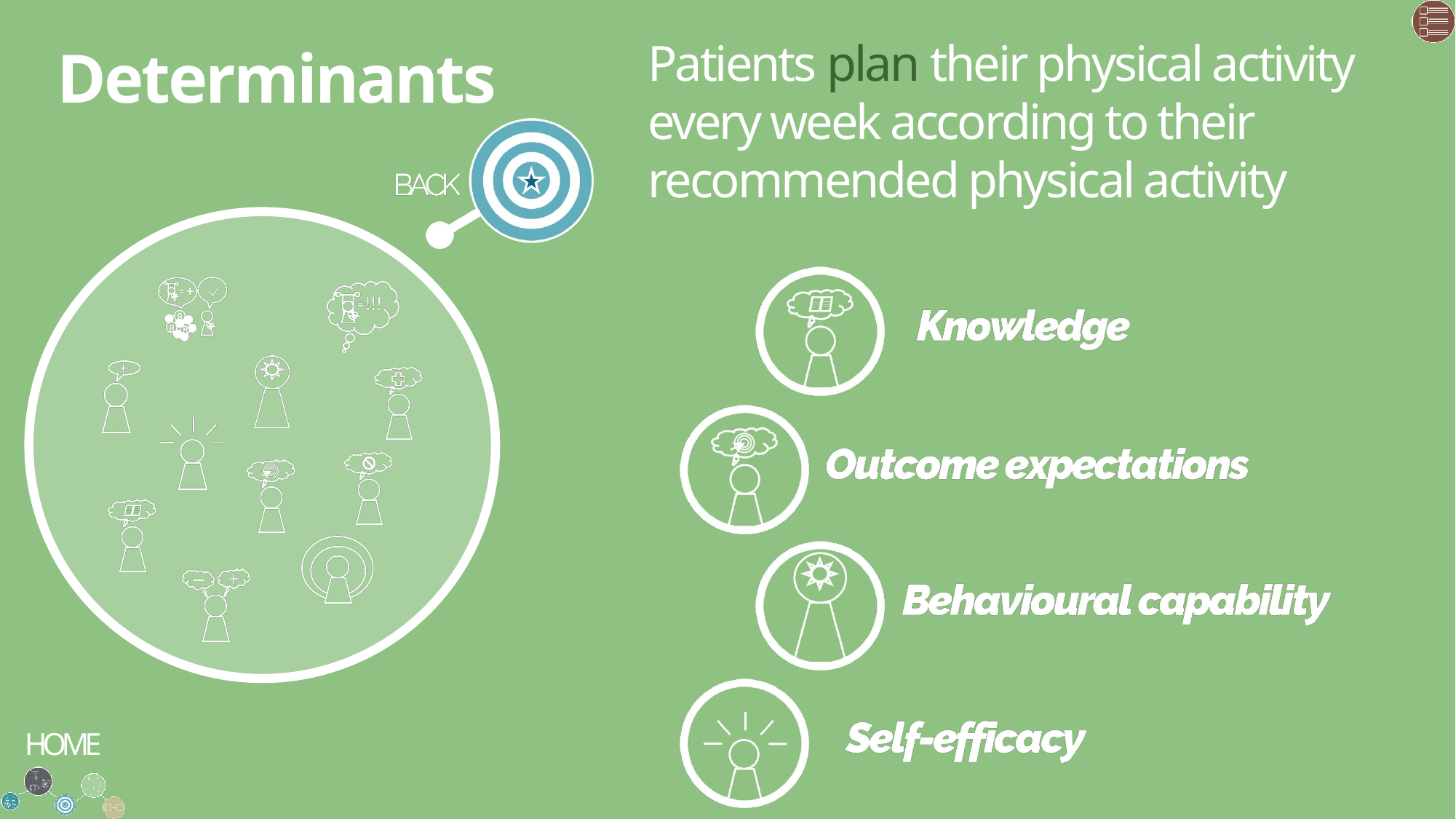

PO1 Determinants for PO1 for patients
Patients plan their physical activity every week according to their recommended physical activity
Determinants
HOME

## Slide 13
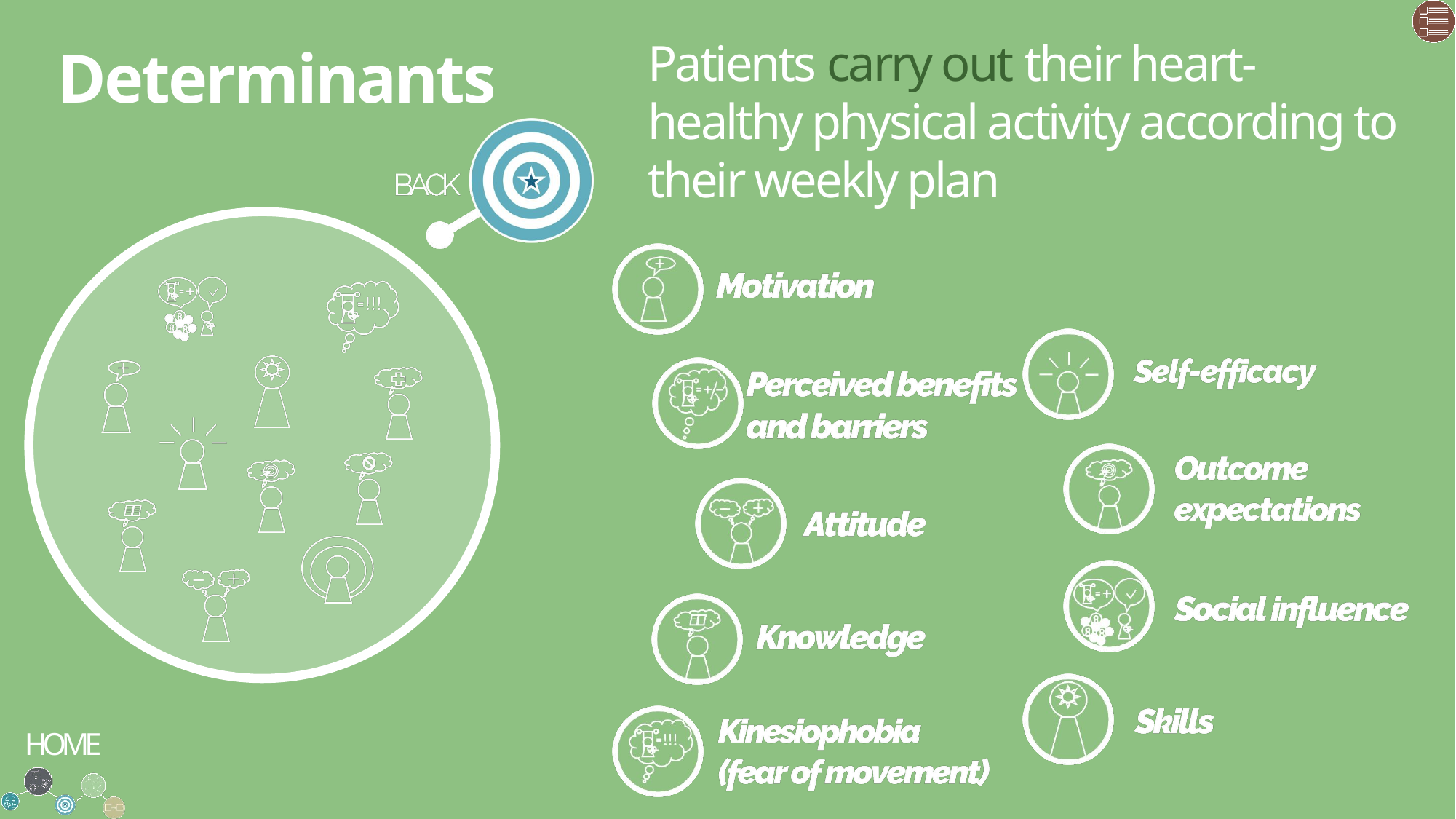

PO2 Determinants for PO2 for patients
Patients carry out their heart-healthy physical activity according to their weekly plan
Determinants
HOME

## Slide 14
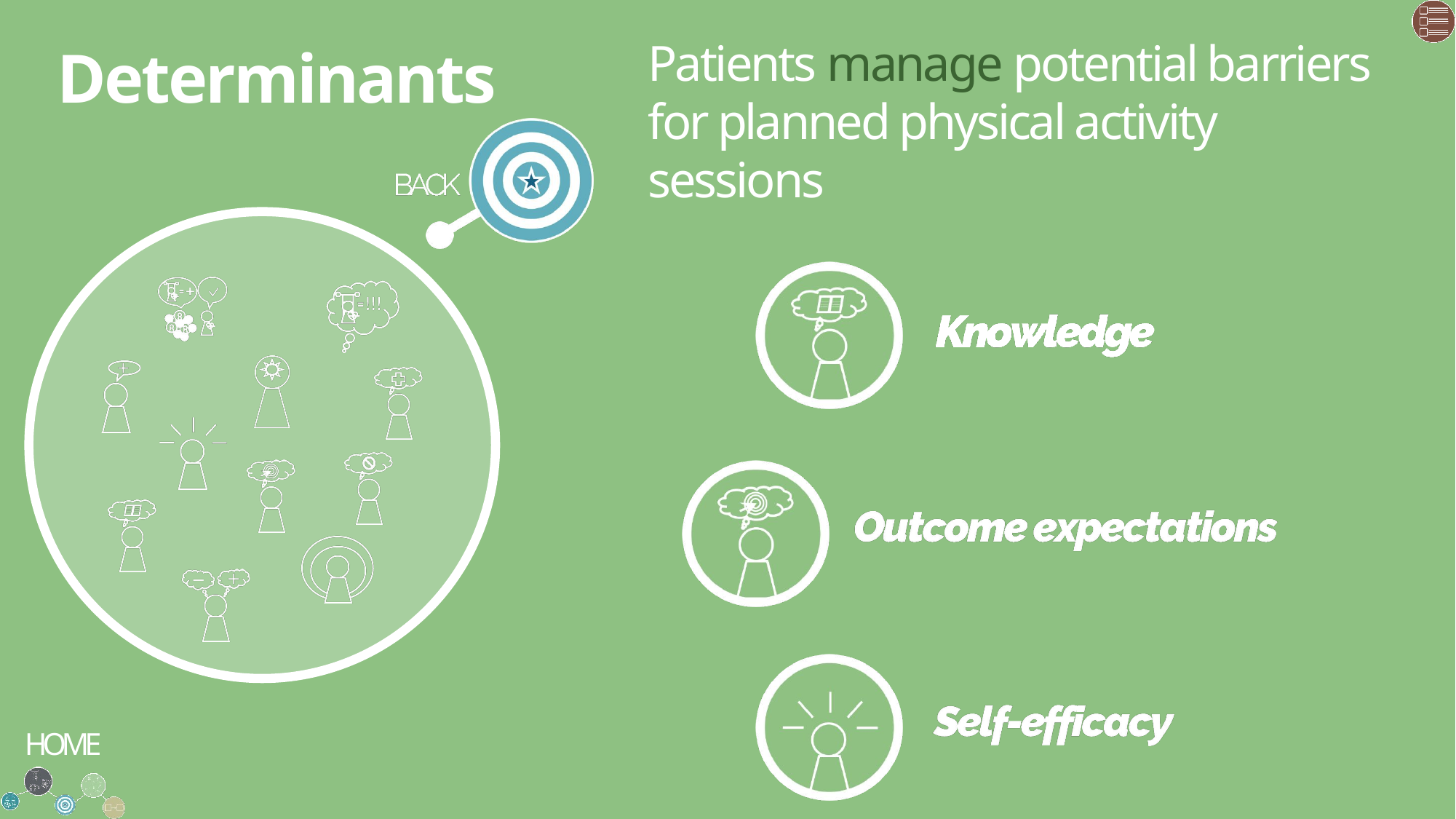

PO3 Determinants for PO3 for patients
Patients manage potential barriers for planned physical activity sessions
Determinants
HOME

## Slide 15
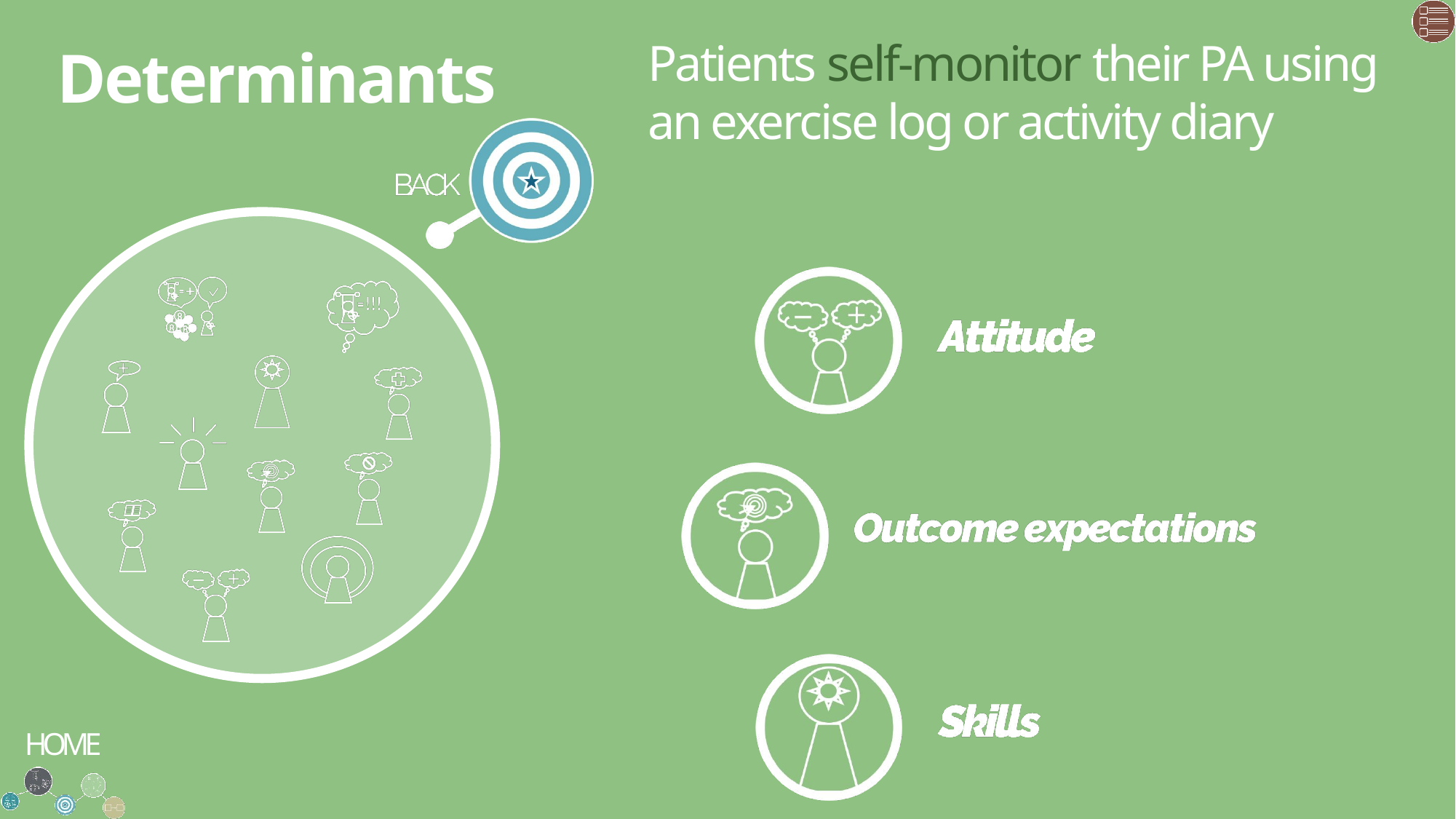

PO4 Determinants for PO4 for patients
Patients self-monitor their PA using an exercise log or activity diary
Determinants
HOME

## Slide 16
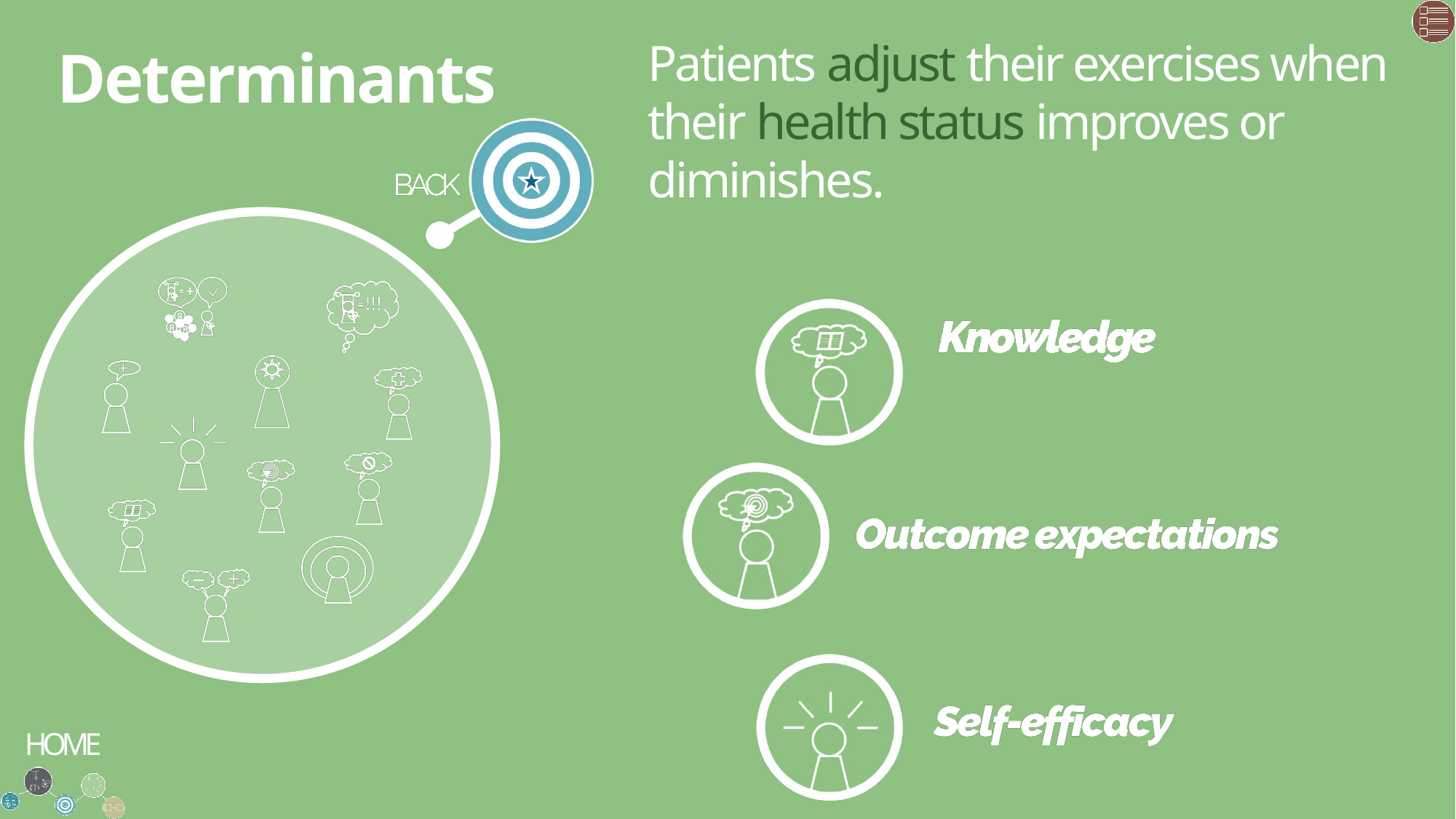

PO5 Determinants for PO5 for patients
Patients adjust their exercises when their health status improves or diminishes.
Determinants
HOME

## Slide 17
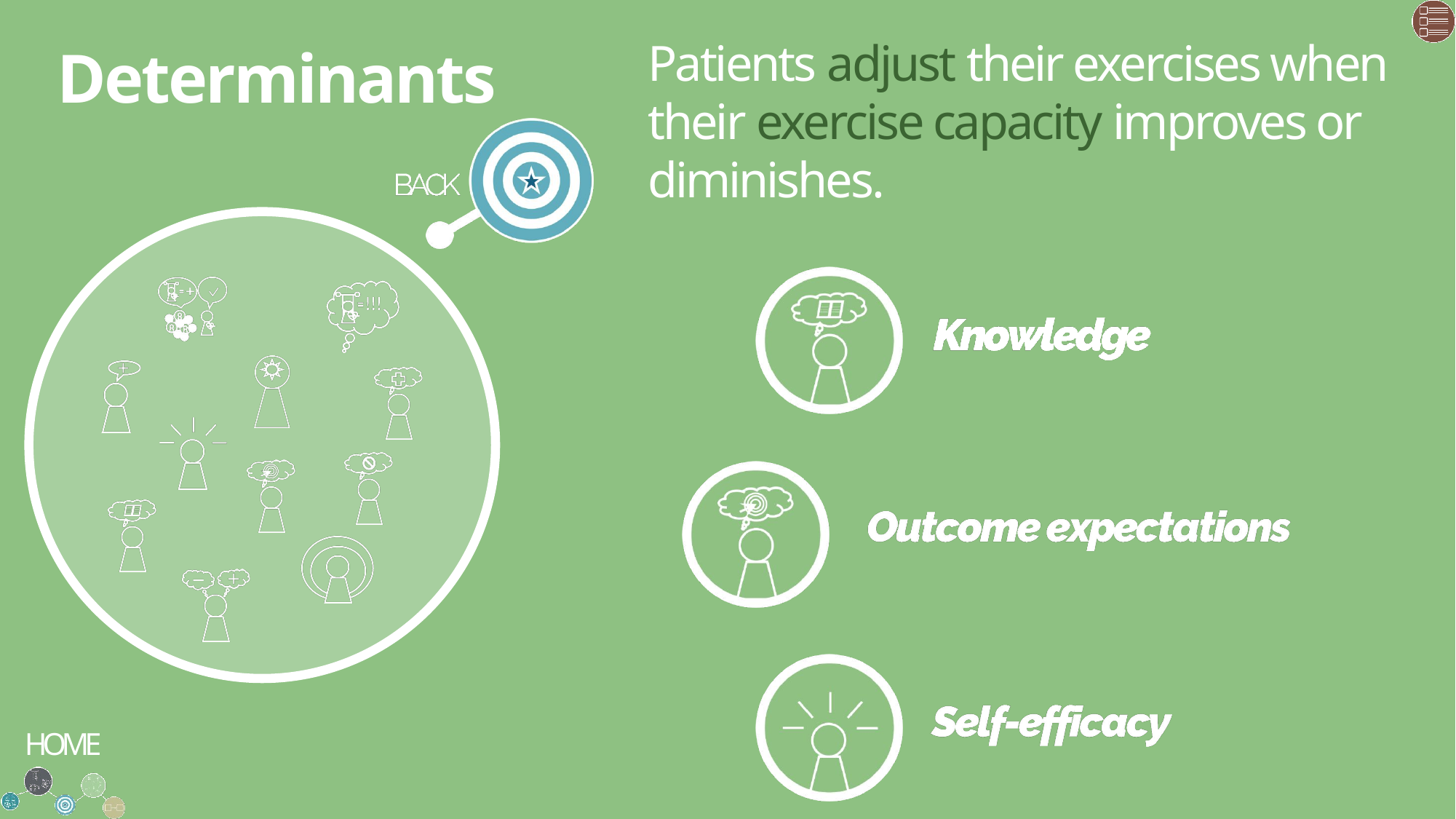

PO6 Determinants for PO6 for patients
Patients adjust their exercises when their exercise capacity improves or diminishes.
Determinants
HOME

## Slide 18
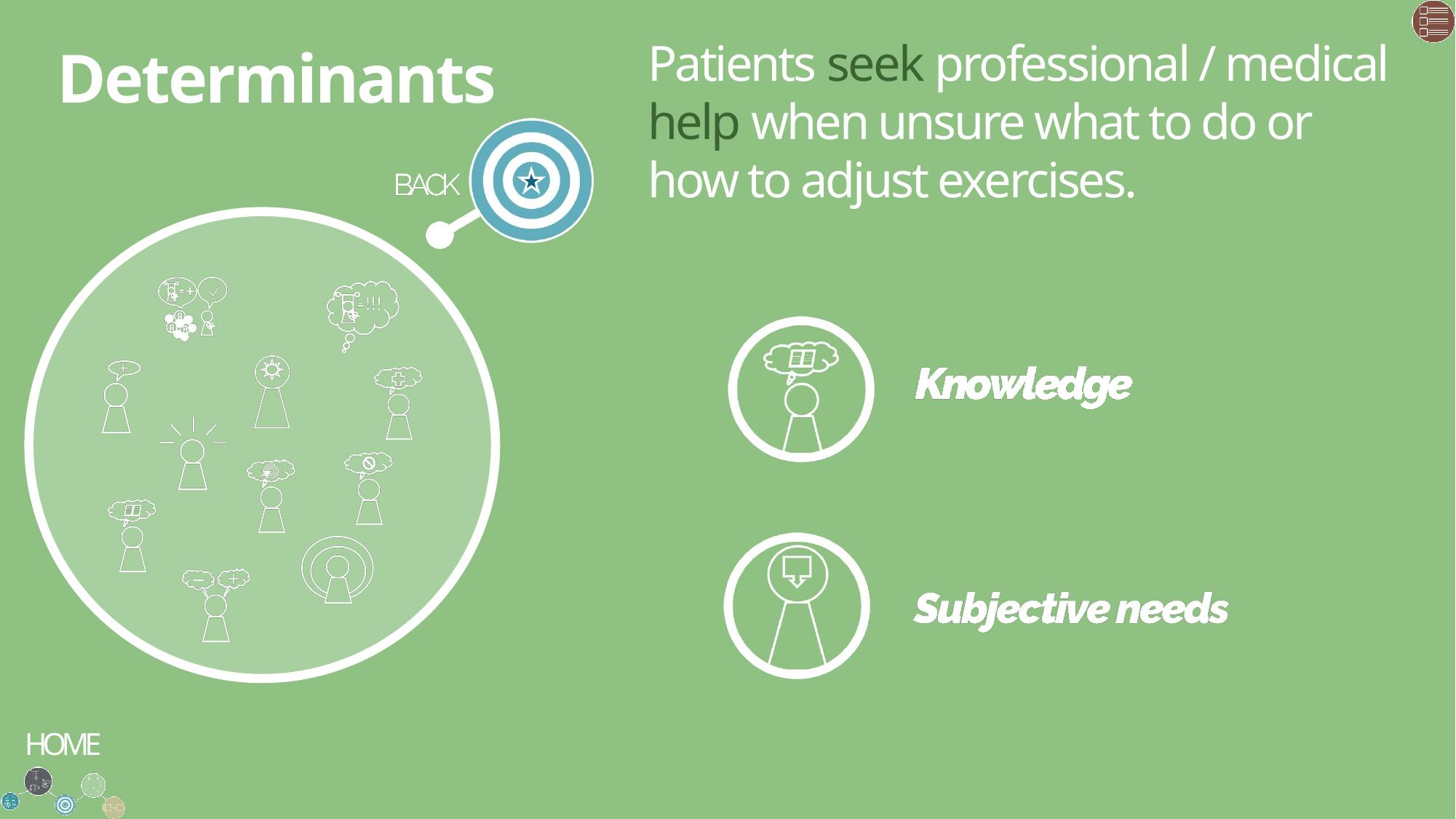

PO7 Determinants for PO7 for patients
Patients seek professional / medical help when unsure what to do or how to adjust exercises.
Determinants
HOME

## Slide 19
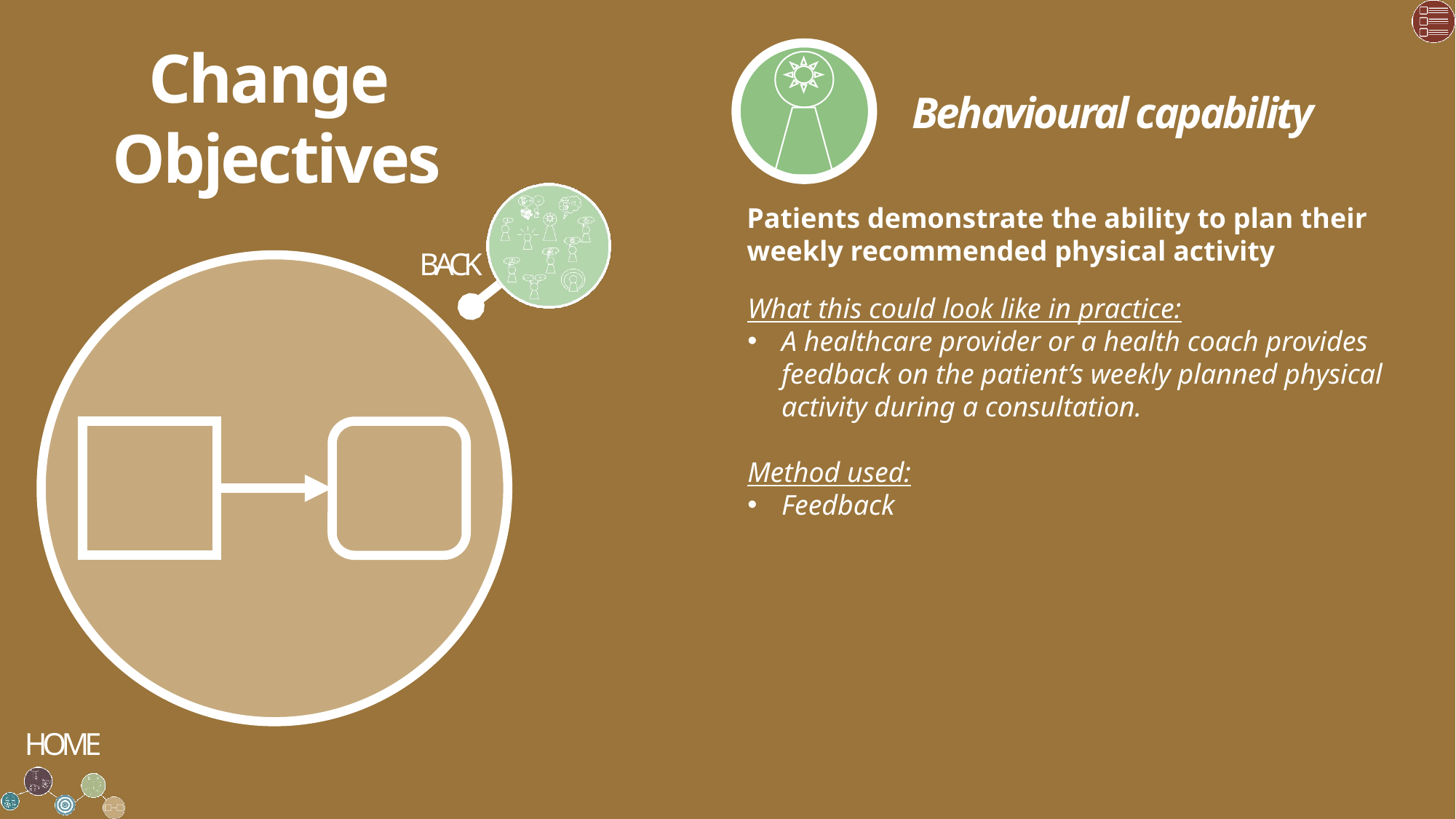

PO1 BC Change Objectives for PO1 for Patients
Change
Objectives
Behavioural capability
Patients demonstrate the ability to plan their weekly recommended physical activity
BACK
What this could look like in practice:
A healthcare provider or a health coach provides feedback on the patient’s weekly planned physical activity during a consultation.
Method used:
Feedback
HOME

## Slide 20
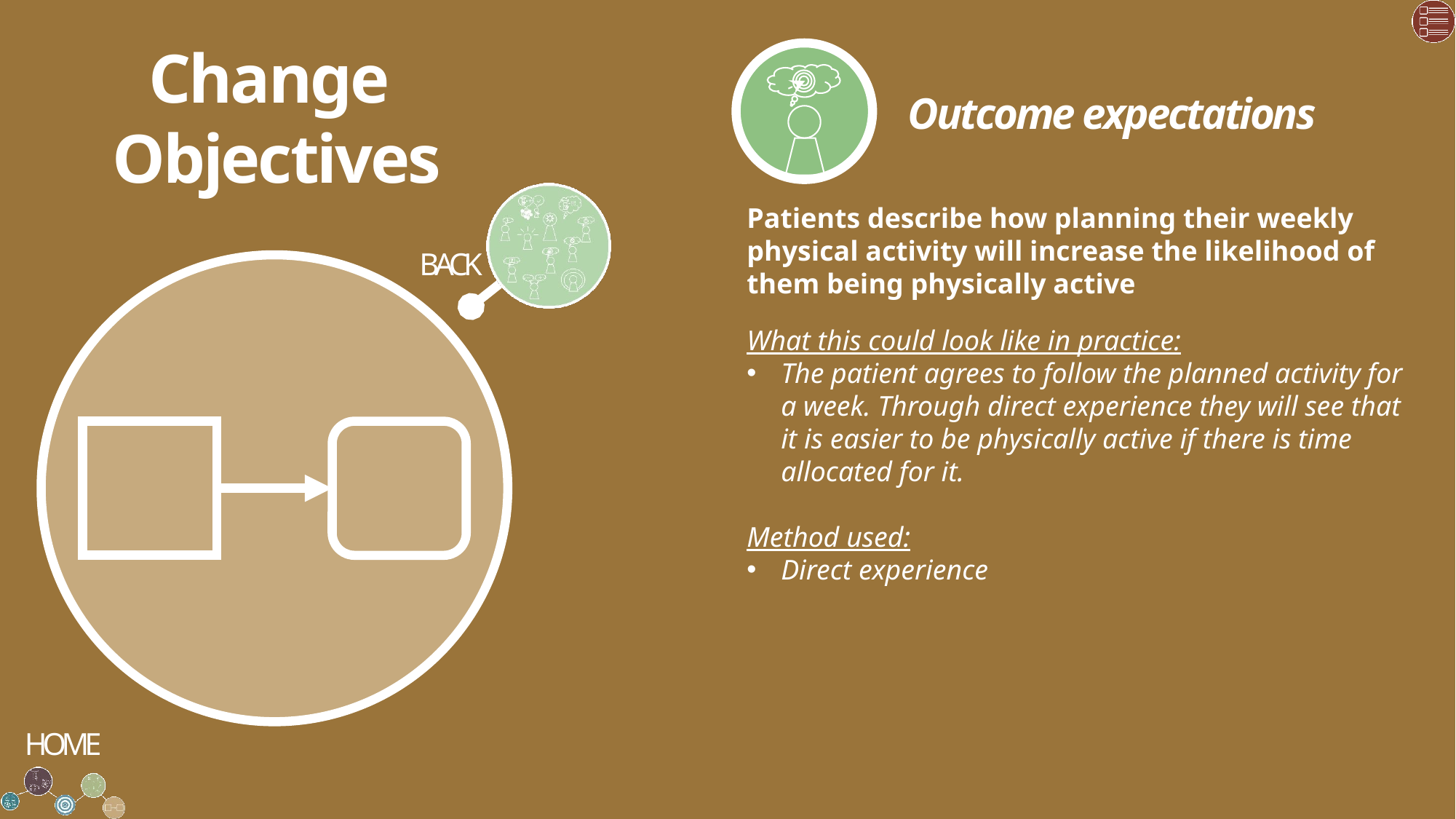

PO1 OE Change Objectives for PO1 for Patients
Change
Objectives
Outcome expectations
Patients describe how planning their weekly physical activity will increase the likelihood of them being physically active
BACK
What this could look like in practice:
The patient agrees to follow the planned activity for a week. Through direct experience they will see that it is easier to be physically active if there is time allocated for it.
Method used:
Direct experience
HOME

## Slide 21
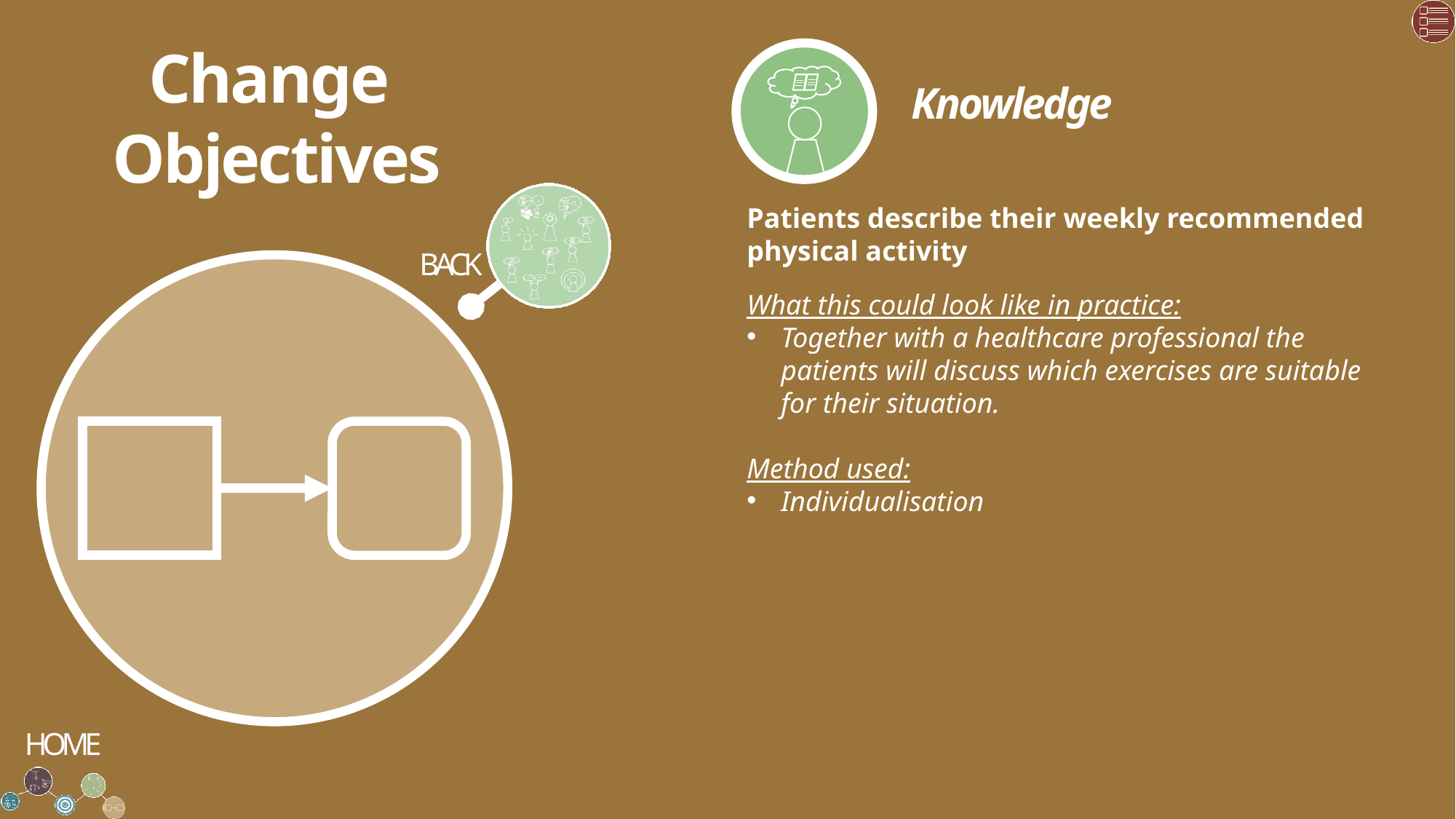

PO1 K Change Objectives for PO1 for Patients
Change
Objectives
Knowledge
Patients describe their weekly recommended physical activity
BACK
What this could look like in practice:
Together with a healthcare professional the patients will discuss which exercises are suitable for their situation.
Method used:
Individualisation
HOME

## Slide 22
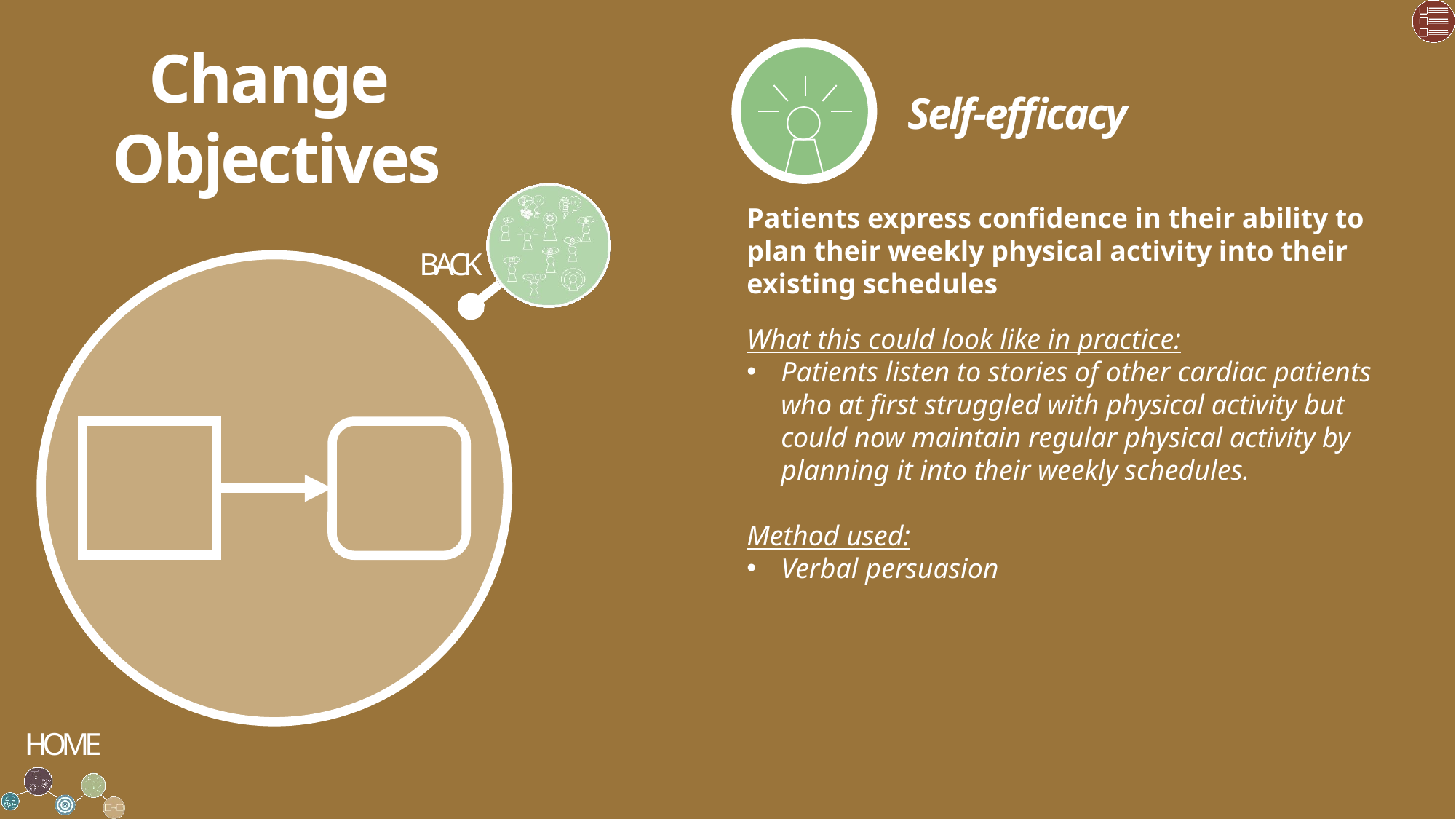

PO1 SE Change Objectives for PO2 for Patients
Change
Objectives
Self-efficacy
Patients express confidence in their ability to plan their weekly physical activity into their existing schedules
BACK
What this could look like in practice:
Patients listen to stories of other cardiac patients who at first struggled with physical activity but could now maintain regular physical activity by planning it into their weekly schedules.
Method used:
Verbal persuasion
HOME

## Slide 23
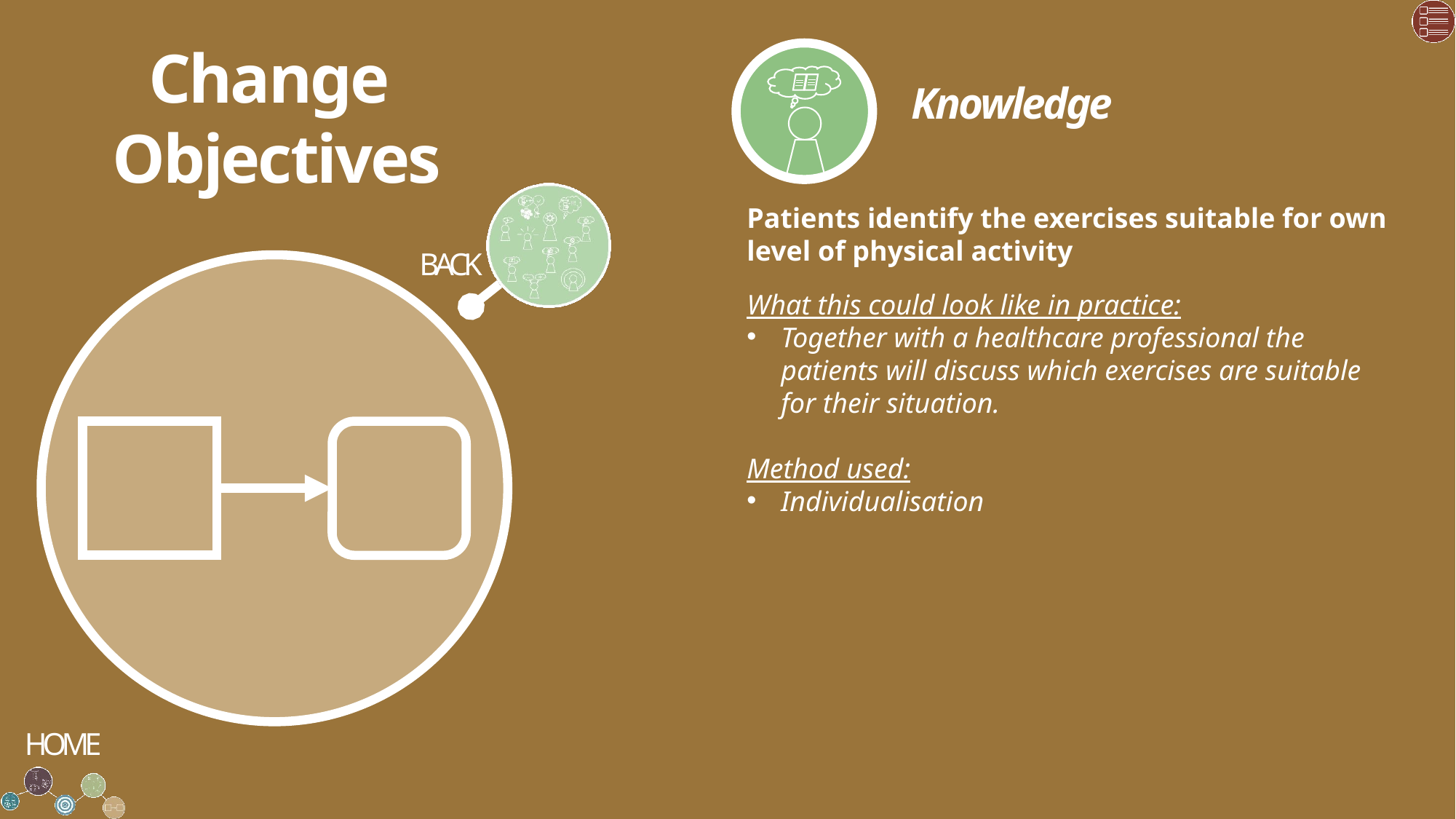

PO2 K Change Objectives for PO2 for Patients
Change
Objectives
Knowledge
Patients identify the exercises suitable for own level of physical activity
BACK
What this could look like in practice:
Together with a healthcare professional the patients will discuss which exercises are suitable for their situation.
Method used:
Individualisation
HOME

## Slide 24
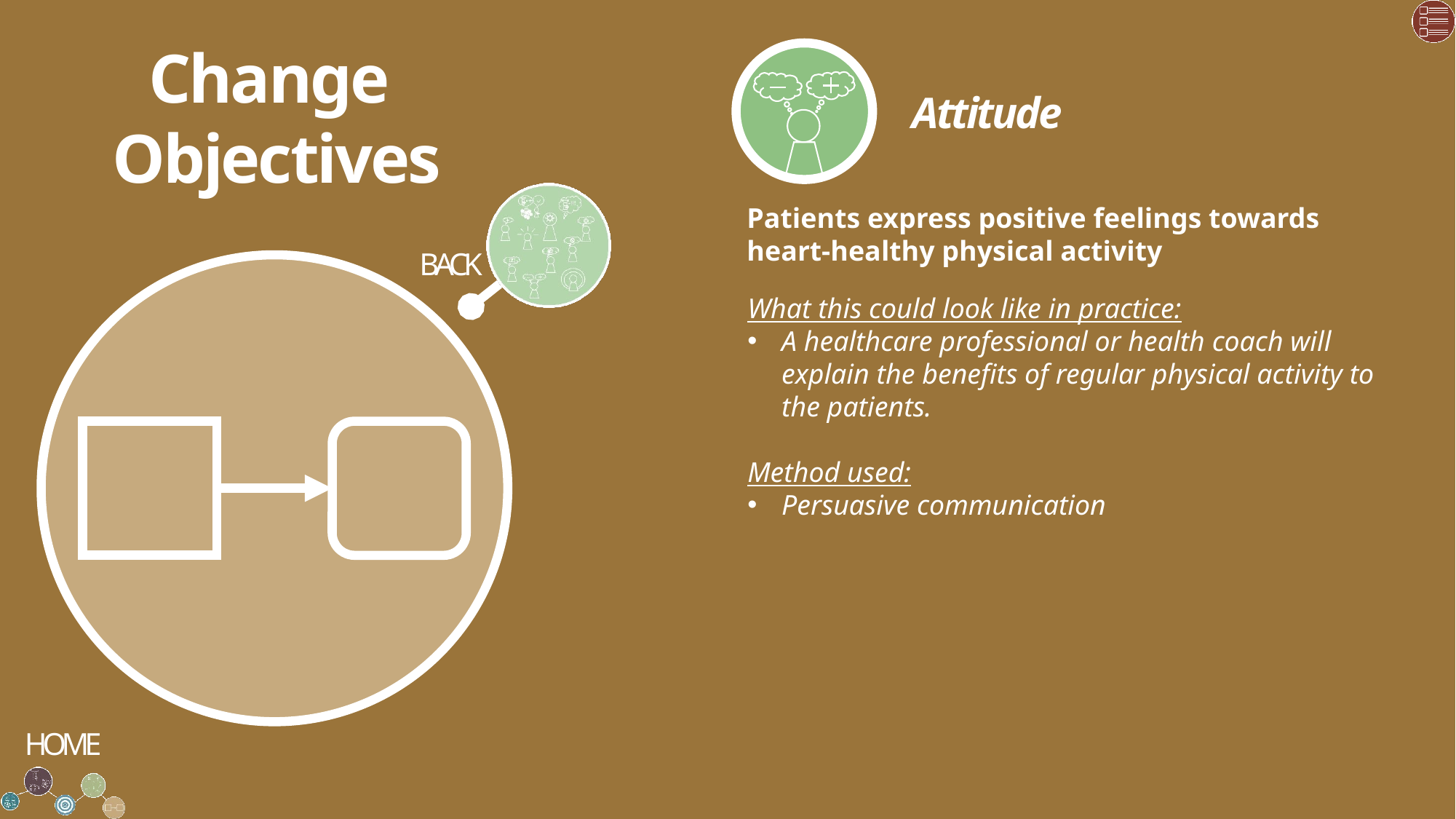

PO2 Att Change Objectives for PO2 for Patients
Change
Objectives
Attitude
Patients express positive feelings towards heart-healthy physical activity
BACK
What this could look like in practice:
A healthcare professional or health coach will explain the benefits of regular physical activity to the patients.
Method used:
Persuasive communication
HOME

## Slide 25
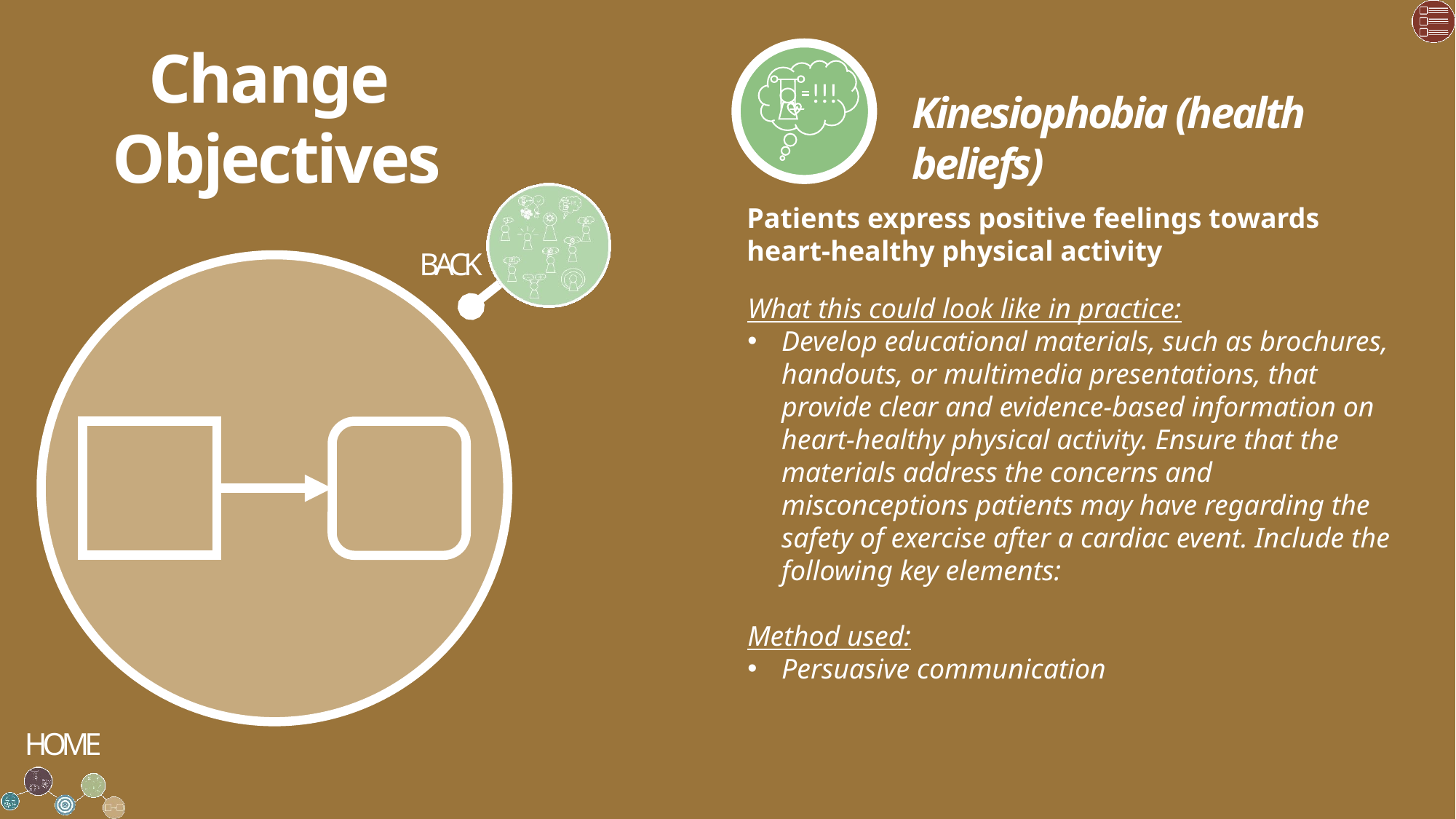

PO2 KHB Change Objectives for PO2 for Patients
Change
Objectives
!!!
Kinesiophobia (health beliefs)
Patients express positive feelings towards heart-healthy physical activity
BACK
What this could look like in practice:
Develop educational materials, such as brochures, handouts, or multimedia presentations, that provide clear and evidence-based information on heart-healthy physical activity. Ensure that the materials address the concerns and misconceptions patients may have regarding the safety of exercise after a cardiac event. Include the following key elements:
Method used:
Persuasive communication
HOME

## Slide 26
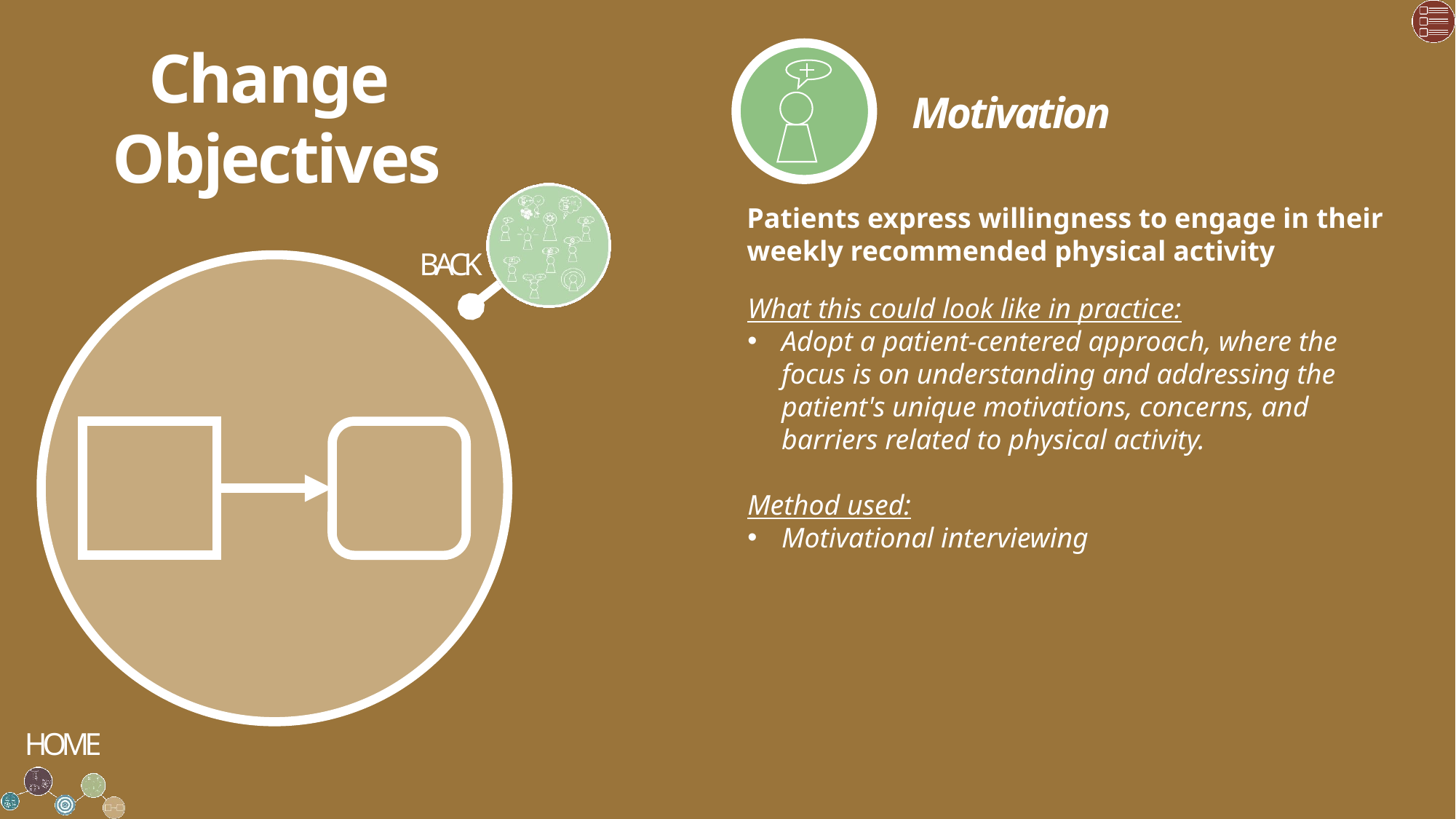

PO2 M Change Objectives for PO2 for Patients
Change
Objectives
Motivation
Patients express willingness to engage in their weekly recommended physical activity
BACK
What this could look like in practice:
Adopt a patient-centered approach, where the focus is on understanding and addressing the patient's unique motivations, concerns, and barriers related to physical activity.
Method used:
Motivational interviewing
HOME

## Slide 27
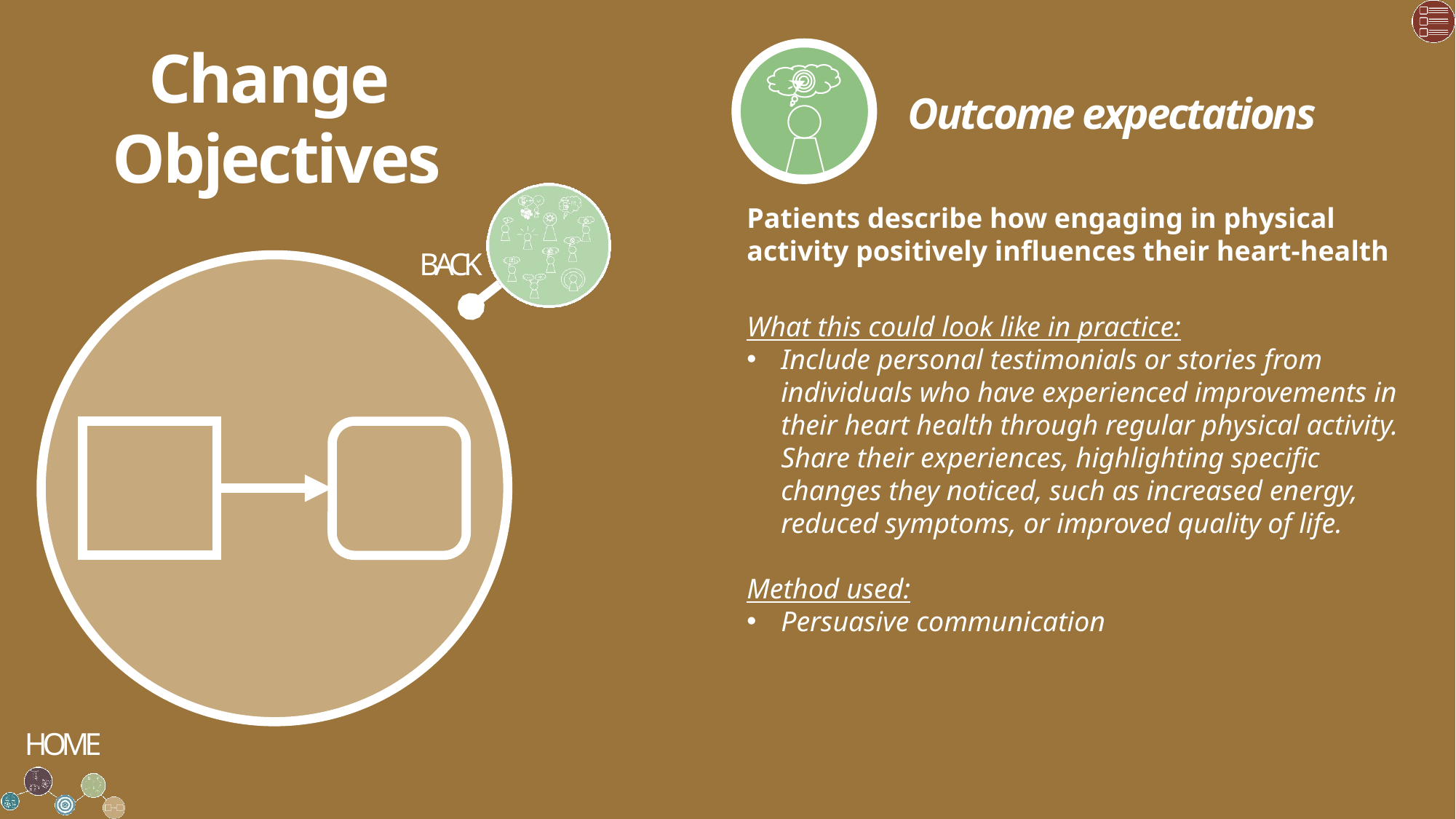

PO2 OE Change Objectives for PO2 for Patients
Change
Objectives
Outcome expectations
Patients describe how engaging in physical activity positively influences their heart-health
BACK
What this could look like in practice:
Include personal testimonials or stories from individuals who have experienced improvements in their heart health through regular physical activity. Share their experiences, highlighting specific changes they noticed, such as increased energy, reduced symptoms, or improved quality of life.
Method used:
Persuasive communication
HOME

## Slide 28
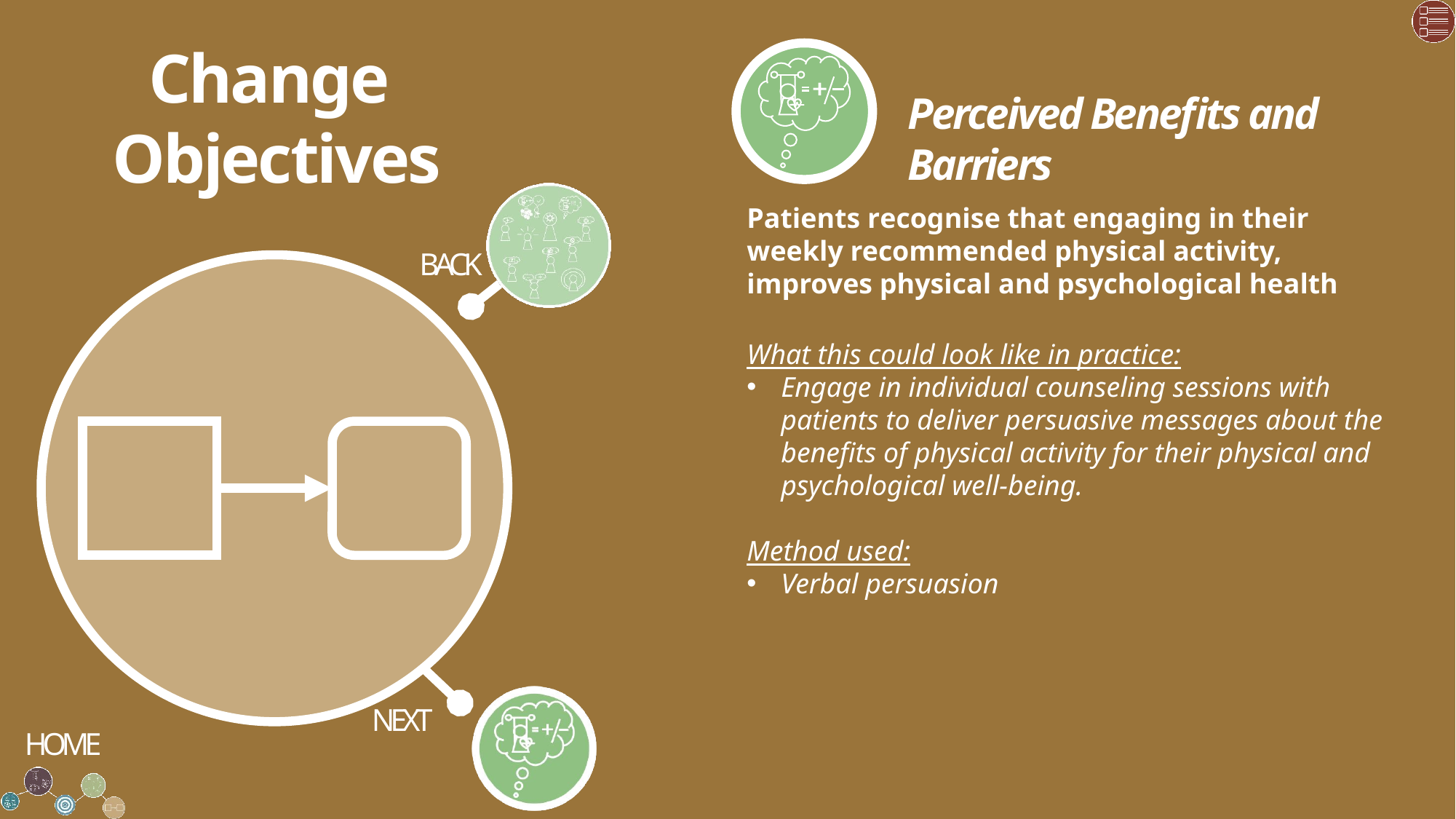

PO2 PB&B Change Objectives for PO2 for Patients
Change
Objectives
Perceived Benefits and Barriers
Patients recognise that engaging in their weekly recommended physical activity, improves physical and psychological health
BACK
What this could look like in practice:
Engage in individual counseling sessions with patients to deliver persuasive messages about the benefits of physical activity for their physical and psychological well-being.
Method used:
Verbal persuasion
NEXT
HOME

## Slide 29
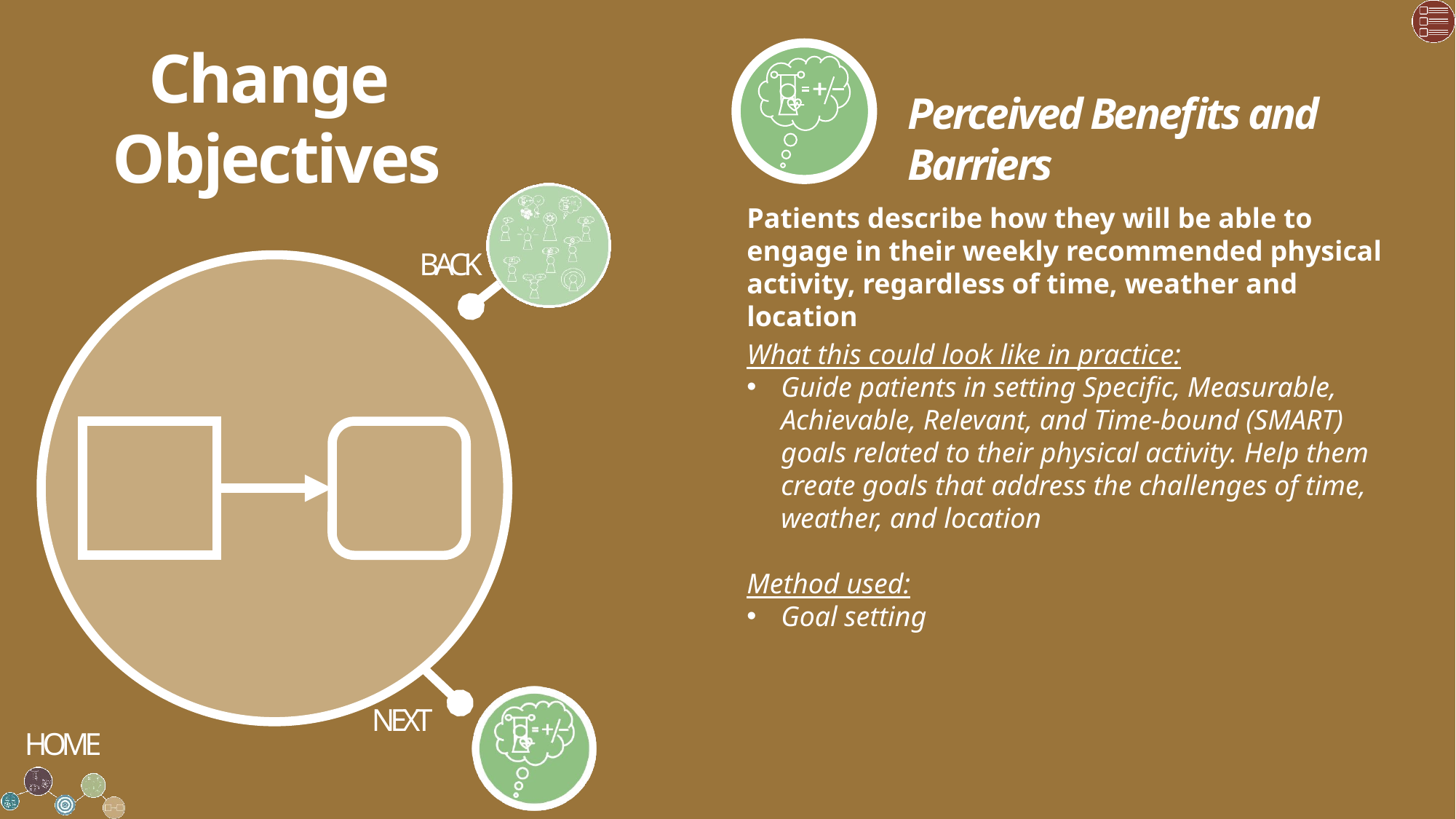

PO2 PB&B Change Objectives for PO2 for Patients
Change
Objectives
Perceived Benefits and Barriers
Patients describe how they will be able to engage in their weekly recommended physical activity, regardless of time, weather and location
BACK
What this could look like in practice:
Guide patients in setting Specific, Measurable, Achievable, Relevant, and Time-bound (SMART) goals related to their physical activity. Help them create goals that address the challenges of time, weather, and location
Method used:
Goal setting
NEXT
HOME

## Slide 30
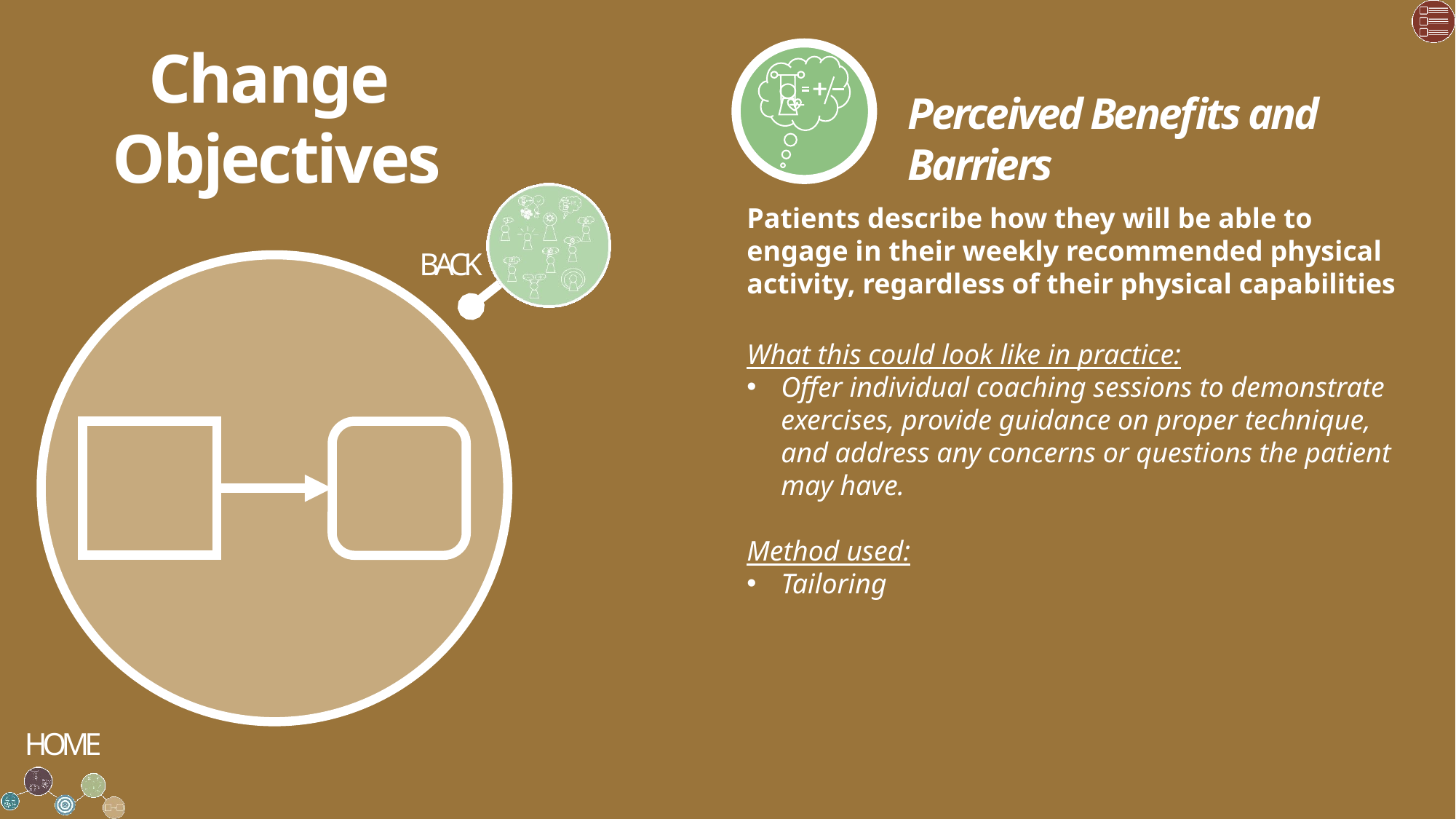

PO2 PB&B Change Objectives for PO2 for Patients
Change
Objectives
Perceived Benefits and Barriers
Patients describe how they will be able to engage in their weekly recommended physical activity, regardless of their physical capabilities
BACK
What this could look like in practice:
Offer individual coaching sessions to demonstrate exercises, provide guidance on proper technique, and address any concerns or questions the patient may have.
Method used:
Tailoring
HOME

## Slide 31
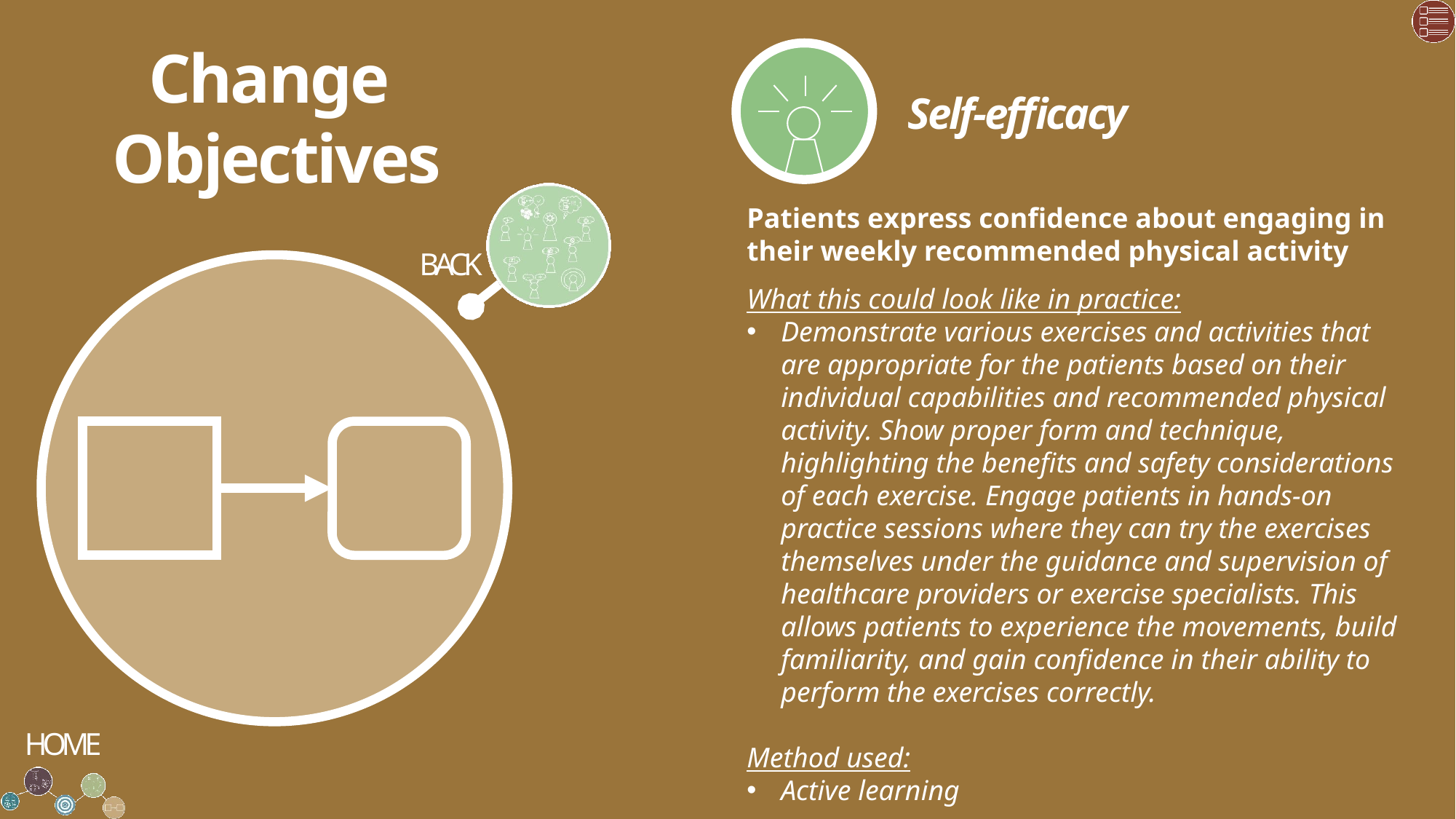

PO2 SE Change Objectives for PO2 for Patients
Change
Objectives
Self-efficacy
Patients express confidence about engaging in their weekly recommended physical activity
BACK
What this could look like in practice:
Demonstrate various exercises and activities that are appropriate for the patients based on their individual capabilities and recommended physical activity. Show proper form and technique, highlighting the benefits and safety considerations of each exercise. Engage patients in hands-on practice sessions where they can try the exercises themselves under the guidance and supervision of healthcare providers or exercise specialists. This allows patients to experience the movements, build familiarity, and gain confidence in their ability to perform the exercises correctly.
Method used:
Active learning
HOME

## Slide 32
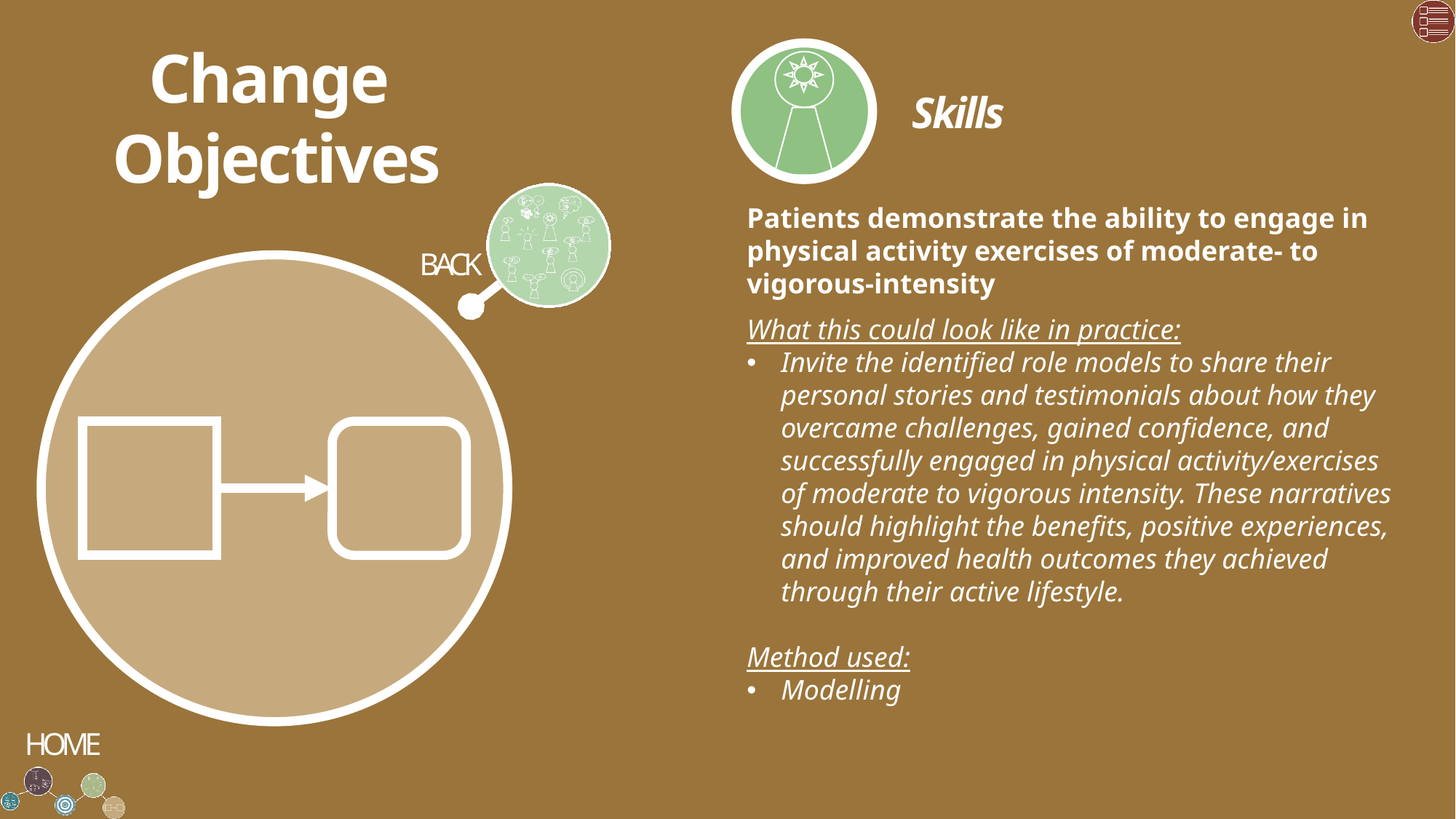

PO2 S Change Objectives for PO2 for Patients
Change
Objectives
Skills
Patients demonstrate the ability to engage in physical activity exercises of moderate- to vigorous-intensity
BACK
What this could look like in practice:
Invite the identified role models to share their personal stories and testimonials about how they overcame challenges, gained confidence, and successfully engaged in physical activity/exercises of moderate to vigorous intensity. These narratives should highlight the benefits, positive experiences, and improved health outcomes they achieved through their active lifestyle.
Method used:
Modelling
HOME

## Slide 33
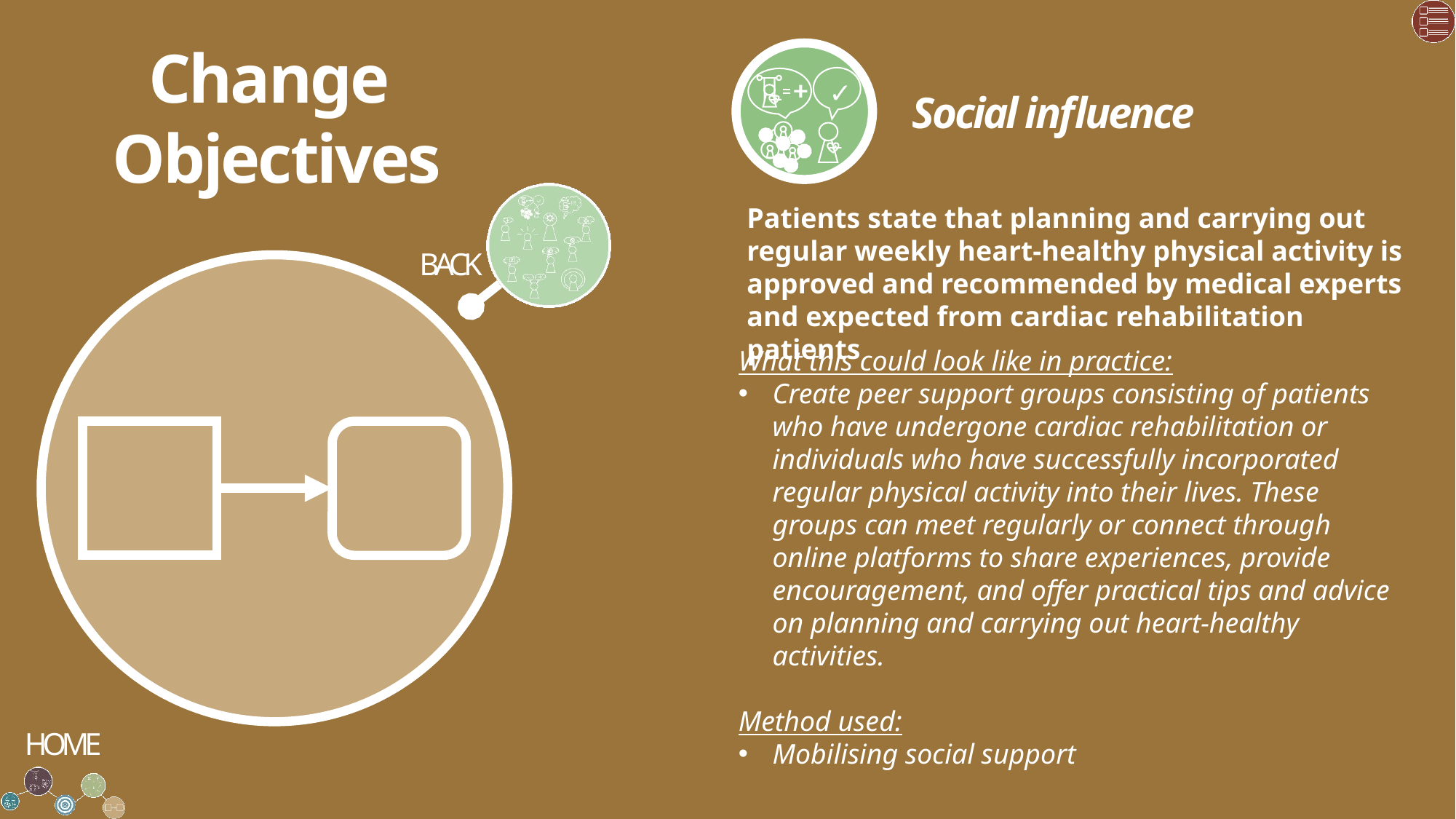

PO2 SI Change Objectives for PO2 for Patients
Change
Objectives
✓
Social influence
Patients state that planning and carrying out regular weekly heart-healthy physical activity is approved and recommended by medical experts and expected from cardiac rehabilitation patients
BACK
What this could look like in practice:
Create peer support groups consisting of patients who have undergone cardiac rehabilitation or individuals who have successfully incorporated regular physical activity into their lives. These groups can meet regularly or connect through online platforms to share experiences, provide encouragement, and offer practical tips and advice on planning and carrying out heart-healthy activities.
Method used:
Mobilising social support
HOME

## Slide 34
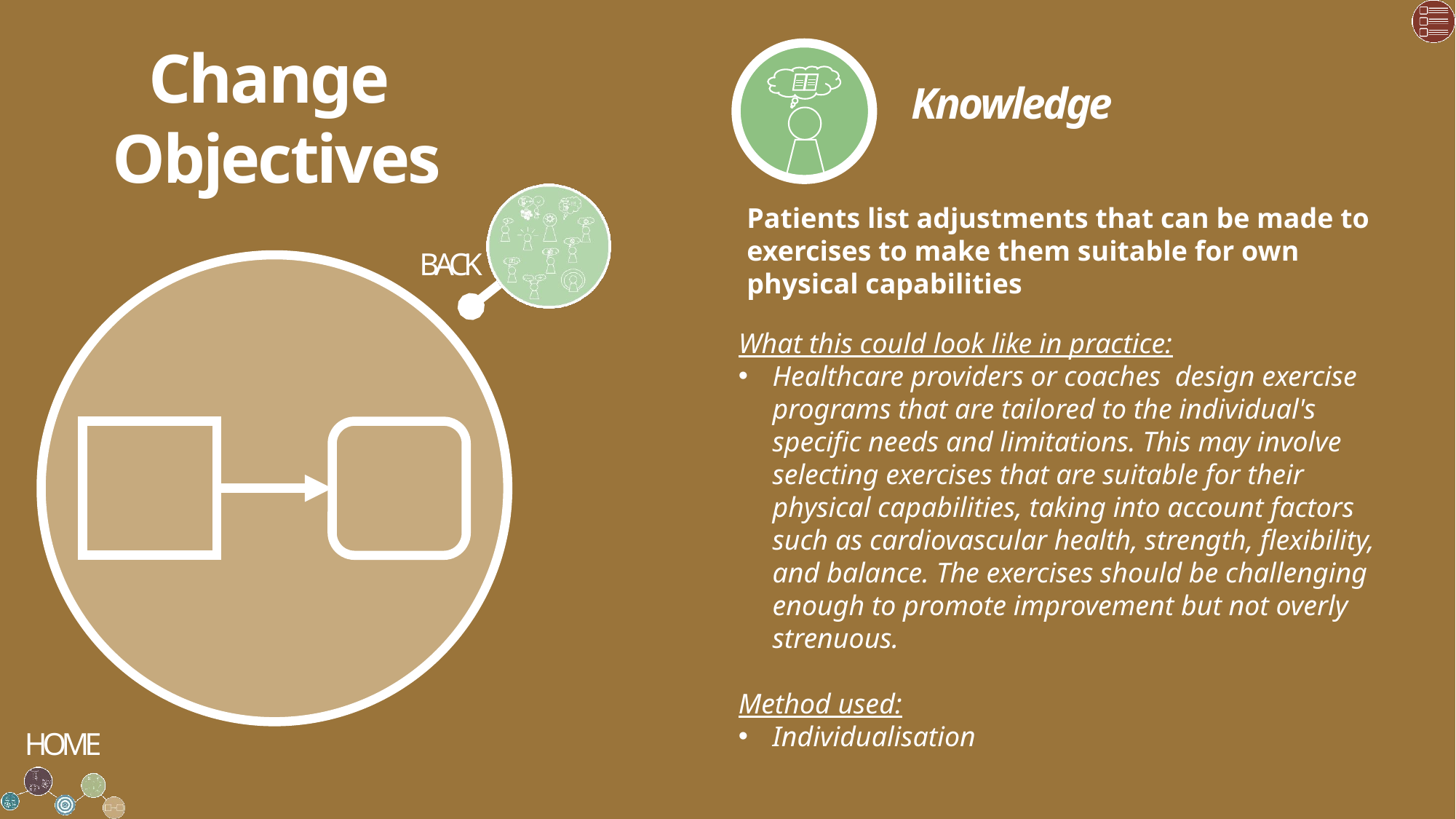

PO3 K Change Objectives for PO3 for Patients
Change
Objectives
Knowledge
Patients list adjustments that can be made to exercises to make them suitable for own physical capabilities
BACK
What this could look like in practice:
Healthcare providers or coaches design exercise programs that are tailored to the individual's specific needs and limitations. This may involve selecting exercises that are suitable for their physical capabilities, taking into account factors such as cardiovascular health, strength, flexibility, and balance. The exercises should be challenging enough to promote improvement but not overly strenuous.
Method used:
Individualisation
HOME

## Slide 35
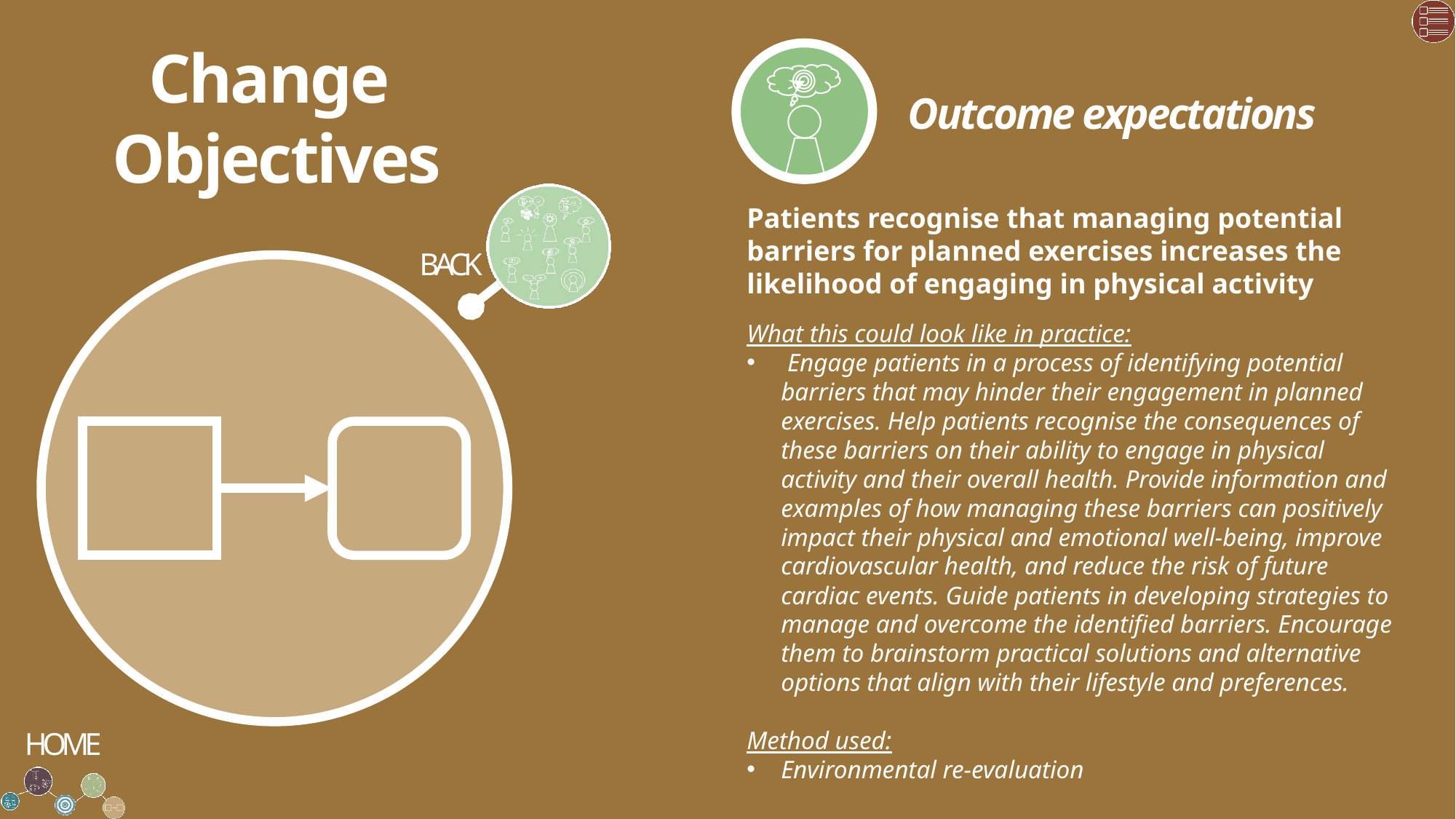

PO3 OE Change Objectives for PO3 for Patients
Change
Objectives
Outcome expectations
Patients recognise that managing potential barriers for planned exercises increases the likelihood of engaging in physical activity
BACK
What this could look like in practice:
 Engage patients in a process of identifying potential barriers that may hinder their engagement in planned exercises. Help patients recognise the consequences of these barriers on their ability to engage in physical activity and their overall health. Provide information and examples of how managing these barriers can positively impact their physical and emotional well-being, improve cardiovascular health, and reduce the risk of future cardiac events. Guide patients in developing strategies to manage and overcome the identified barriers. Encourage them to brainstorm practical solutions and alternative options that align with their lifestyle and preferences.
Method used:
Environmental re-evaluation
HOME

## Slide 36
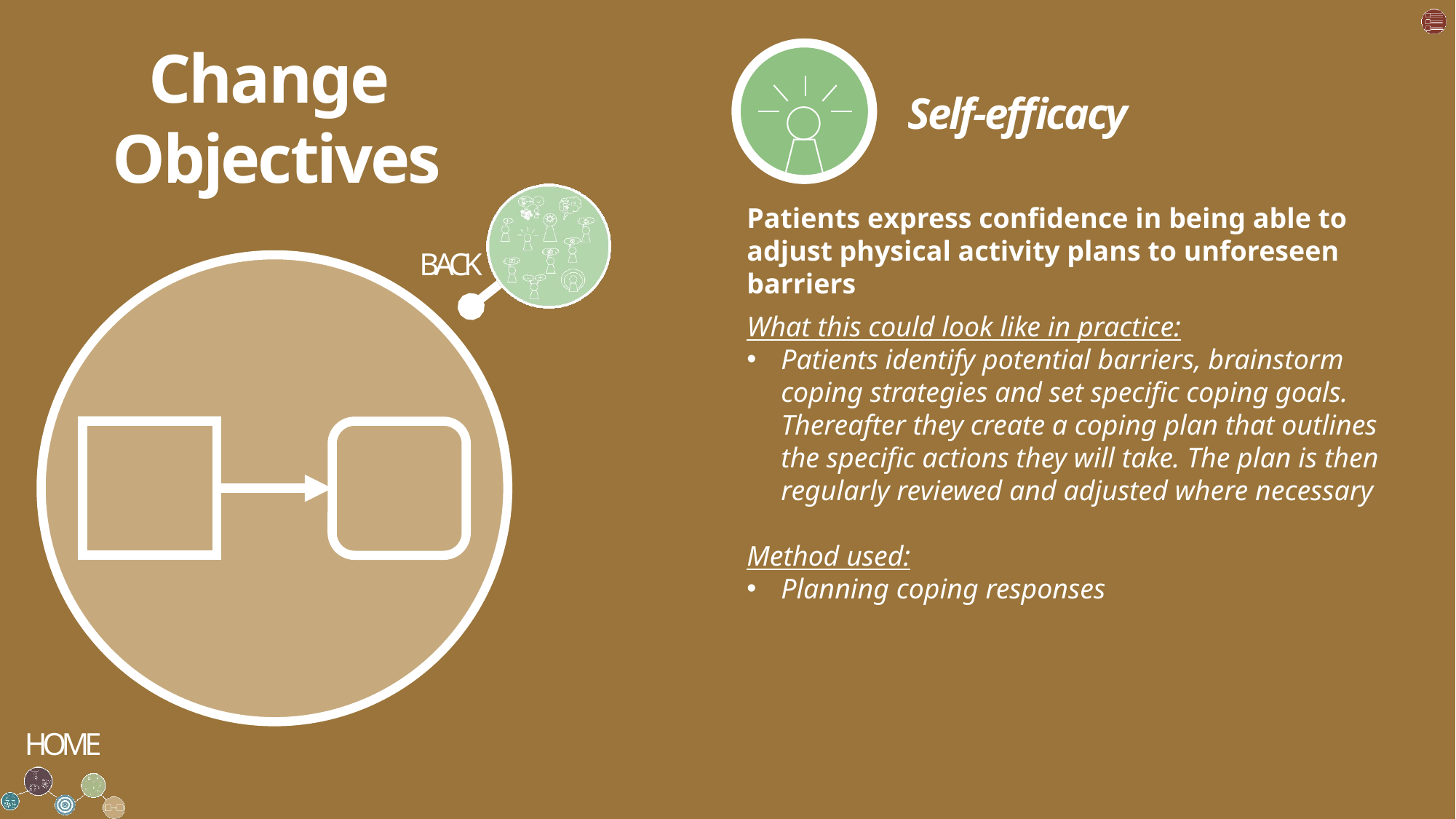

PO3 SE Change Objectives for PO3 for Patients
Change
Objectives
Self-efficacy
Patients express confidence in being able to adjust physical activity plans to unforeseen barriers
BACK
What this could look like in practice:
Patients identify potential barriers, brainstorm coping strategies and set specific coping goals. Thereafter they create a coping plan that outlines the specific actions they will take. The plan is then regularly reviewed and adjusted where necessary
Method used:
Planning coping responses
HOME

## Slide 37
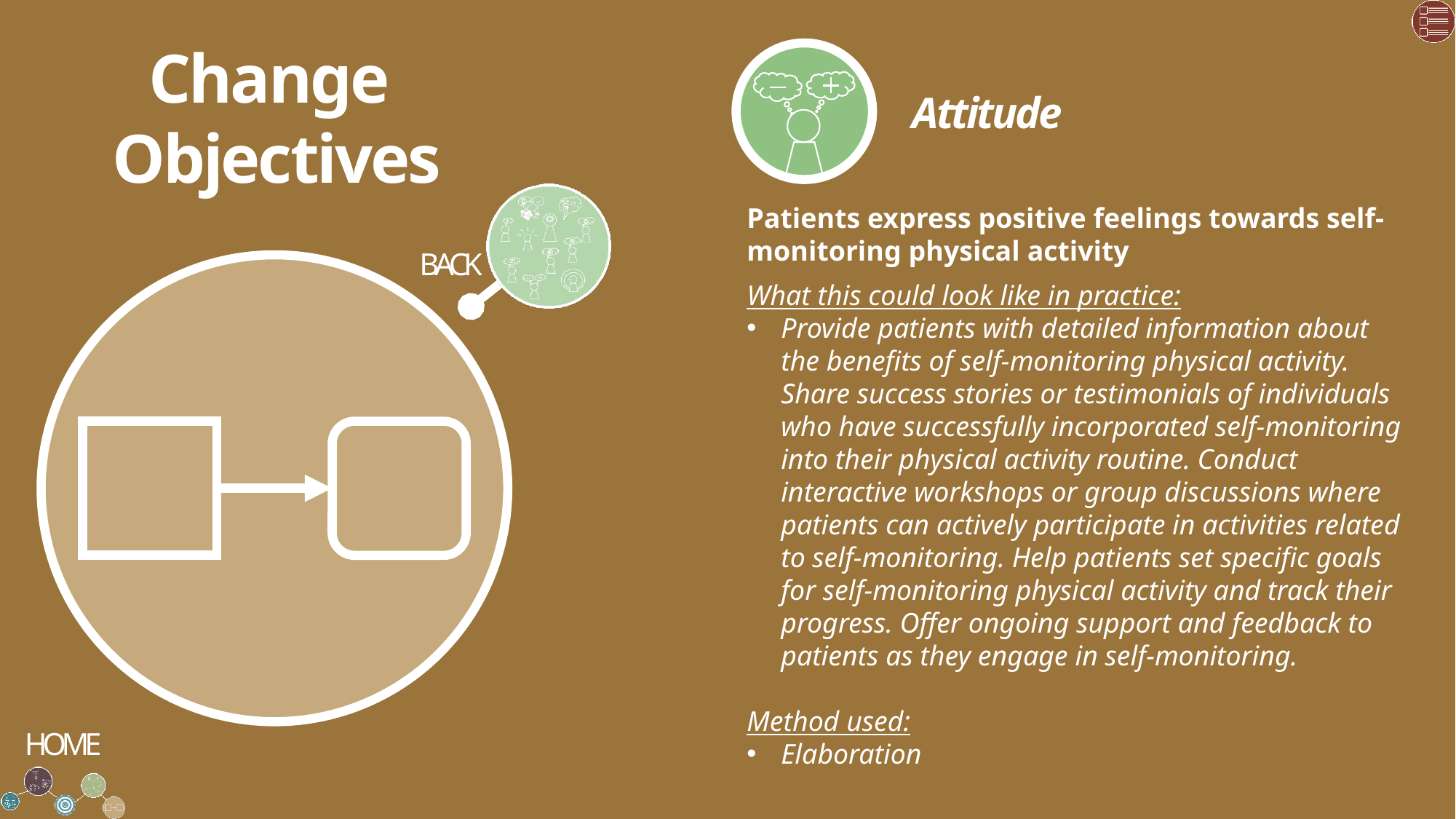

PO4 Att Change Objectives for PO4 for Patients
Change
Objectives
Attitude
Patients express positive feelings towards self-monitoring physical activity
BACK
What this could look like in practice:
Provide patients with detailed information about the benefits of self-monitoring physical activity. Share success stories or testimonials of individuals who have successfully incorporated self-monitoring into their physical activity routine. Conduct interactive workshops or group discussions where patients can actively participate in activities related to self-monitoring. Help patients set specific goals for self-monitoring physical activity and track their progress. Offer ongoing support and feedback to patients as they engage in self-monitoring.
Method used:
Elaboration
HOME

## Slide 38
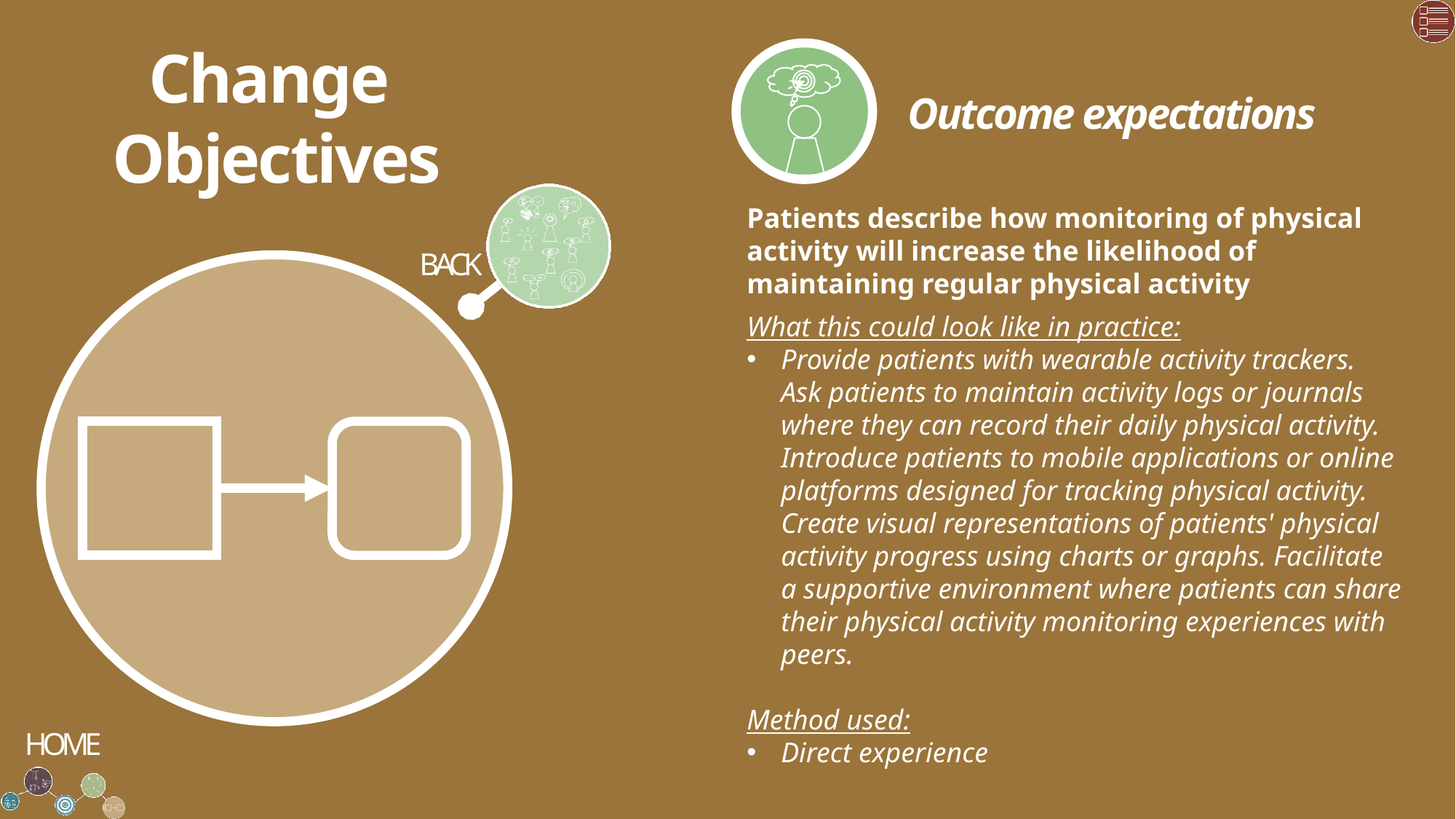

PO4 OE Change Objectives for PO4 for Patients
Change
Objectives
Outcome expectations
Patients describe how monitoring of physical activity will increase the likelihood of maintaining regular physical activity
BACK
What this could look like in practice:
Provide patients with wearable activity trackers. Ask patients to maintain activity logs or journals where they can record their daily physical activity. Introduce patients to mobile applications or online platforms designed for tracking physical activity. Create visual representations of patients' physical activity progress using charts or graphs. Facilitate a supportive environment where patients can share their physical activity monitoring experiences with peers.
Method used:
Direct experience
HOME

## Slide 39
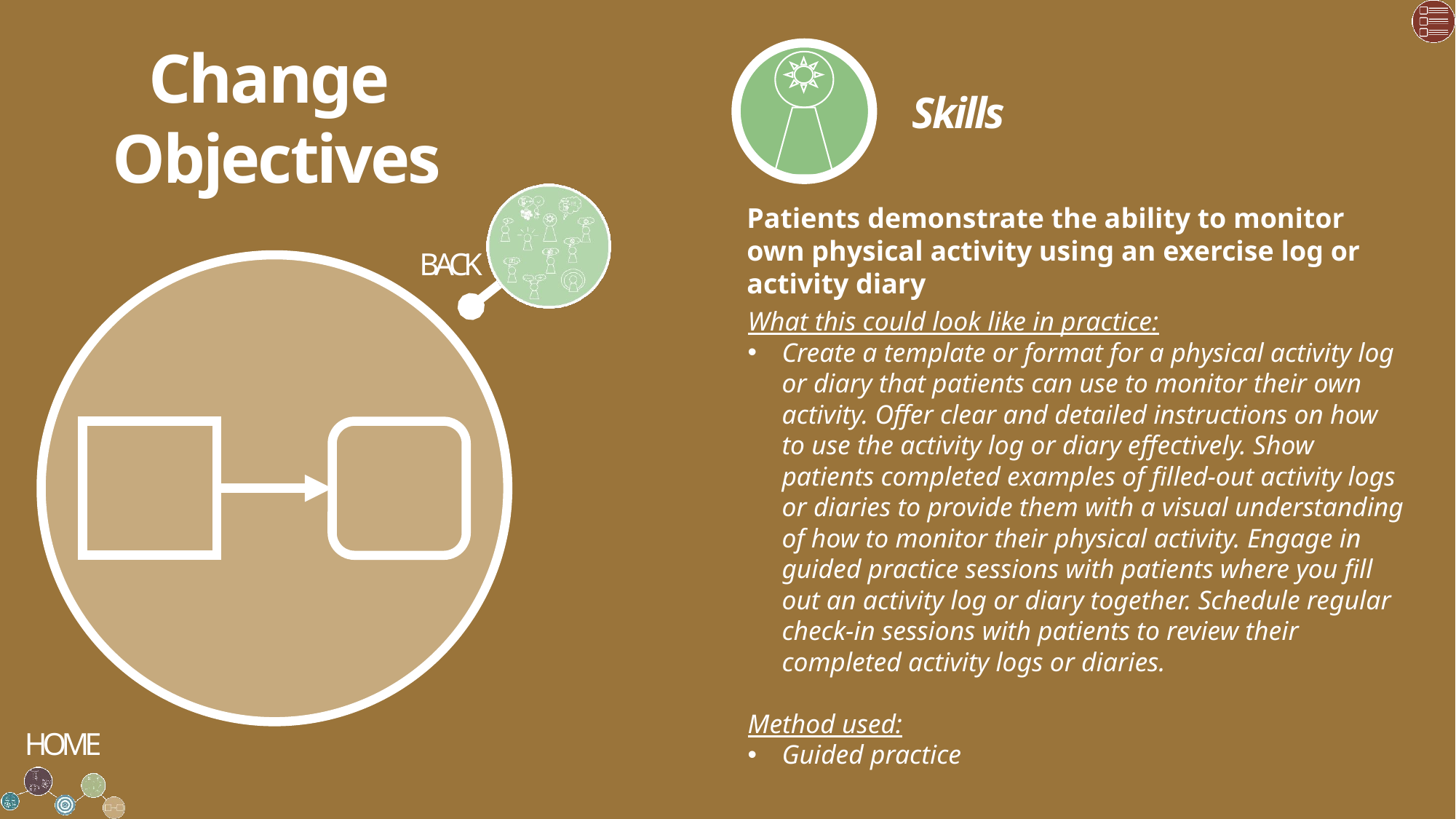

PO4 S Change Objectives for PO4 for Patients
Change
Objectives
Skills
Patients demonstrate the ability to monitor own physical activity using an exercise log or activity diary
BACK
What this could look like in practice:
Create a template or format for a physical activity log or diary that patients can use to monitor their own activity. Offer clear and detailed instructions on how to use the activity log or diary effectively. Show patients completed examples of filled-out activity logs or diaries to provide them with a visual understanding of how to monitor their physical activity. Engage in guided practice sessions with patients where you fill out an activity log or diary together. Schedule regular check-in sessions with patients to review their completed activity logs or diaries.
Method used:
Guided practice
HOME

## Slide 40
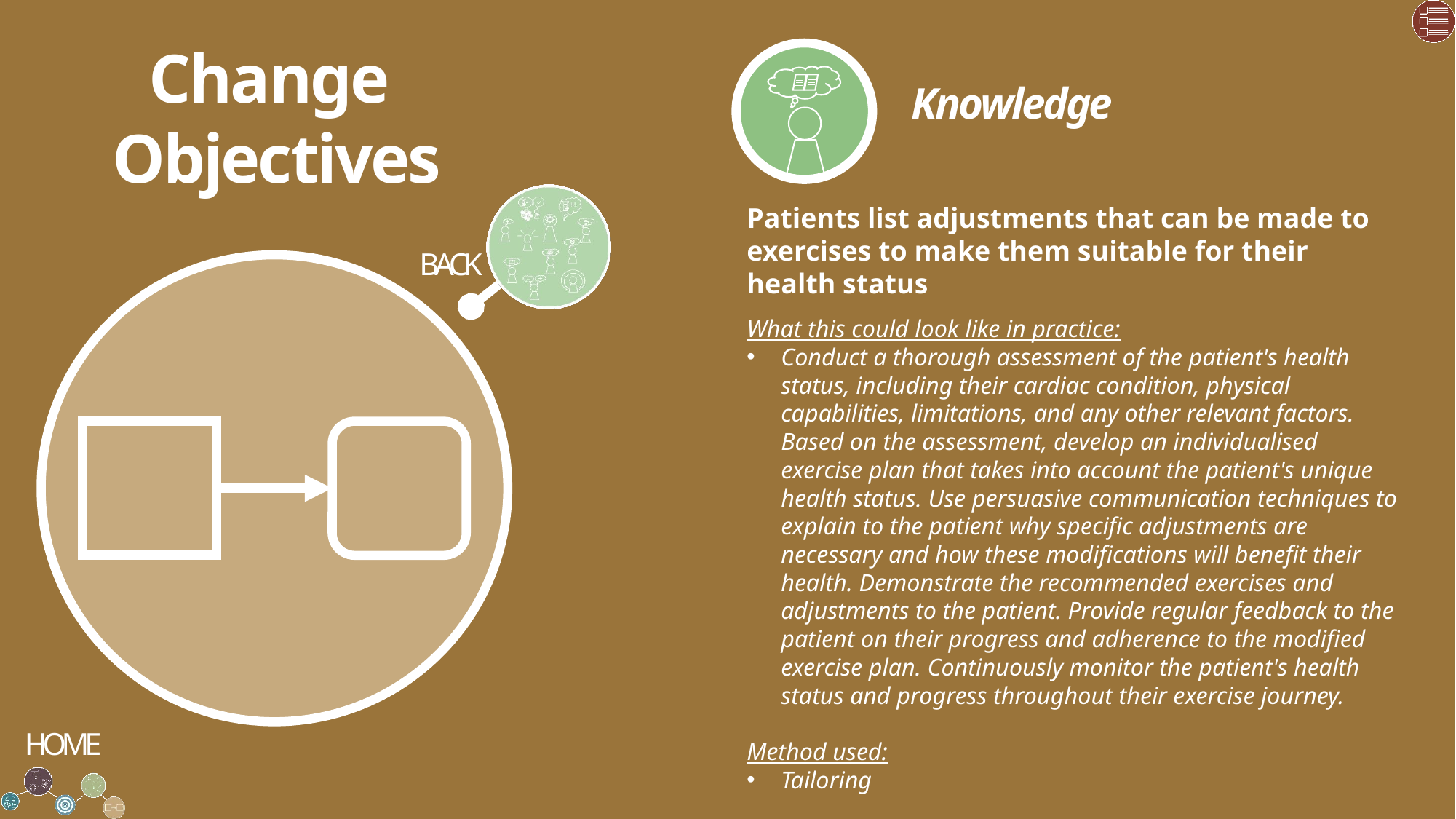

PO5 K Change Objectives for PO5 for Patients
Change
Objectives
Knowledge
Patients list adjustments that can be made to exercises to make them suitable for their health status
BACK
What this could look like in practice:
Conduct a thorough assessment of the patient's health status, including their cardiac condition, physical capabilities, limitations, and any other relevant factors. Based on the assessment, develop an individualised exercise plan that takes into account the patient's unique health status. Use persuasive communication techniques to explain to the patient why specific adjustments are necessary and how these modifications will benefit their health. Demonstrate the recommended exercises and adjustments to the patient. Provide regular feedback to the patient on their progress and adherence to the modified exercise plan. Continuously monitor the patient's health status and progress throughout their exercise journey.
Method used:
Tailoring
HOME

## Slide 41
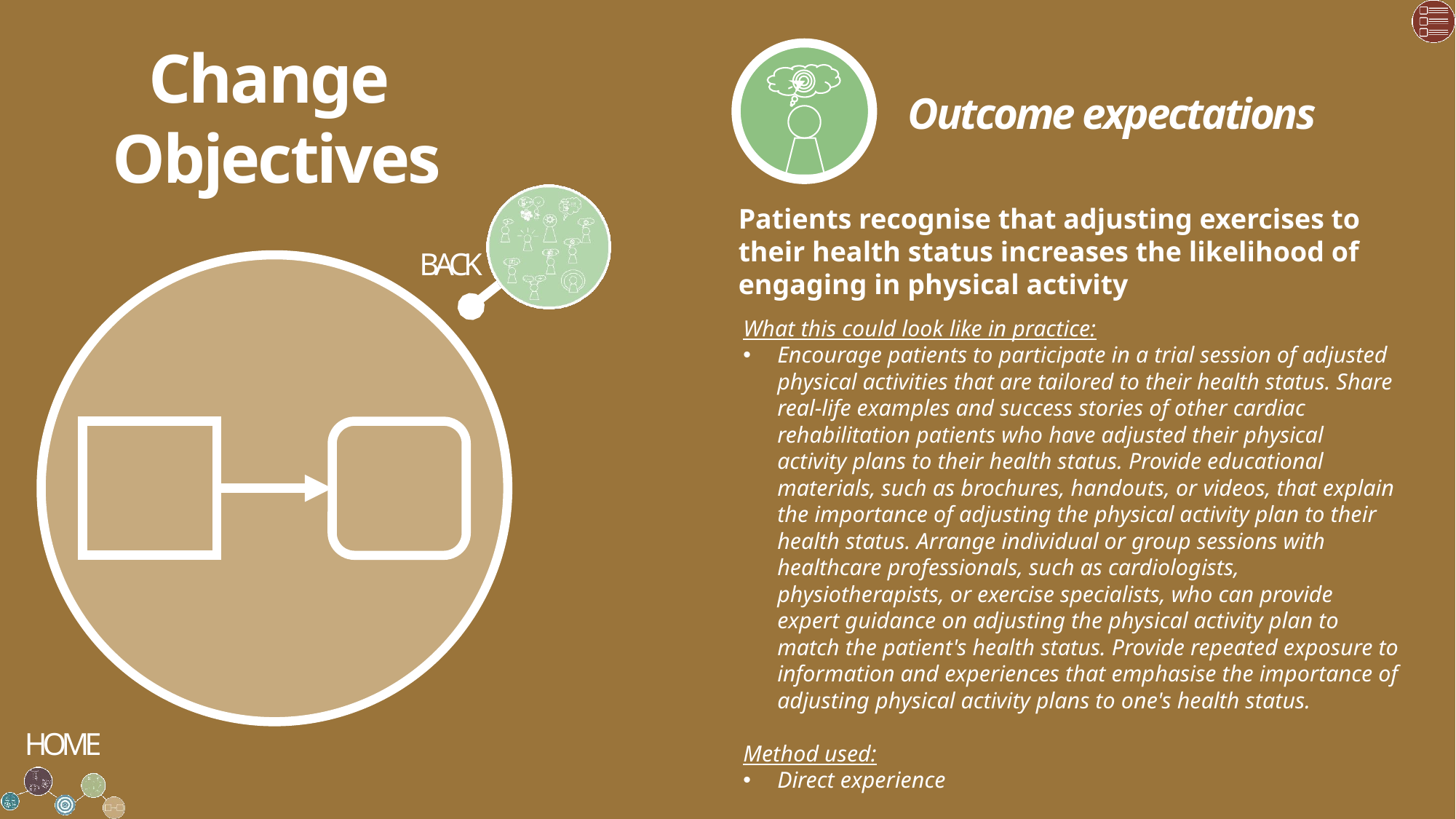

PO5 OE Change Objectives for PO5 for Patients
Change
Objectives
Outcome expectations
Patients recognise that adjusting exercises to their health status increases the likelihood of engaging in physical activity
BACK
What this could look like in practice:
Encourage patients to participate in a trial session of adjusted physical activities that are tailored to their health status. Share real-life examples and success stories of other cardiac rehabilitation patients who have adjusted their physical activity plans to their health status. Provide educational materials, such as brochures, handouts, or videos, that explain the importance of adjusting the physical activity plan to their health status. Arrange individual or group sessions with healthcare professionals, such as cardiologists, physiotherapists, or exercise specialists, who can provide expert guidance on adjusting the physical activity plan to match the patient's health status. Provide repeated exposure to information and experiences that emphasise the importance of adjusting physical activity plans to one's health status.
Method used:
Direct experience
HOME

## Slide 42
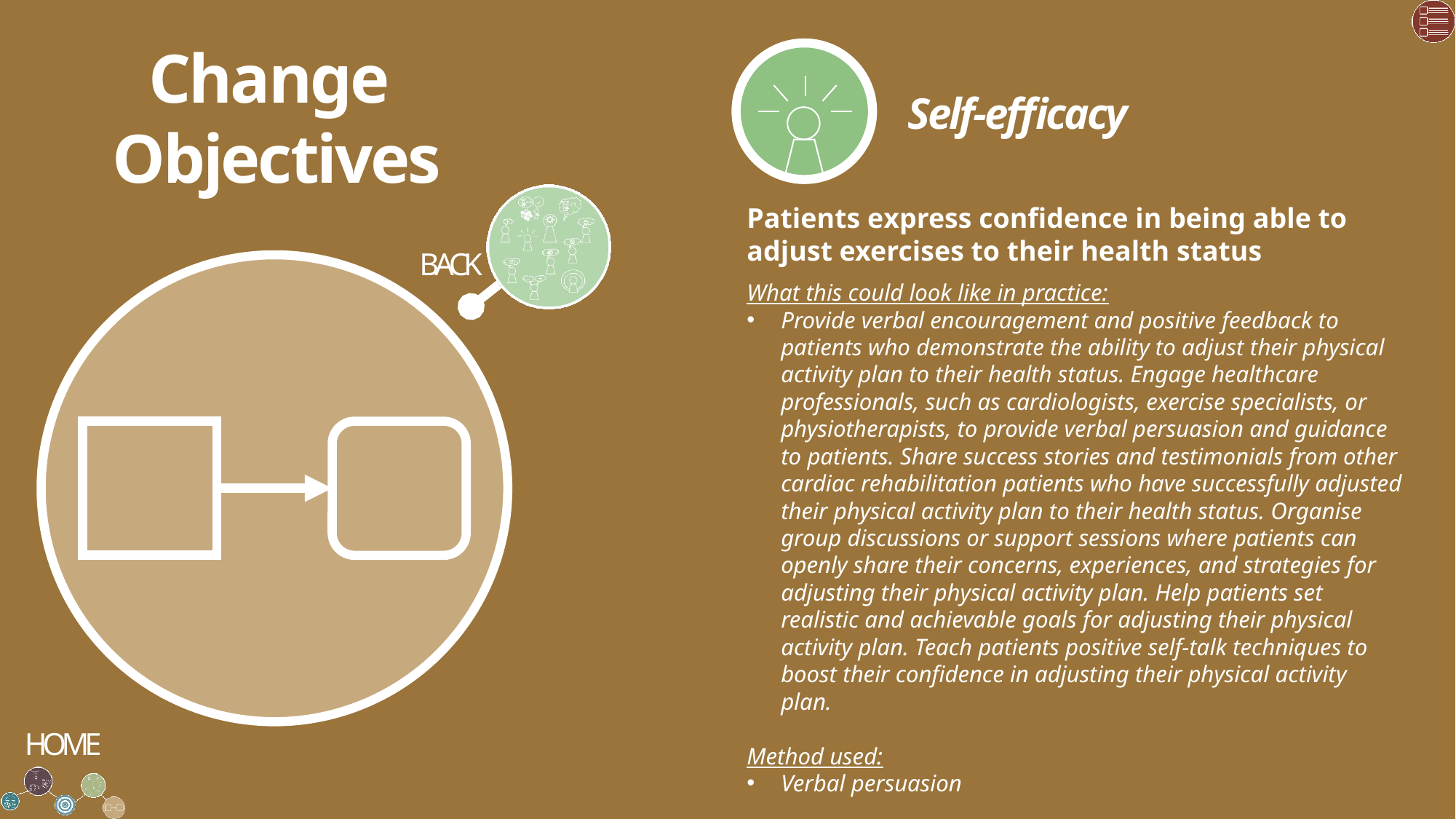

PO5 SE Change Objectives for PO5 for Patients
Change
Objectives
Self-efficacy
Patients express confidence in being able to adjust exercises to their health status
BACK
What this could look like in practice:
Provide verbal encouragement and positive feedback to patients who demonstrate the ability to adjust their physical activity plan to their health status. Engage healthcare professionals, such as cardiologists, exercise specialists, or physiotherapists, to provide verbal persuasion and guidance to patients. Share success stories and testimonials from other cardiac rehabilitation patients who have successfully adjusted their physical activity plan to their health status. Organise group discussions or support sessions where patients can openly share their concerns, experiences, and strategies for adjusting their physical activity plan. Help patients set realistic and achievable goals for adjusting their physical activity plan. Teach patients positive self-talk techniques to boost their confidence in adjusting their physical activity plan.
Method used:
Verbal persuasion
HOME

## Slide 43
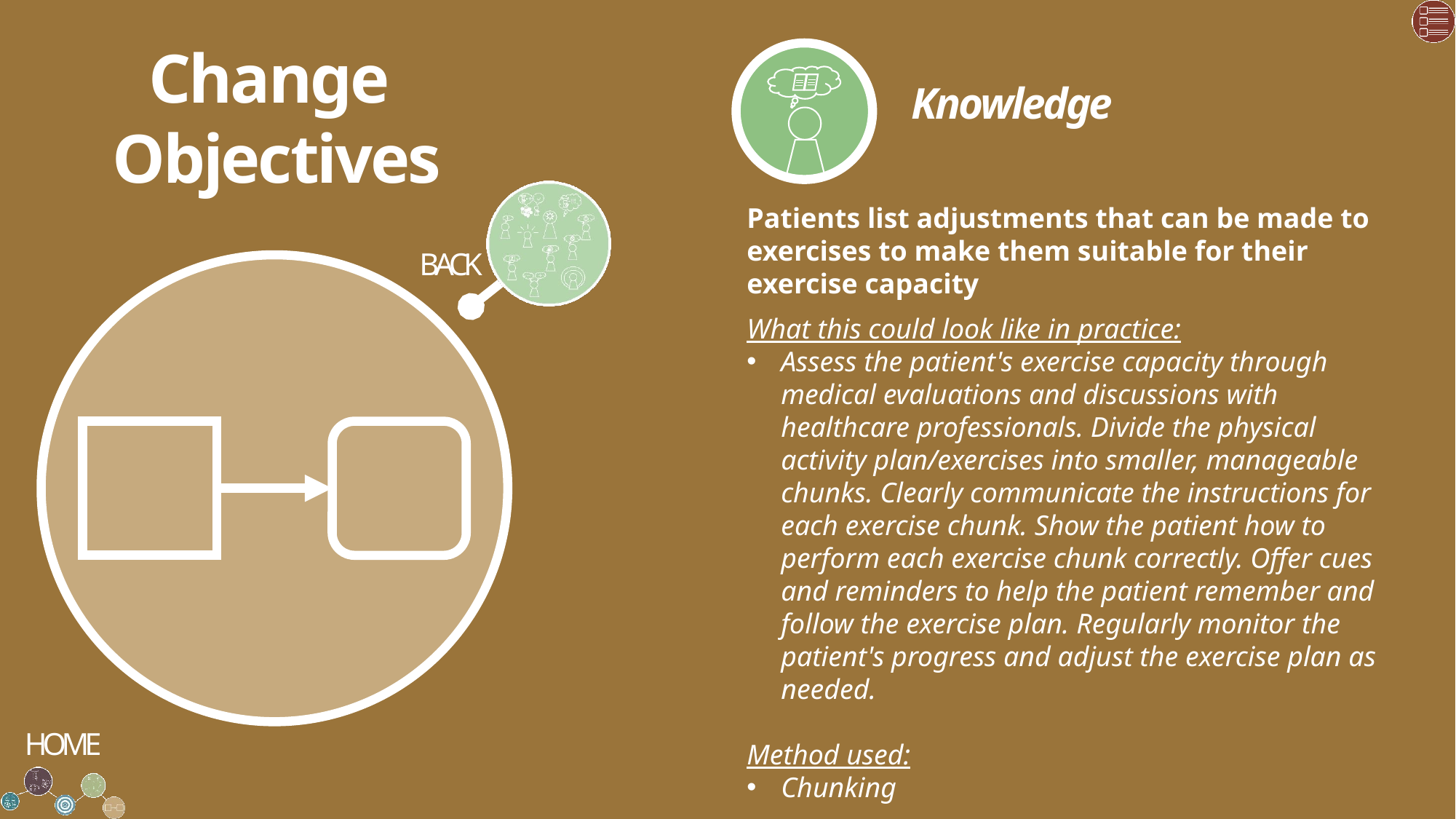

PO6 K Change Objectives for PO6 for Patients
Change
Objectives
Knowledge
Patients list adjustments that can be made to exercises to make them suitable for their exercise capacity
BACK
What this could look like in practice:
Assess the patient's exercise capacity through medical evaluations and discussions with healthcare professionals. Divide the physical activity plan/exercises into smaller, manageable chunks. Clearly communicate the instructions for each exercise chunk. Show the patient how to perform each exercise chunk correctly. Offer cues and reminders to help the patient remember and follow the exercise plan. Regularly monitor the patient's progress and adjust the exercise plan as needed.
Method used:
Chunking
HOME

## Slide 44
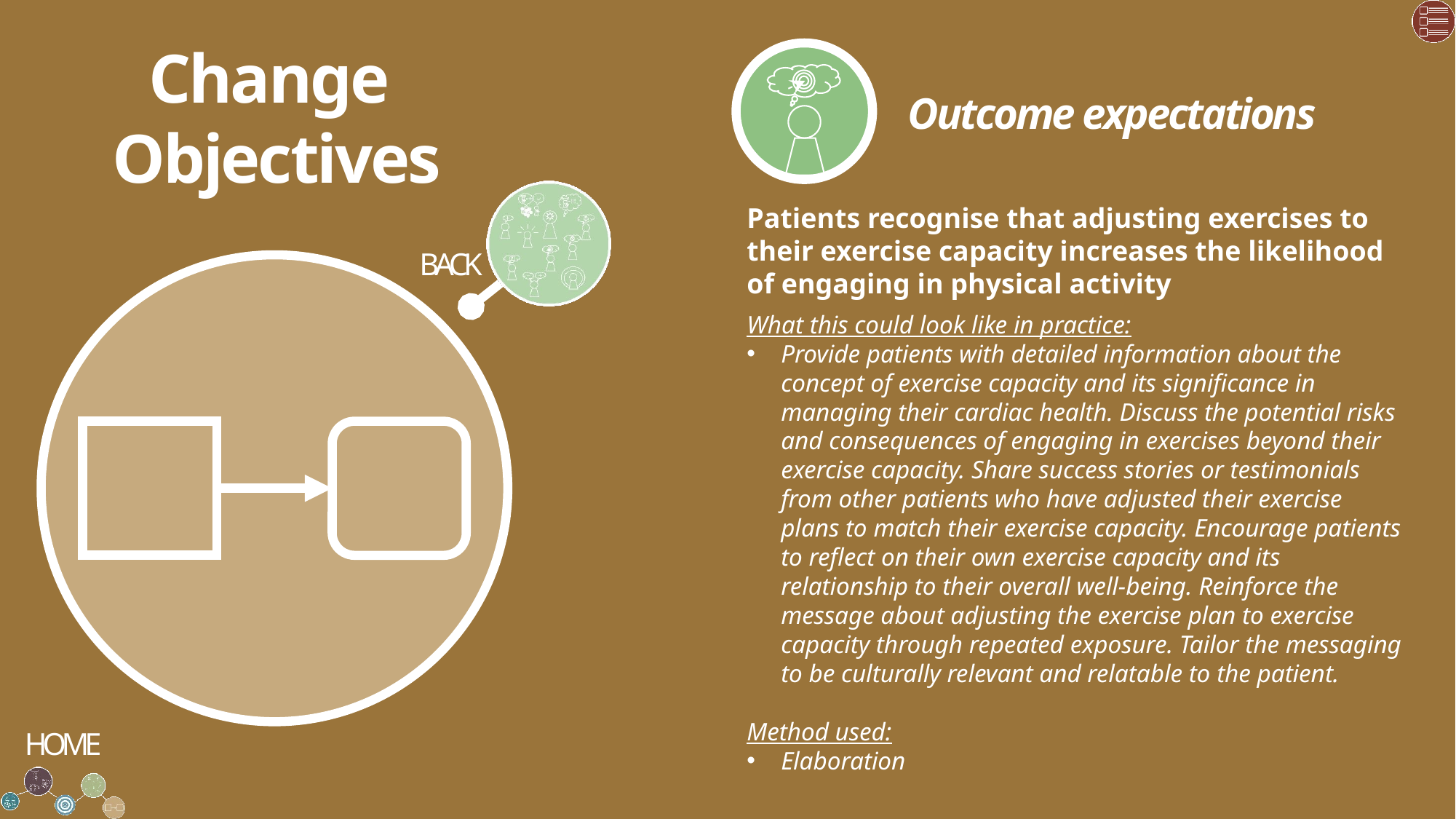

PO6 OE Change Objectives for PO6 for Patients
Change
Objectives
Outcome expectations
Patients recognise that adjusting exercises to their exercise capacity increases the likelihood of engaging in physical activity
BACK
What this could look like in practice:
Provide patients with detailed information about the concept of exercise capacity and its significance in managing their cardiac health. Discuss the potential risks and consequences of engaging in exercises beyond their exercise capacity. Share success stories or testimonials from other patients who have adjusted their exercise plans to match their exercise capacity. Encourage patients to reflect on their own exercise capacity and its relationship to their overall well-being. Reinforce the message about adjusting the exercise plan to exercise capacity through repeated exposure. Tailor the messaging to be culturally relevant and relatable to the patient.
Method used:
Elaboration
HOME

## Slide 45
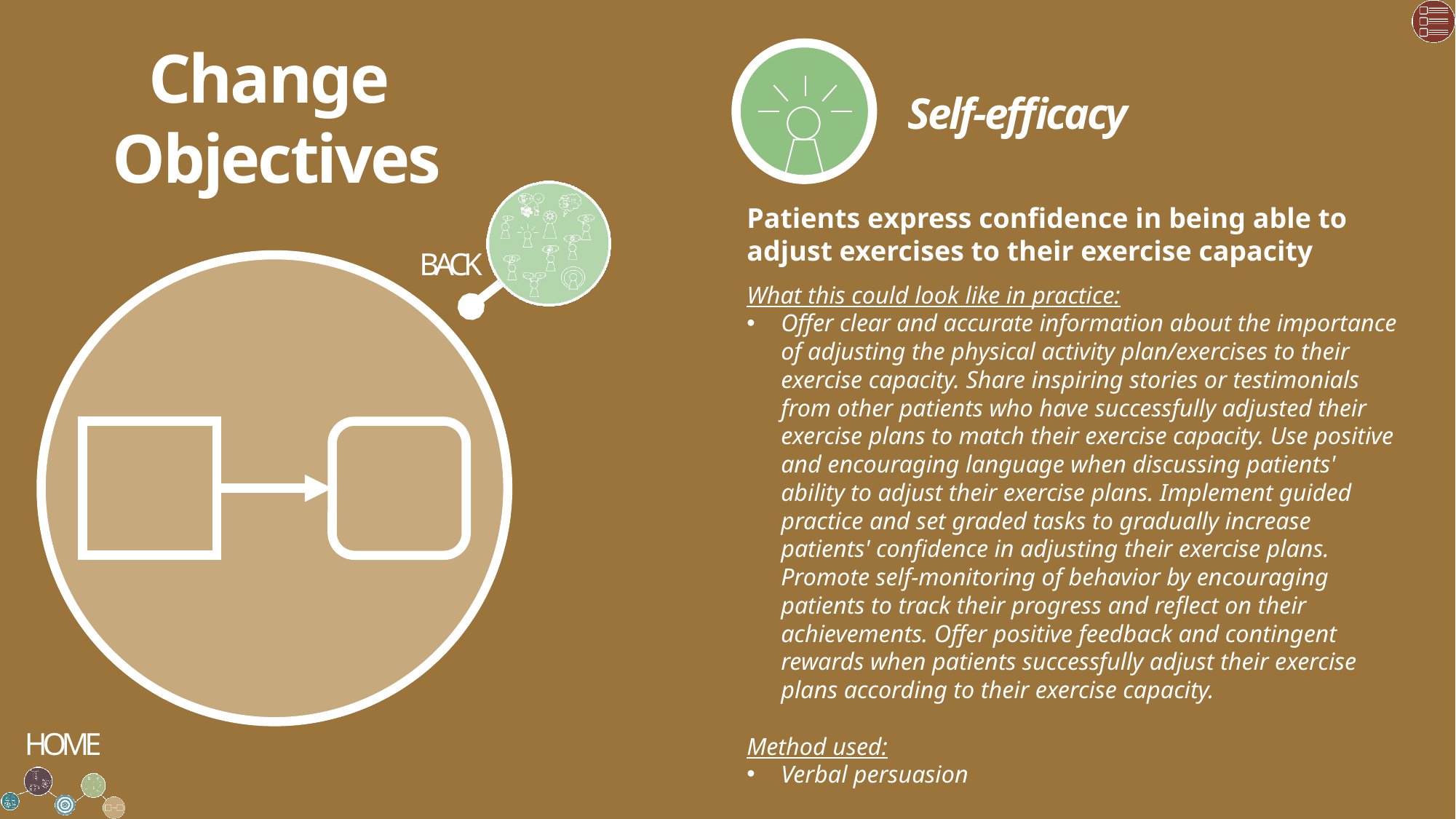

PO6 SE Change Objectives for PO6 for Patients
Change
Objectives
Self-efficacy
Patients express confidence in being able to adjust exercises to their exercise capacity
BACK
What this could look like in practice:
Offer clear and accurate information about the importance of adjusting the physical activity plan/exercises to their exercise capacity. Share inspiring stories or testimonials from other patients who have successfully adjusted their exercise plans to match their exercise capacity. Use positive and encouraging language when discussing patients' ability to adjust their exercise plans. Implement guided practice and set graded tasks to gradually increase patients' confidence in adjusting their exercise plans. Promote self-monitoring of behavior by encouraging patients to track their progress and reflect on their achievements. Offer positive feedback and contingent rewards when patients successfully adjust their exercise plans according to their exercise capacity.
Method used:
Verbal persuasion
HOME

## Slide 46
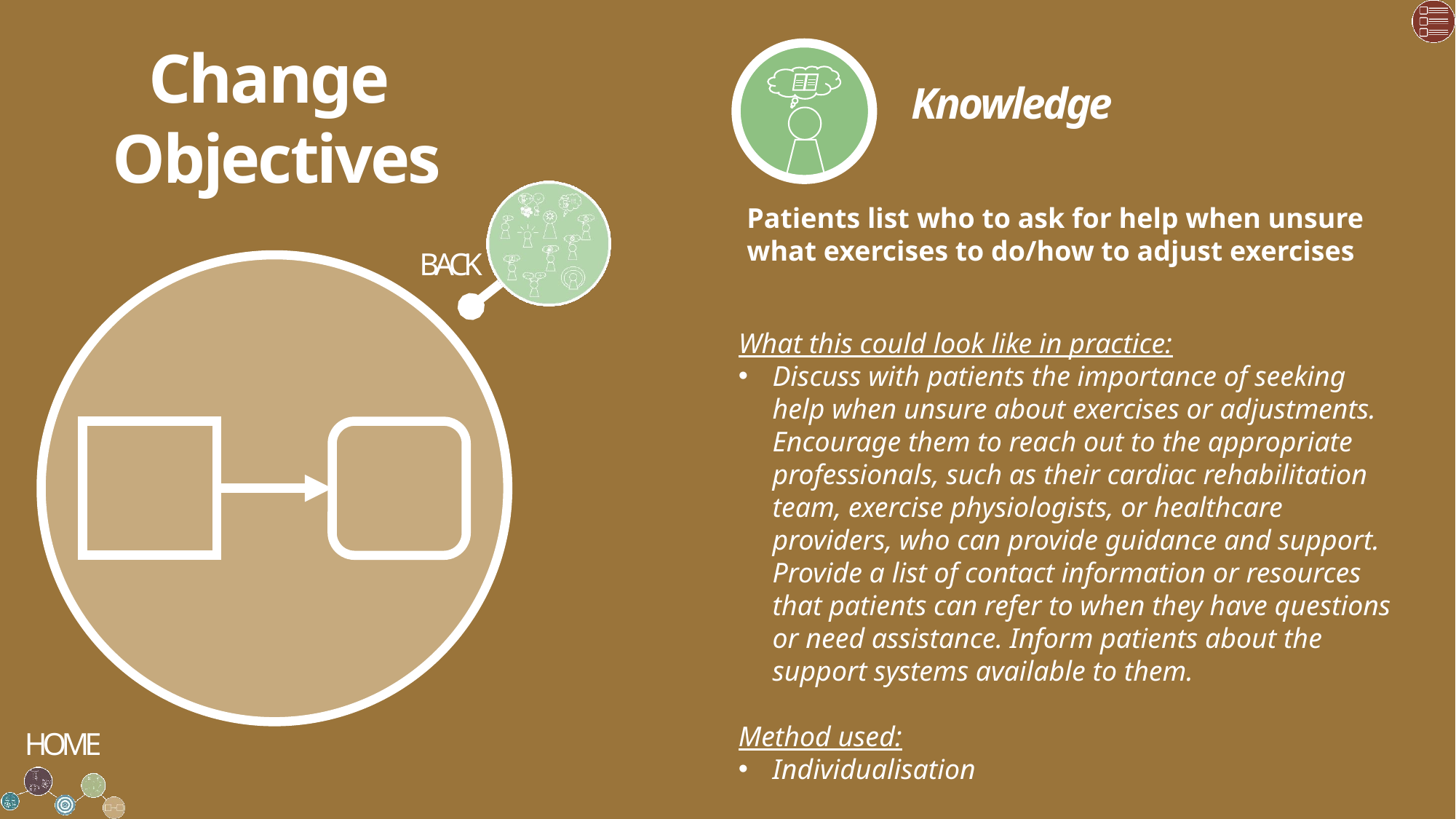

PO7 K Change Objectives for PO7 for Patients
Change
Objectives
Knowledge
Patients list who to ask for help when unsure what exercises to do/how to adjust exercises
BACK
What this could look like in practice:
Discuss with patients the importance of seeking help when unsure about exercises or adjustments. Encourage them to reach out to the appropriate professionals, such as their cardiac rehabilitation team, exercise physiologists, or healthcare providers, who can provide guidance and support. Provide a list of contact information or resources that patients can refer to when they have questions or need assistance. Inform patients about the support systems available to them.
Method used:
Individualisation
HOME

## Slide 47
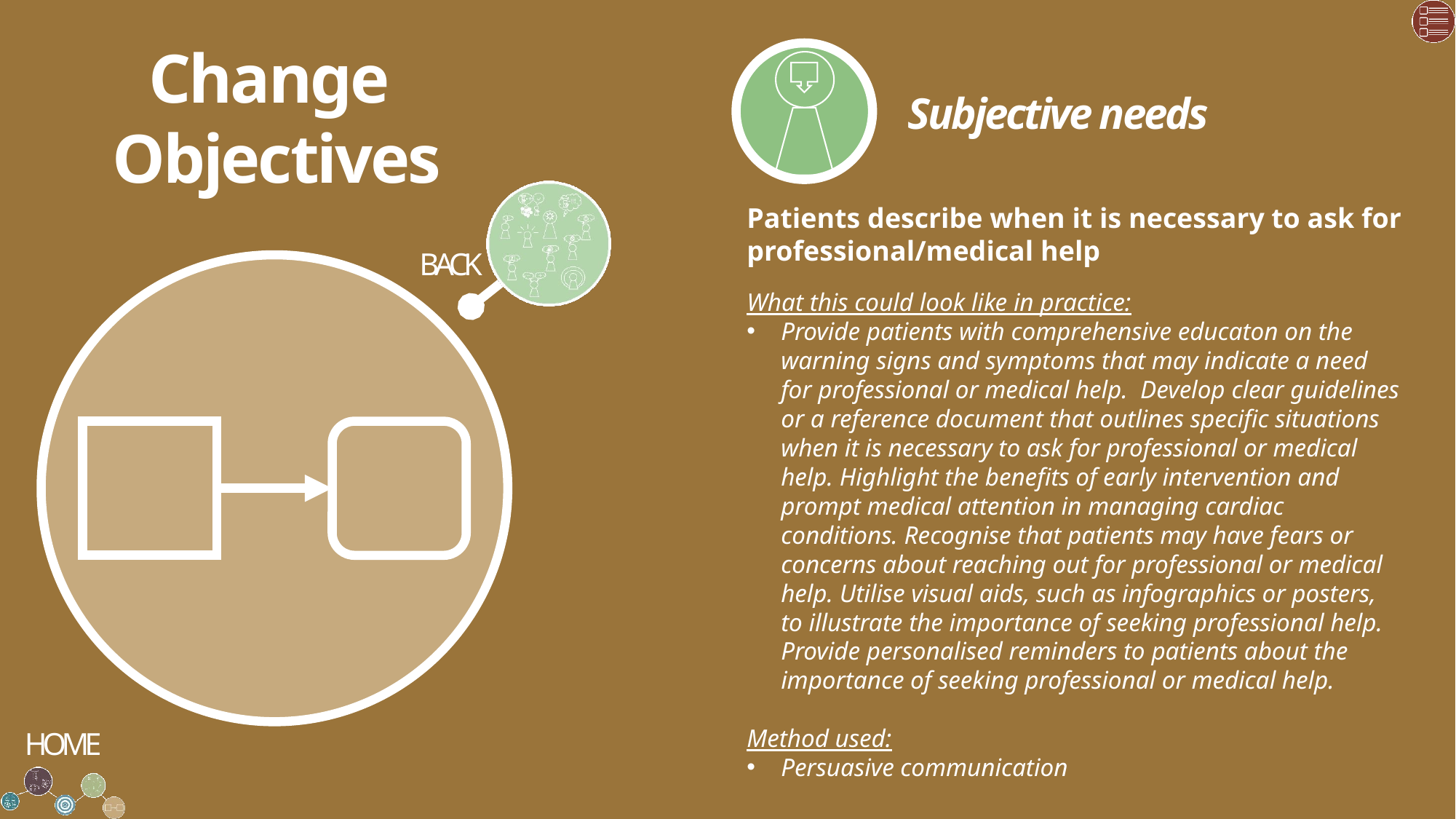

PO7 SNE Change Objectives for PO7 for Patients
Change
Objectives
Subjective needs
Patients describe when it is necessary to ask for professional/medical help
BACK
What this could look like in practice:
Provide patients with comprehensive educaton on the warning signs and symptoms that may indicate a need for professional or medical help. Develop clear guidelines or a reference document that outlines specific situations when it is necessary to ask for professional or medical help. Highlight the benefits of early intervention and prompt medical attention in managing cardiac conditions. Recognise that patients may have fears or concerns about reaching out for professional or medical help. Utilise visual aids, such as infographics or posters, to illustrate the importance of seeking professional help. Provide personalised reminders to patients about the importance of seeking professional or medical help.
Method used:
Persuasive communication
HOME

## Slide 48
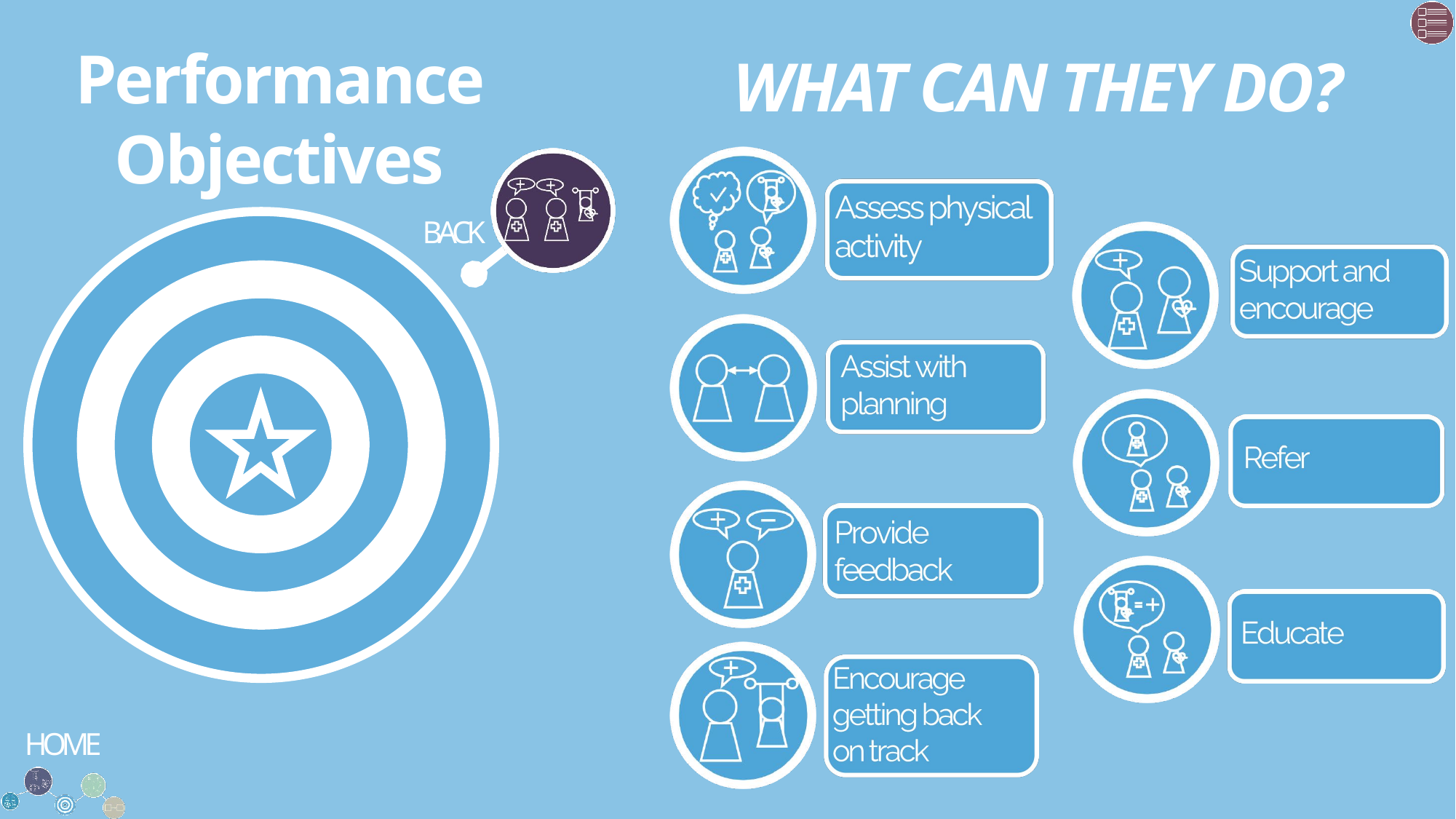

Performance Objectives for healthcare providers
Performance Objectives
WHAT CAN THEY DO?
BACK
HOME

## Slide 49
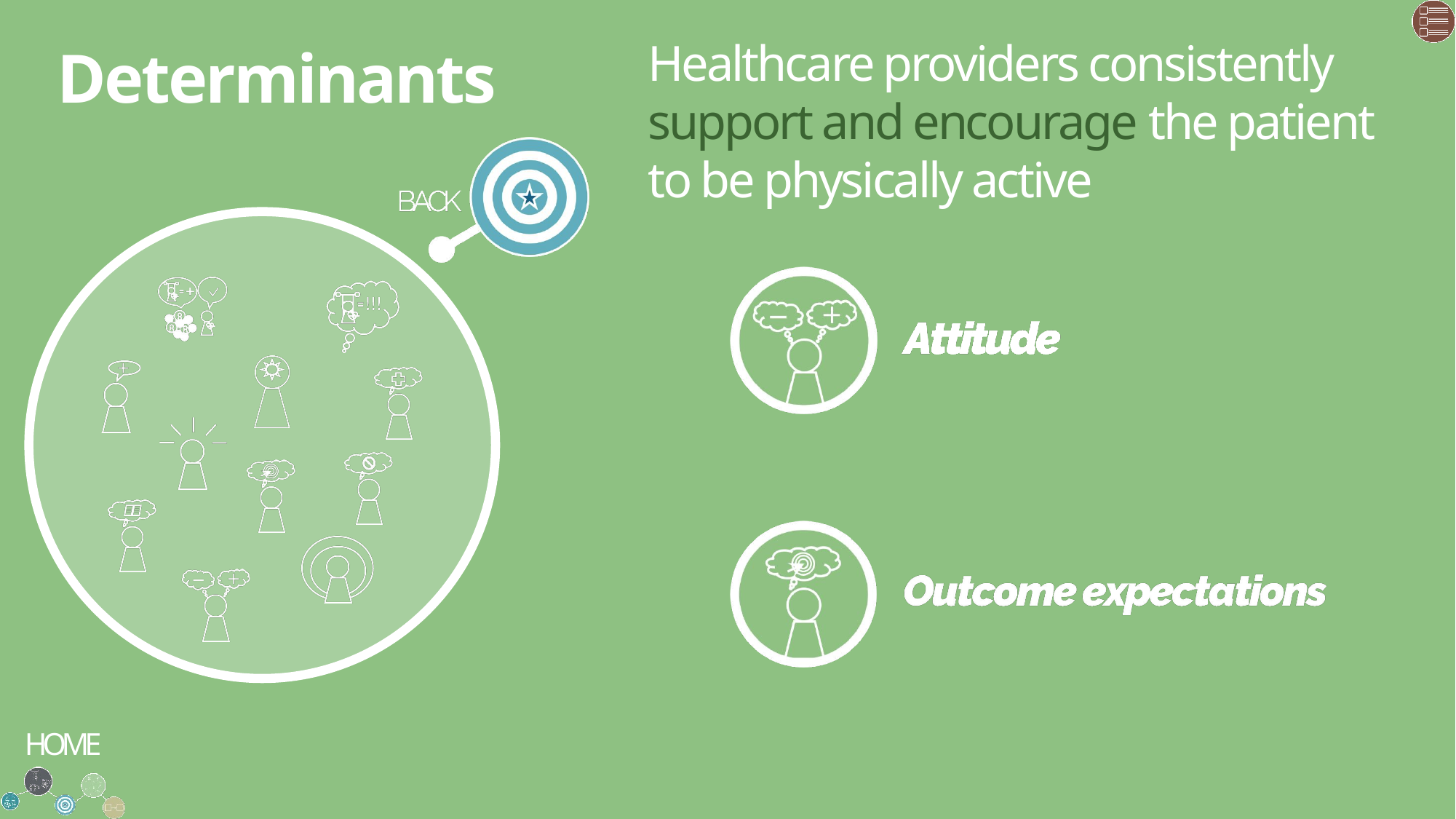

PO1 Determinants for PO1 for healthcare providers
Healthcare providers consistently support and encourage the patient to be physically active
Determinants
HOME

## Slide 50
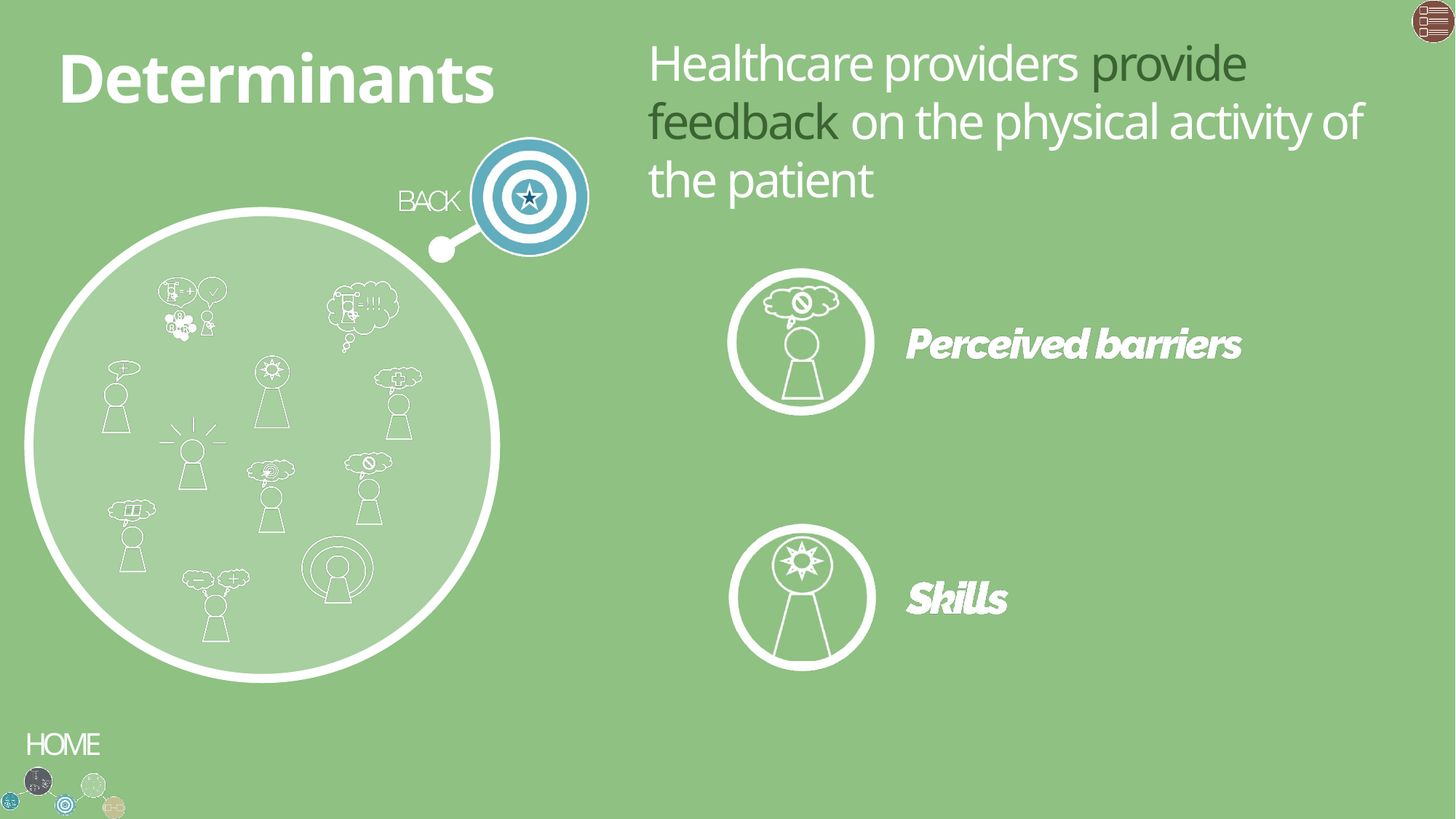

PO2 Determinants for PO2 for healthcare providers
Healthcare providers provide feedback on the physical activity of the patient
Determinants
HOME

## Slide 51
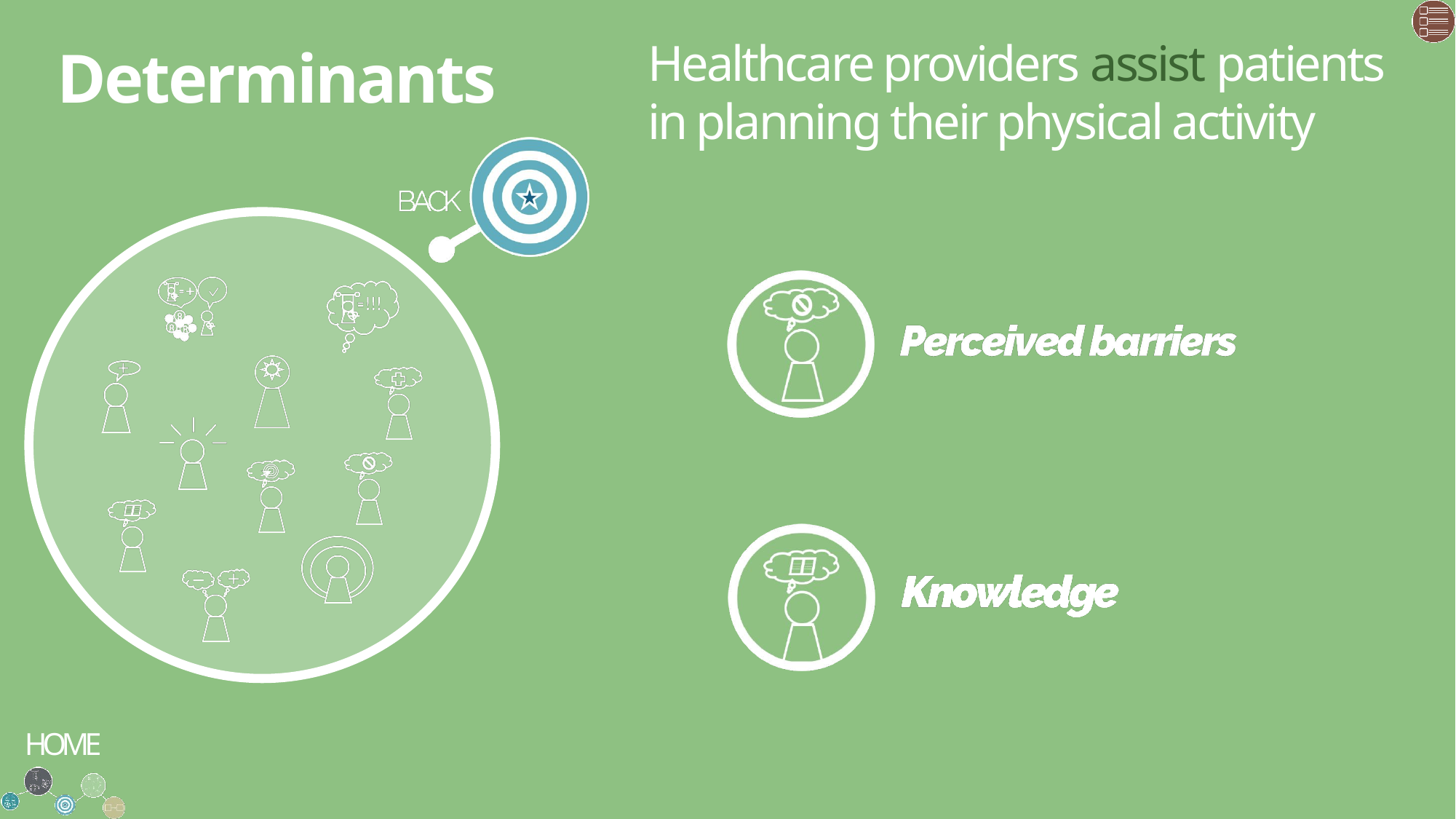

PO3 Determinants for PO3 for healthcare providers
Healthcare providers assist patients in planning their physical activity
Determinants
HOME

## Slide 52
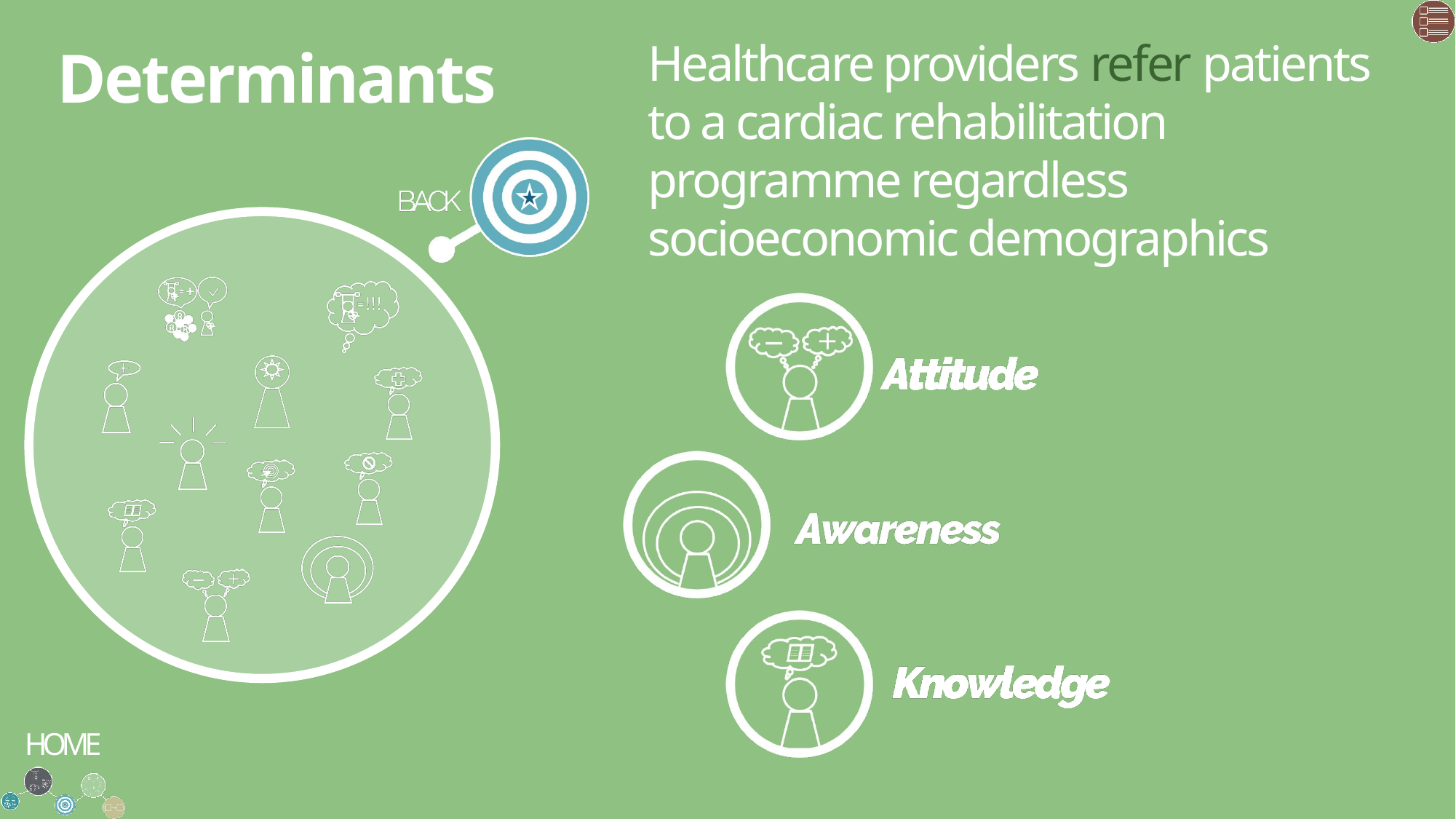

PO4 Determinants for PO4 for healthcare providers
Healthcare providers refer patients to a cardiac rehabilitation programme regardless socioeconomic demographics
Determinants
HOME

## Slide 53
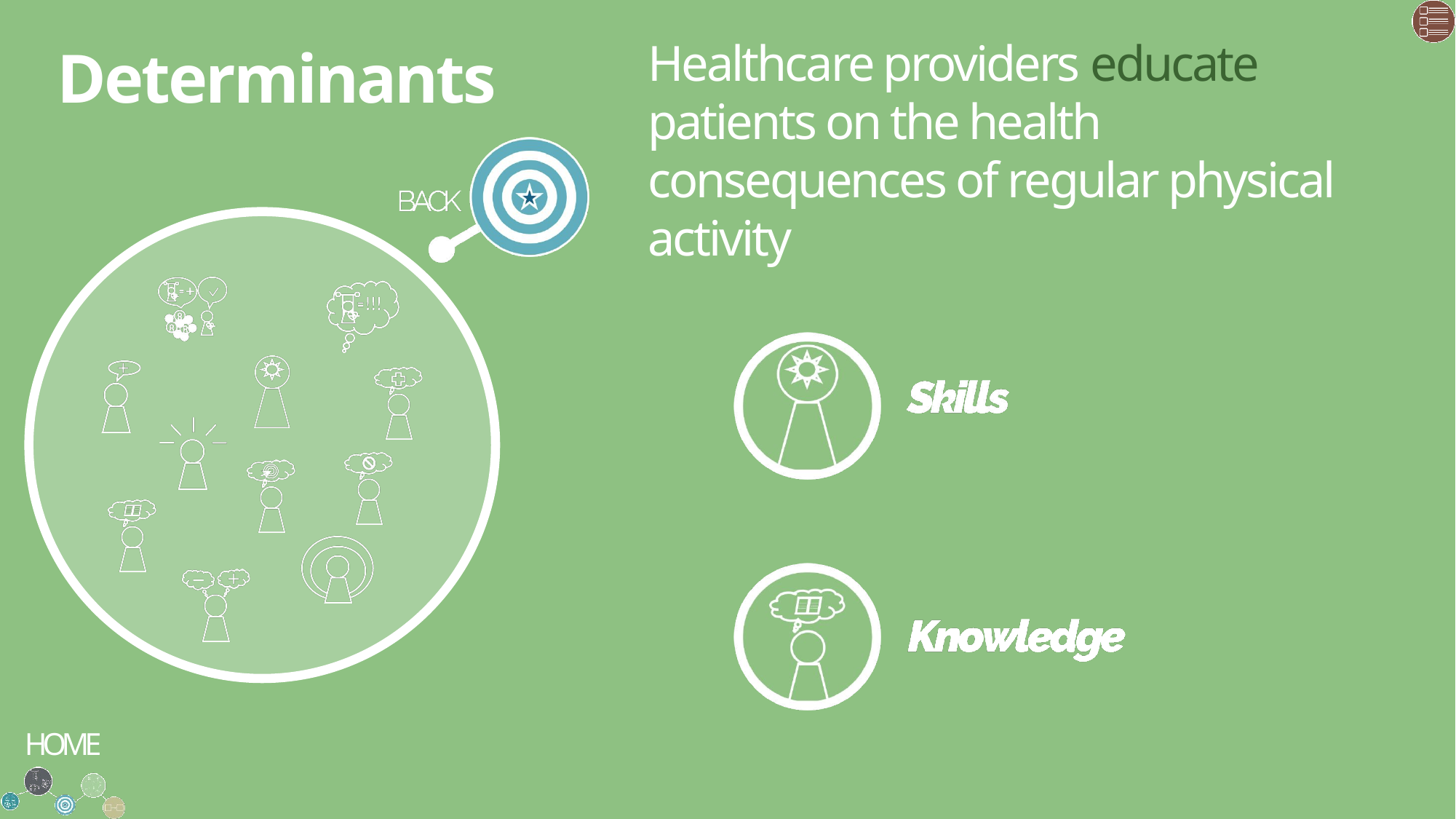

PO5 Determinants for PO5 for healthcare providers
Healthcare providers educate patients on the health consequences of regular physical activity
Determinants
HOME

## Slide 54
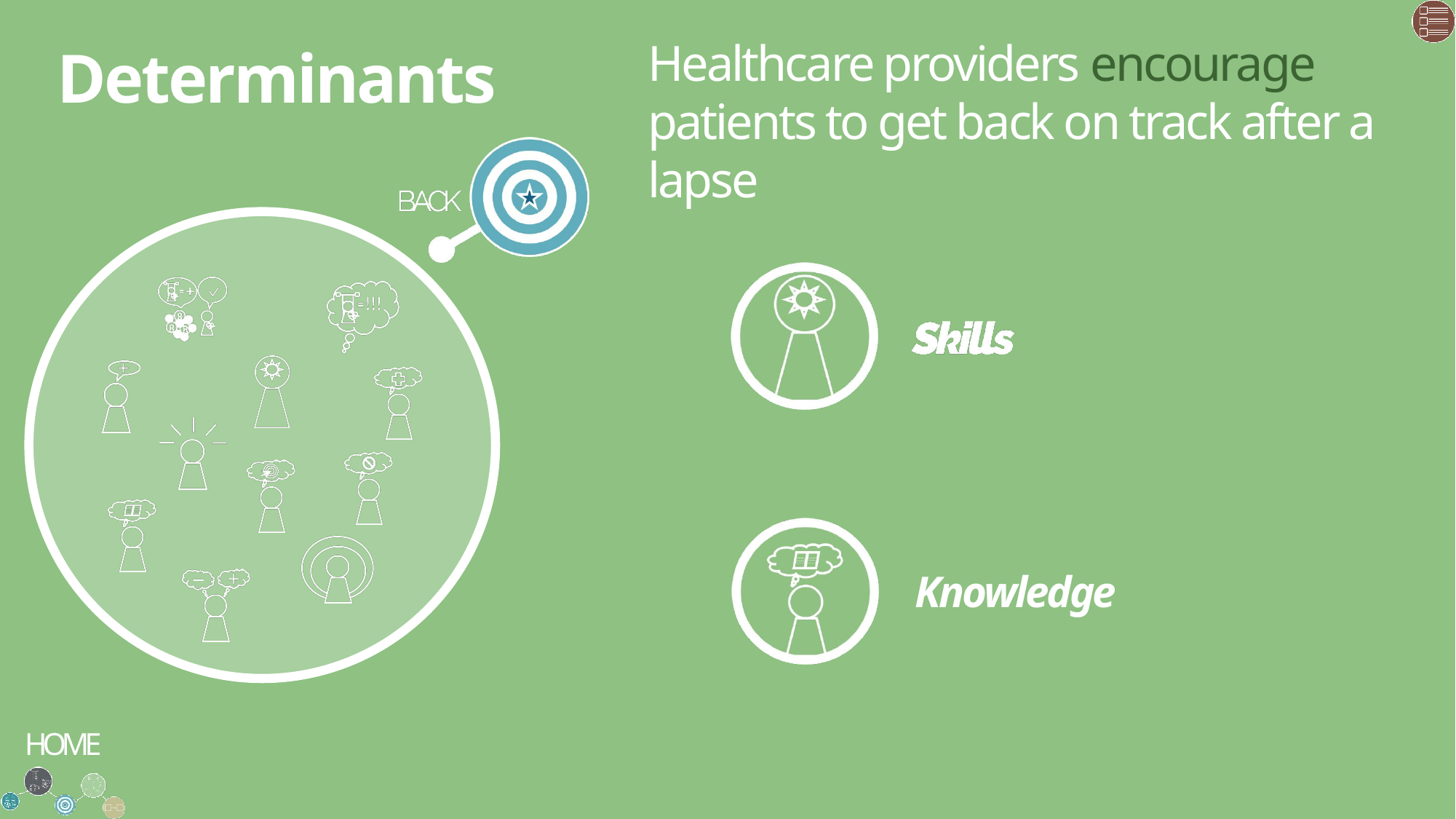

PO6 Determinants for PO6 for healthcare providers
Healthcare providers encourage patients to get back on track after a lapse
Determinants
Knowledge
HOME

## Slide 55
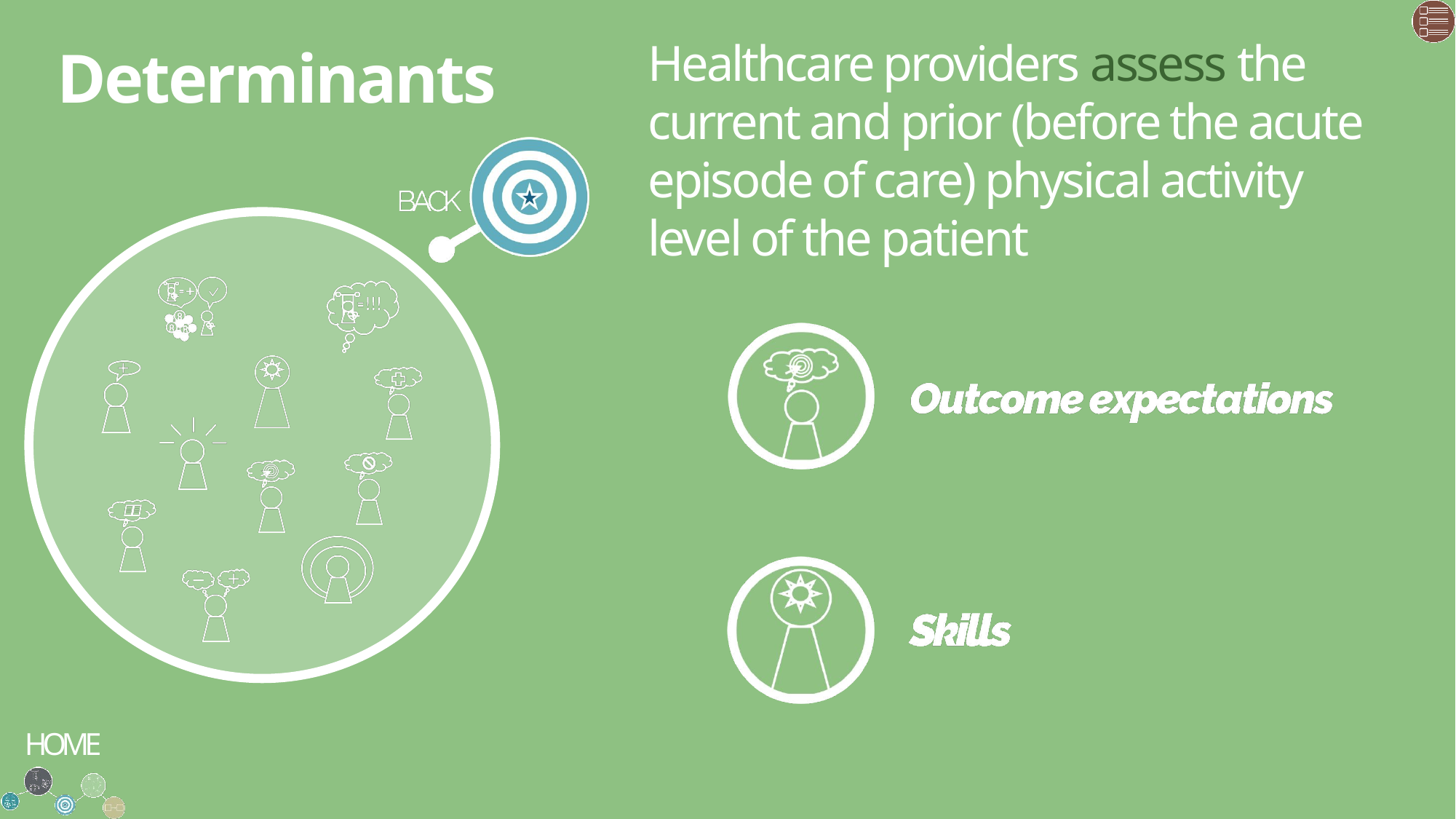

PO7 Determinants for PO7 for healthcare providers
Healthcare providers assess the current and prior (before the acute episode of care) physical activity level of the patient
Determinants
HOME

## Slide 56
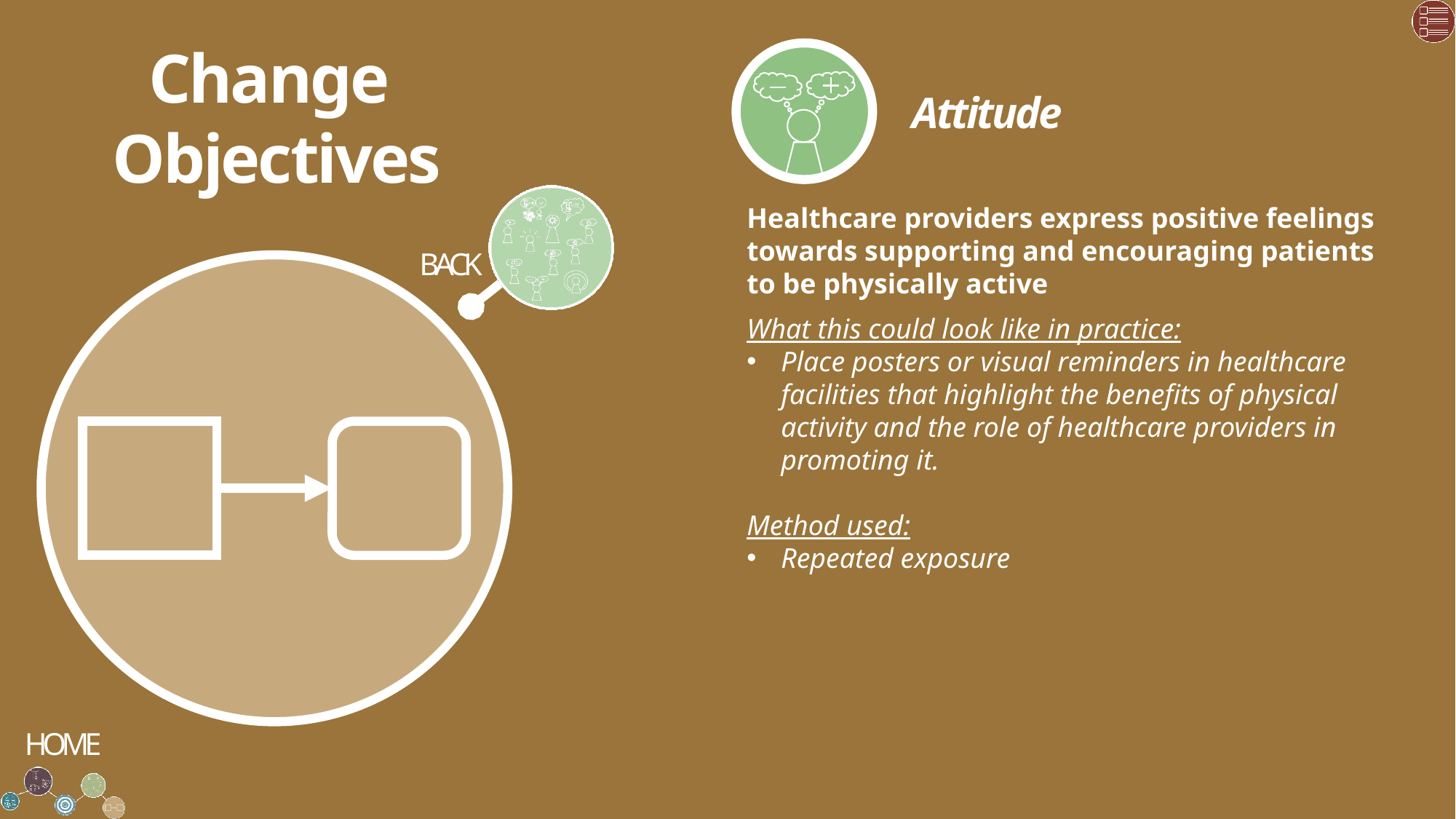

PO1 Att Change objectives for PO1 for healthcare providers
Change
Objectives
Attitude
Healthcare providers express positive feelings towards supporting and encouraging patients to be physically active
BACK
What this could look like in practice:
Place posters or visual reminders in healthcare facilities that highlight the benefits of physical activity and the role of healthcare providers in promoting it.
Method used:
Repeated exposure
HOME

## Slide 57
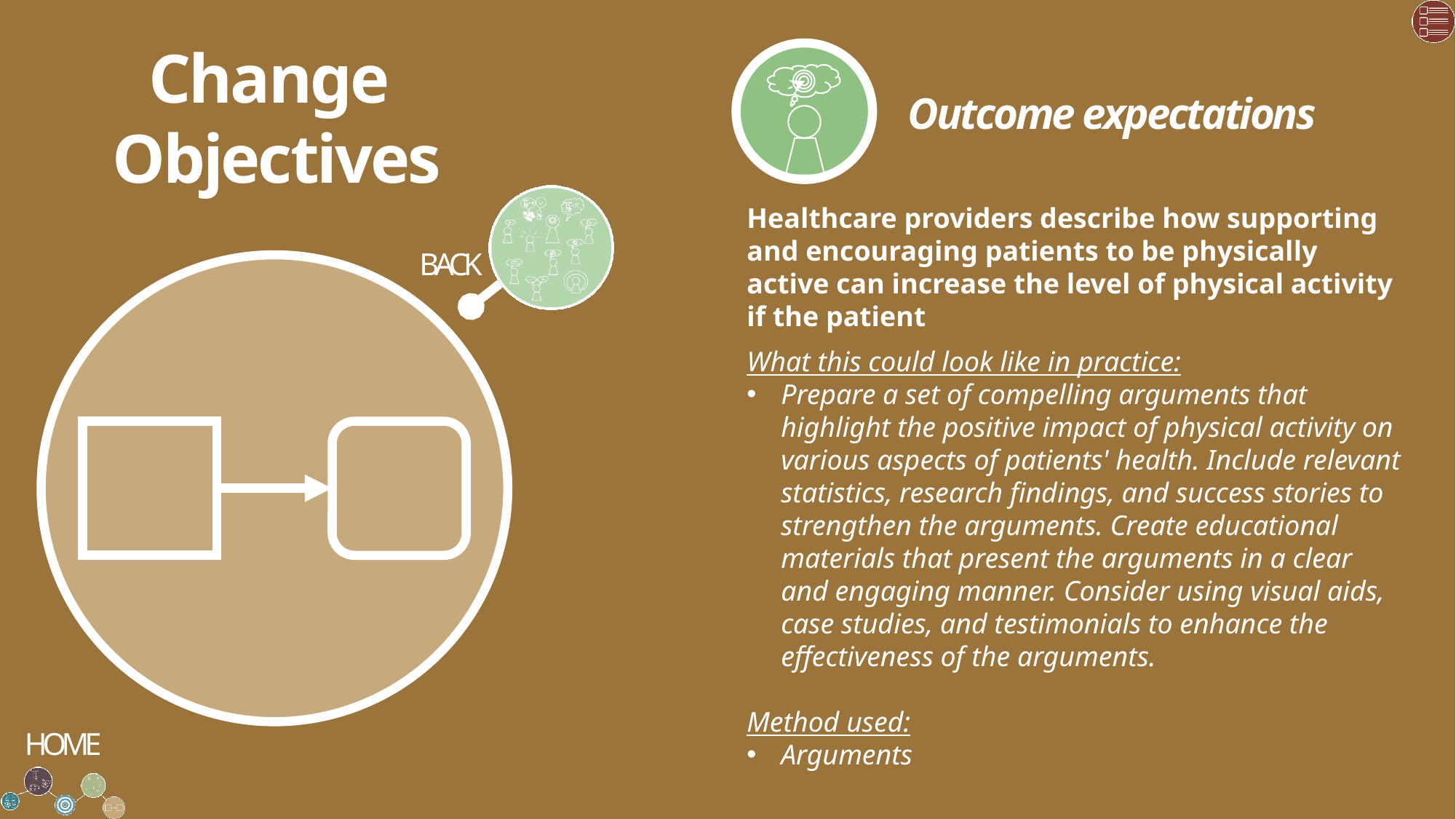

PO1 OE Change objectives for PO1 for healthcare providers
Change
Objectives
Outcome expectations
Healthcare providers describe how supporting and encouraging patients to be physically active can increase the level of physical activity if the patient
BACK
What this could look like in practice:
Prepare a set of compelling arguments that highlight the positive impact of physical activity on various aspects of patients' health. Include relevant statistics, research findings, and success stories to strengthen the arguments. Create educational materials that present the arguments in a clear and engaging manner. Consider using visual aids, case studies, and testimonials to enhance the effectiveness of the arguments.
Method used:
Arguments
HOME

## Slide 58
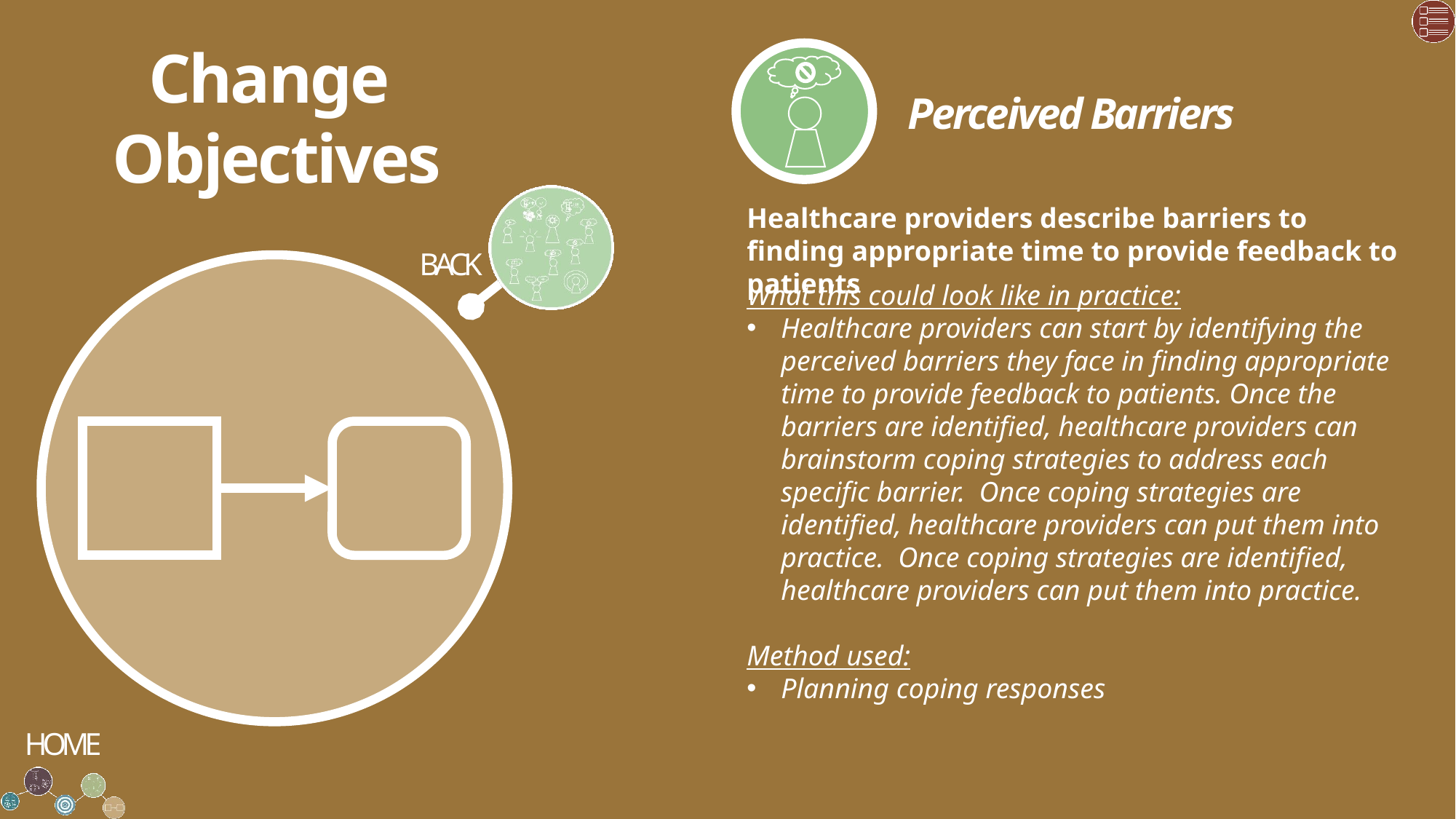

PO2 PB Change objectives for PO2 for healthcare providers
Change
Objectives
Perceived Barriers
Healthcare providers describe barriers to finding appropriate time to provide feedback to patients
BACK
What this could look like in practice:
Healthcare providers can start by identifying the perceived barriers they face in finding appropriate time to provide feedback to patients. Once the barriers are identified, healthcare providers can brainstorm coping strategies to address each specific barrier. Once coping strategies are identified, healthcare providers can put them into practice. Once coping strategies are identified, healthcare providers can put them into practice.
Method used:
Planning coping responses
HOME

## Slide 59
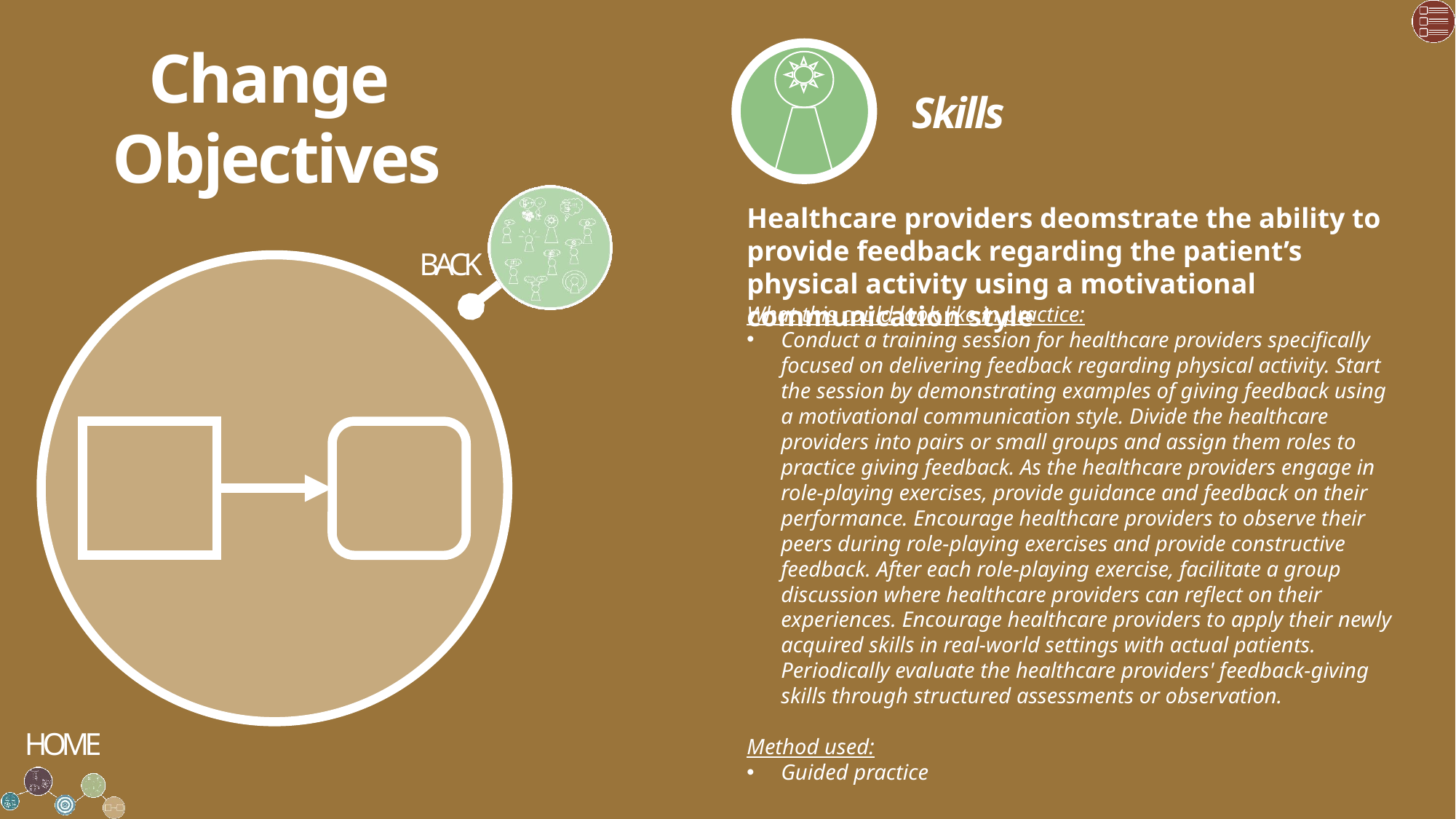

PO2 S Change objectives for PO2 for healthcare providers
Change
Objectives
Skills
Healthcare providers deomstrate the ability to provide feedback regarding the patient’s physical activity using a motivational communication style
BACK
What this could look like in practice:
Conduct a training session for healthcare providers specifically focused on delivering feedback regarding physical activity. Start the session by demonstrating examples of giving feedback using a motivational communication style. Divide the healthcare providers into pairs or small groups and assign them roles to practice giving feedback. As the healthcare providers engage in role-playing exercises, provide guidance and feedback on their performance. Encourage healthcare providers to observe their peers during role-playing exercises and provide constructive feedback. After each role-playing exercise, facilitate a group discussion where healthcare providers can reflect on their experiences. Encourage healthcare providers to apply their newly acquired skills in real-world settings with actual patients. Periodically evaluate the healthcare providers' feedback-giving skills through structured assessments or observation.
Method used:
Guided practice
HOME

## Slide 60
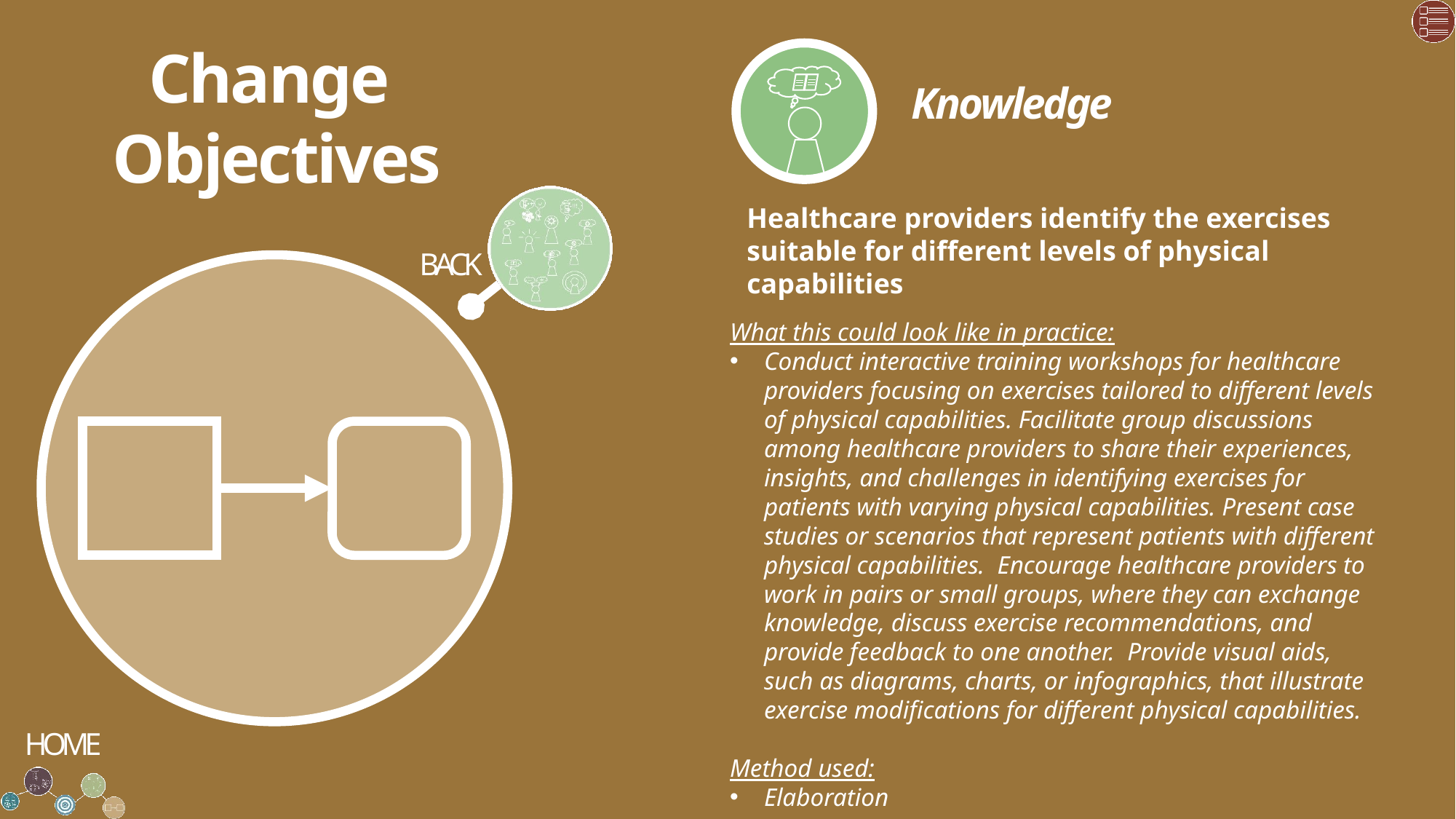

PO3 K Change objectives for PO3 for healthcare providers
Change
Objectives
Knowledge
Healthcare providers identify the exercises suitable for different levels of physical capabilities
BACK
What this could look like in practice:
Conduct interactive training workshops for healthcare providers focusing on exercises tailored to different levels of physical capabilities. Facilitate group discussions among healthcare providers to share their experiences, insights, and challenges in identifying exercises for patients with varying physical capabilities. Present case studies or scenarios that represent patients with different physical capabilities. Encourage healthcare providers to work in pairs or small groups, where they can exchange knowledge, discuss exercise recommendations, and provide feedback to one another. Provide visual aids, such as diagrams, charts, or infographics, that illustrate exercise modifications for different physical capabilities.
Method used:
Elaboration
HOME

## Slide 61
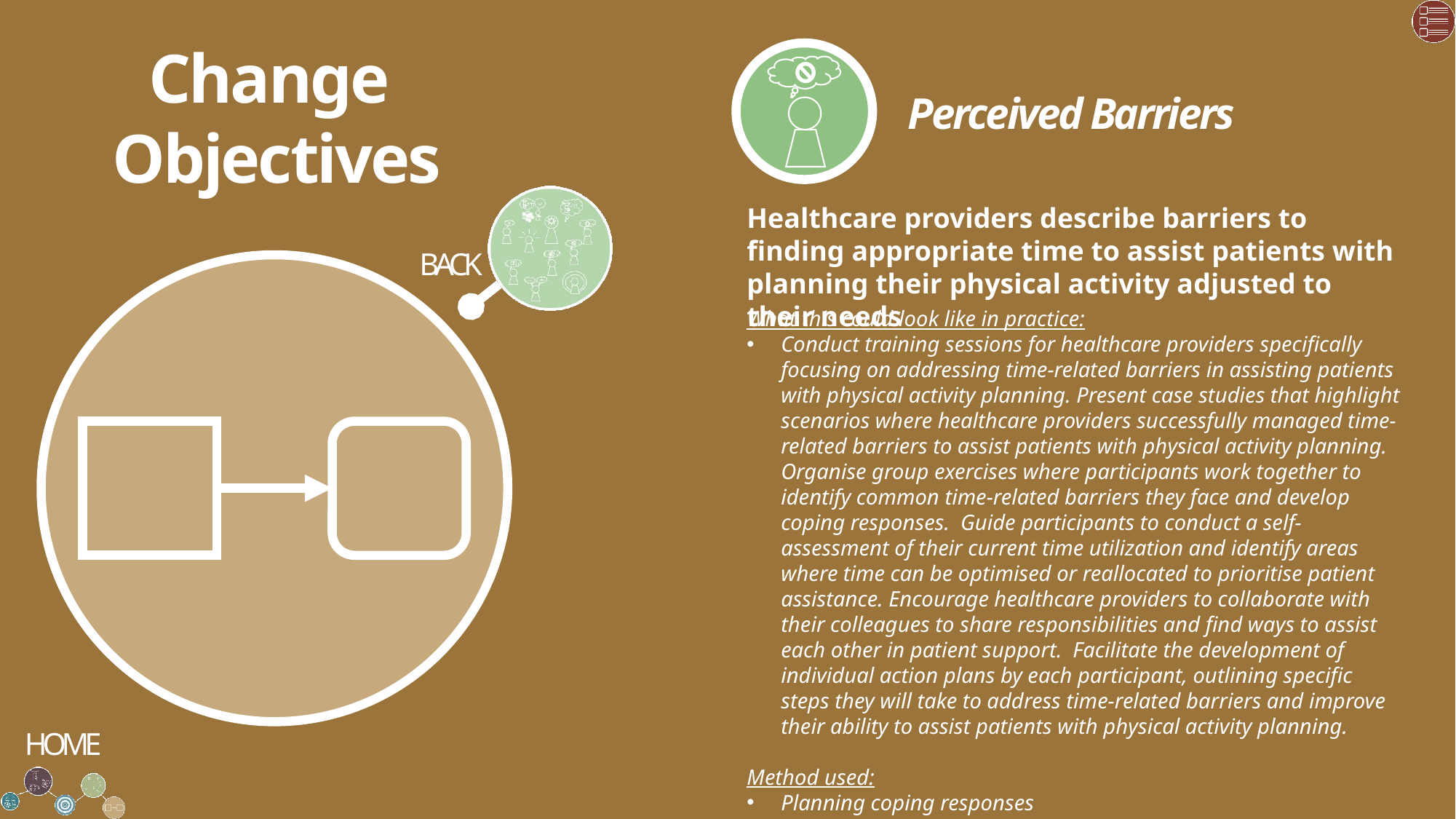

PO3 PB Change objectives for PO3 for healthcare providers
Change
Objectives
Perceived Barriers
Healthcare providers describe barriers to finding appropriate time to assist patients with planning their physical activity adjusted to their needs
BACK
What this could look like in practice:
Conduct training sessions for healthcare providers specifically focusing on addressing time-related barriers in assisting patients with physical activity planning. Present case studies that highlight scenarios where healthcare providers successfully managed time-related barriers to assist patients with physical activity planning. Organise group exercises where participants work together to identify common time-related barriers they face and develop coping responses. Guide participants to conduct a self-assessment of their current time utilization and identify areas where time can be optimised or reallocated to prioritise patient assistance. Encourage healthcare providers to collaborate with their colleagues to share responsibilities and find ways to assist each other in patient support. Facilitate the development of individual action plans by each participant, outlining specific steps they will take to address time-related barriers and improve their ability to assist patients with physical activity planning.
Method used:
Planning coping responses
HOME

## Slide 62
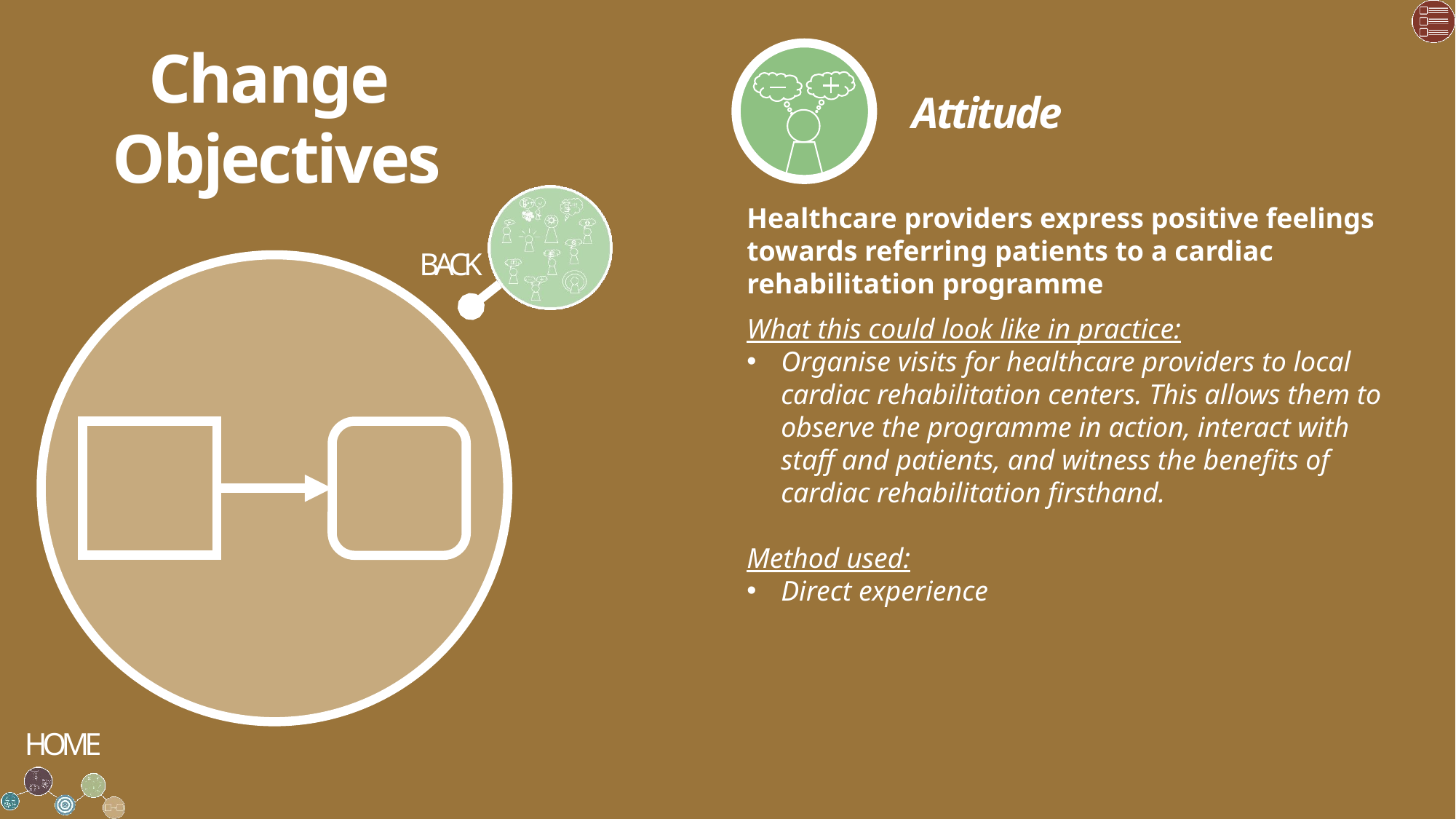

PO4 Att Change objectives for PO4 for healthcare providers
Change
Objectives
Attitude
Healthcare providers express positive feelings towards referring patients to a cardiac rehabilitation programme
BACK
What this could look like in practice:
Organise visits for healthcare providers to local cardiac rehabilitation centers. This allows them to observe the programme in action, interact with staff and patients, and witness the benefits of cardiac rehabilitation firsthand.
Method used:
Direct experience
HOME

## Slide 63
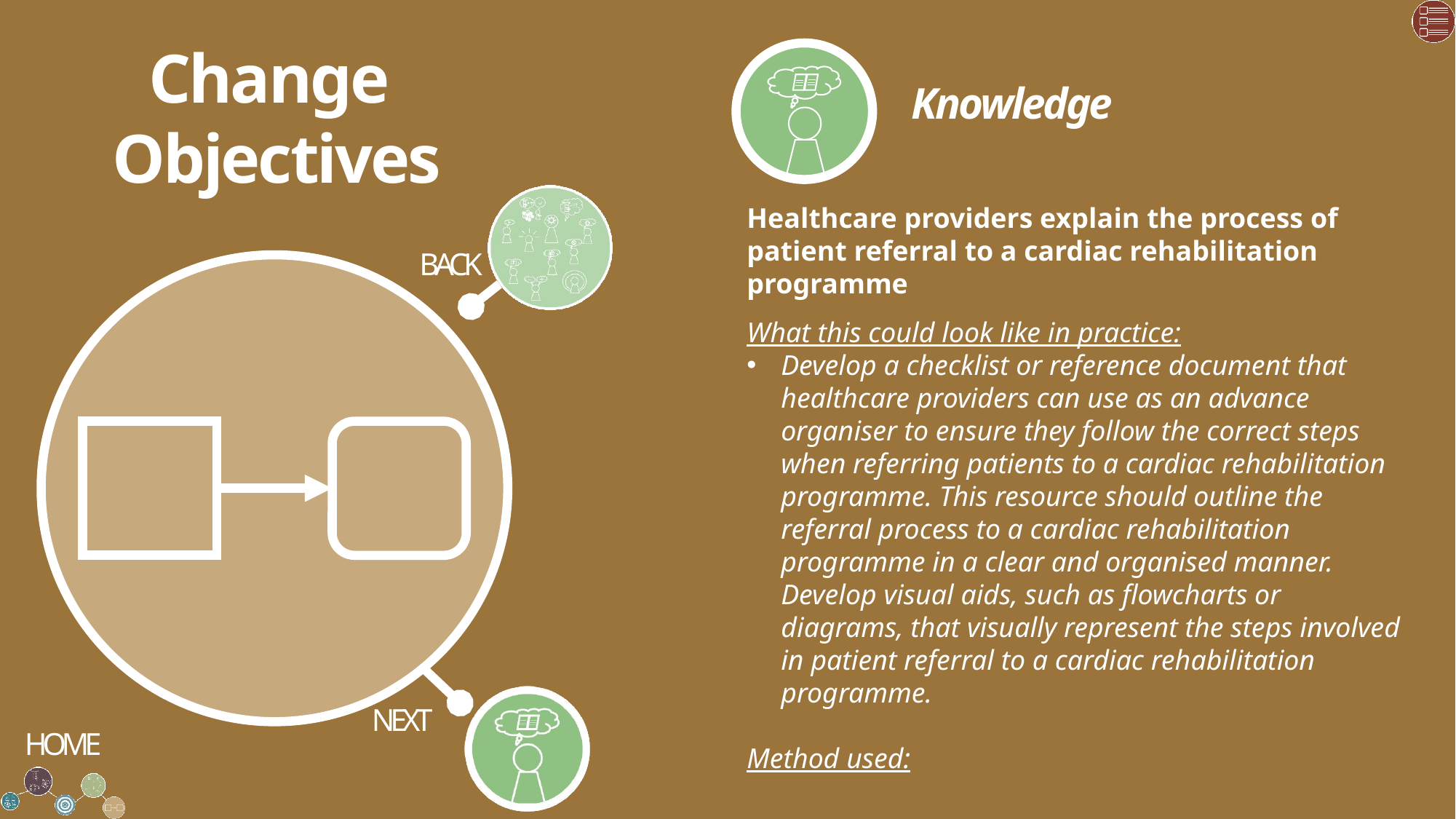

PO4 K Change objectives for PO4 for healthcare providers
Change
Objectives
Knowledge
Healthcare providers explain the process of patient referral to a cardiac rehabilitation programme
BACK
What this could look like in practice:
Develop a checklist or reference document that healthcare providers can use as an advance organiser to ensure they follow the correct steps when referring patients to a cardiac rehabilitation programme. This resource should outline the referral process to a cardiac rehabilitation programme in a clear and organised manner. Develop visual aids, such as flowcharts or diagrams, that visually represent the steps involved in patient referral to a cardiac rehabilitation programme.
Method used:
NEXT
HOME

## Slide 64
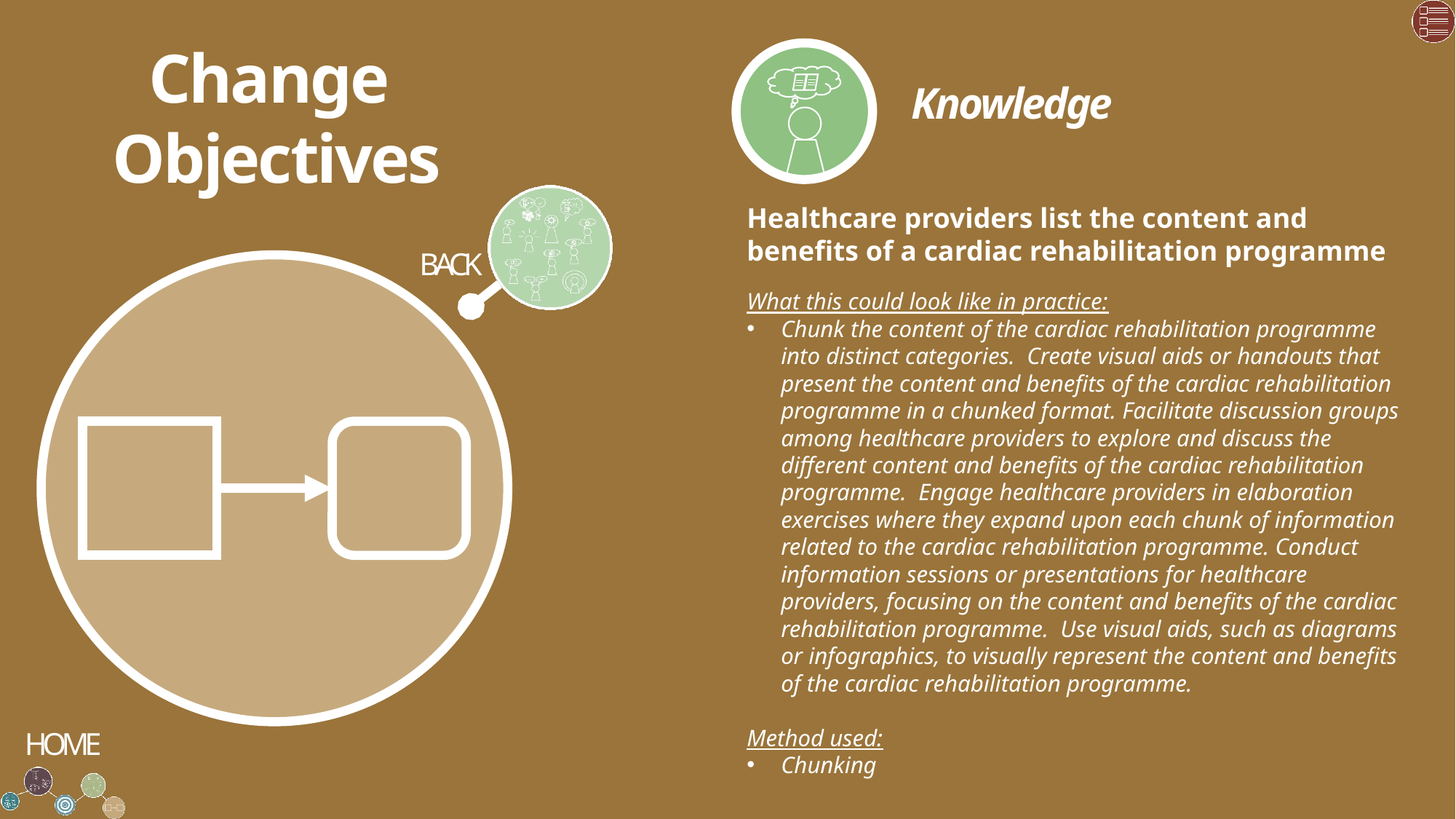

PO4 K Change objectives for PO4 for healthcare providers
Change
Objectives
Knowledge
Healthcare providers list the content and benefits of a cardiac rehabilitation programme
BACK
What this could look like in practice:
Chunk the content of the cardiac rehabilitation programme into distinct categories. Create visual aids or handouts that present the content and benefits of the cardiac rehabilitation programme in a chunked format. Facilitate discussion groups among healthcare providers to explore and discuss the different content and benefits of the cardiac rehabilitation programme. Engage healthcare providers in elaboration exercises where they expand upon each chunk of information related to the cardiac rehabilitation programme. Conduct information sessions or presentations for healthcare providers, focusing on the content and benefits of the cardiac rehabilitation programme. Use visual aids, such as diagrams or infographics, to visually represent the content and benefits of the cardiac rehabilitation programme.
Method used:
Chunking
HOME

## Slide 65
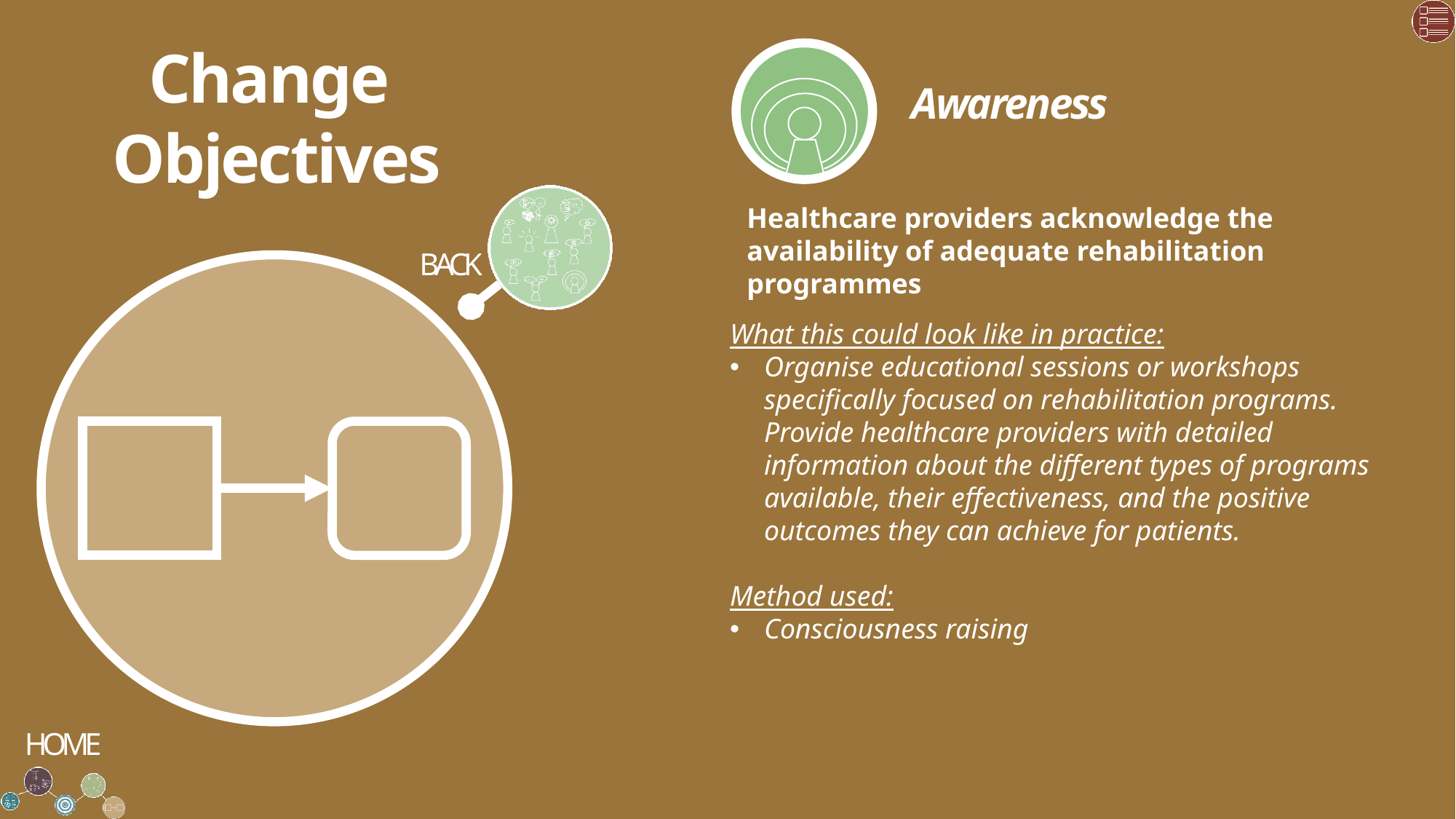

PO4 A Change objectives for PO4 for healthcare providers
Change
Objectives
Awareness
Healthcare providers acknowledge the availability of adequate rehabilitation programmes
BACK
What this could look like in practice:
Organise educational sessions or workshops specifically focused on rehabilitation programs. Provide healthcare providers with detailed information about the different types of programs available, their effectiveness, and the positive outcomes they can achieve for patients.
Method used:
Consciousness raising
HOME

## Slide 66
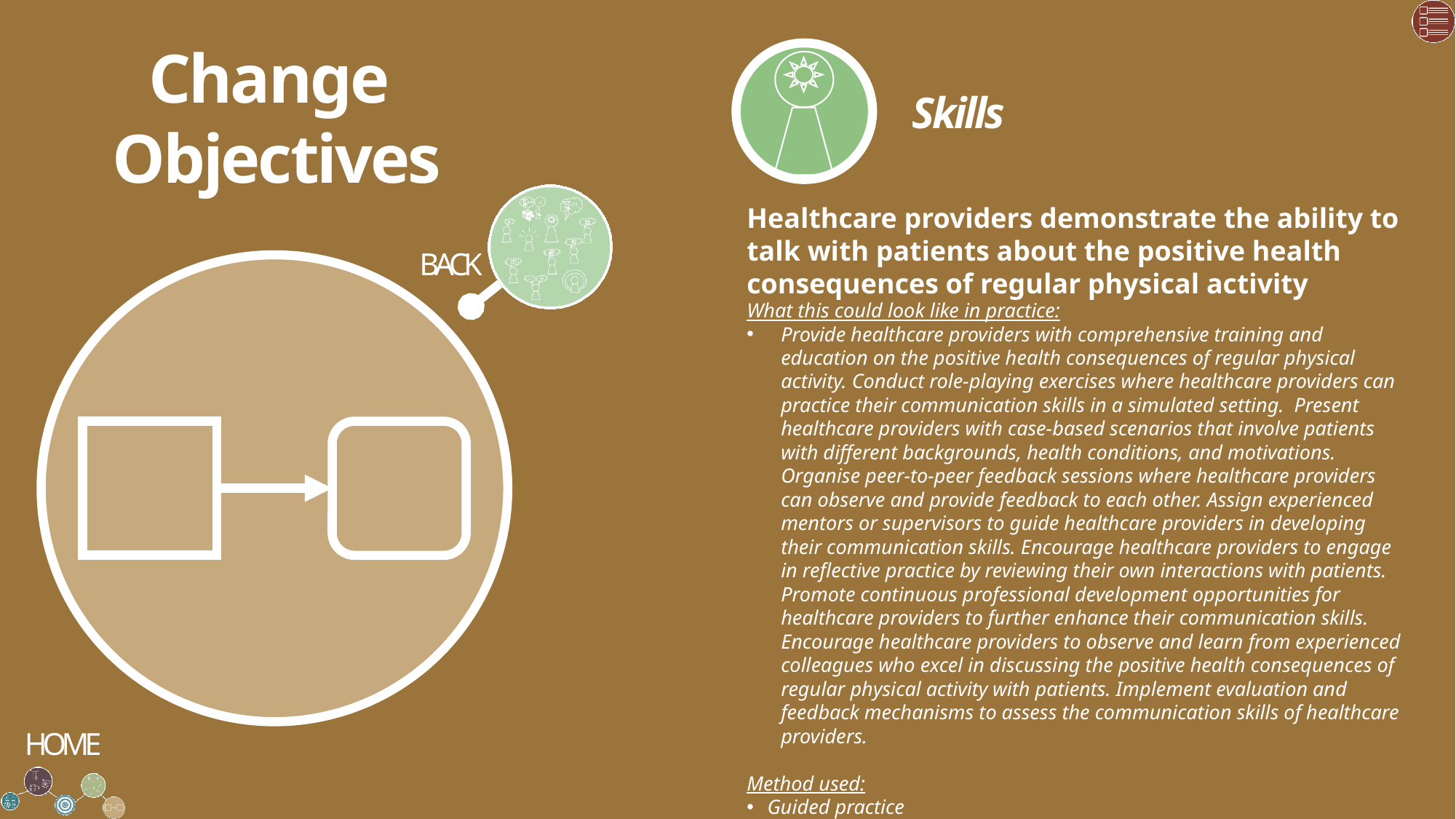

PO5 S Change objectives for PO5 for healthcare providers
Change
Objectives
Skills
Healthcare providers demonstrate the ability to talk with patients about the positive health consequences of regular physical activity
BACK
What this could look like in practice:
Provide healthcare providers with comprehensive training and education on the positive health consequences of regular physical activity. Conduct role-playing exercises where healthcare providers can practice their communication skills in a simulated setting. Present healthcare providers with case-based scenarios that involve patients with different backgrounds, health conditions, and motivations. Organise peer-to-peer feedback sessions where healthcare providers can observe and provide feedback to each other. Assign experienced mentors or supervisors to guide healthcare providers in developing their communication skills. Encourage healthcare providers to engage in reflective practice by reviewing their own interactions with patients. Promote continuous professional development opportunities for healthcare providers to further enhance their communication skills. Encourage healthcare providers to observe and learn from experienced colleagues who excel in discussing the positive health consequences of regular physical activity with patients. Implement evaluation and feedback mechanisms to assess the communication skills of healthcare providers.
Method used:
Guided practice
HOME

## Slide 67
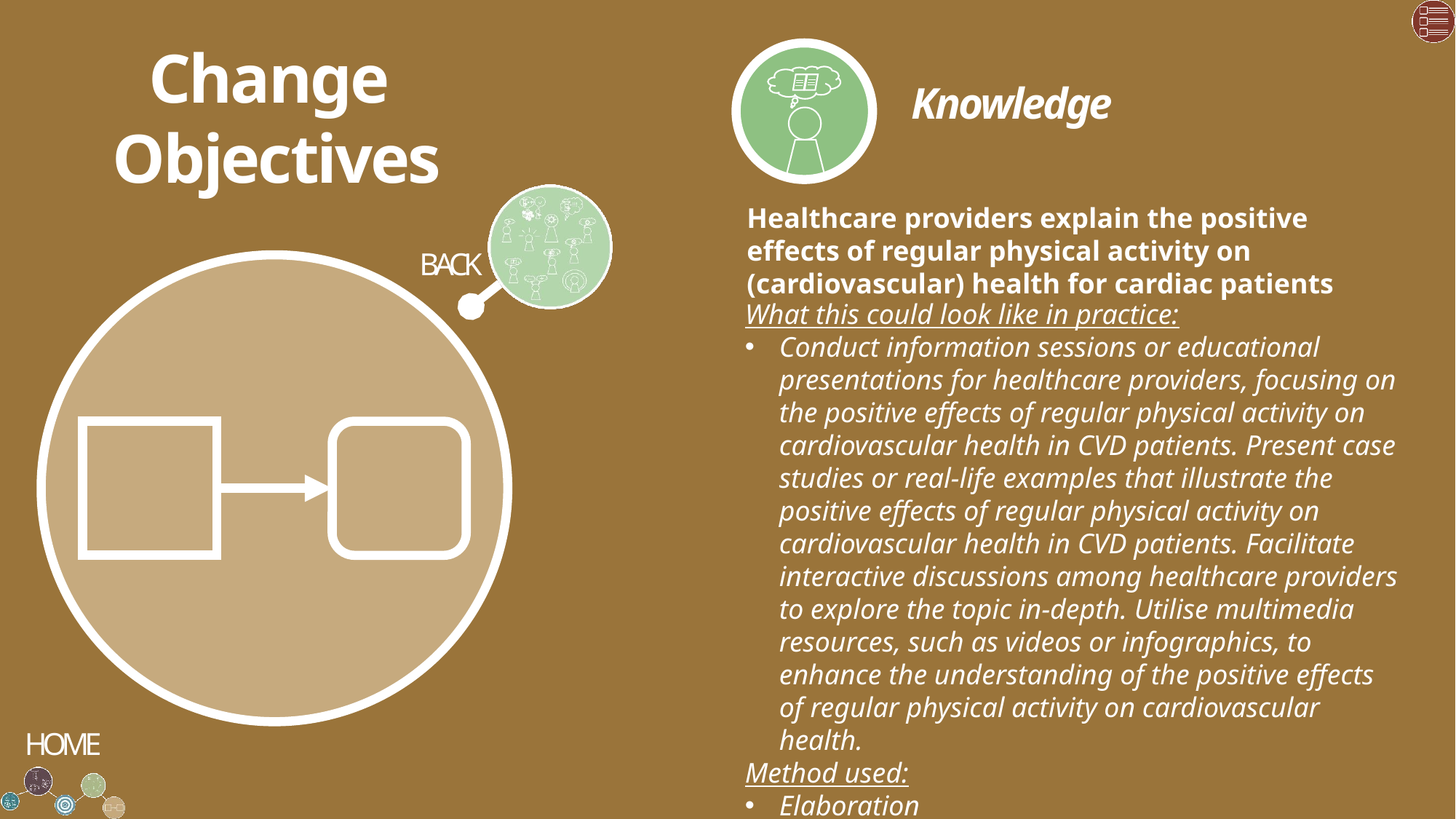

PO5 K Change objectives for PO5 for healthcare providers
Change
Objectives
Knowledge
Healthcare providers explain the positive effects of regular physical activity on (cardiovascular) health for cardiac patients
BACK
What this could look like in practice:
Conduct information sessions or educational presentations for healthcare providers, focusing on the positive effects of regular physical activity on cardiovascular health in CVD patients. Present case studies or real-life examples that illustrate the positive effects of regular physical activity on cardiovascular health in CVD patients. Facilitate interactive discussions among healthcare providers to explore the topic in-depth. Utilise multimedia resources, such as videos or infographics, to enhance the understanding of the positive effects of regular physical activity on cardiovascular health.
Method used:
Elaboration
HOME

## Slide 68
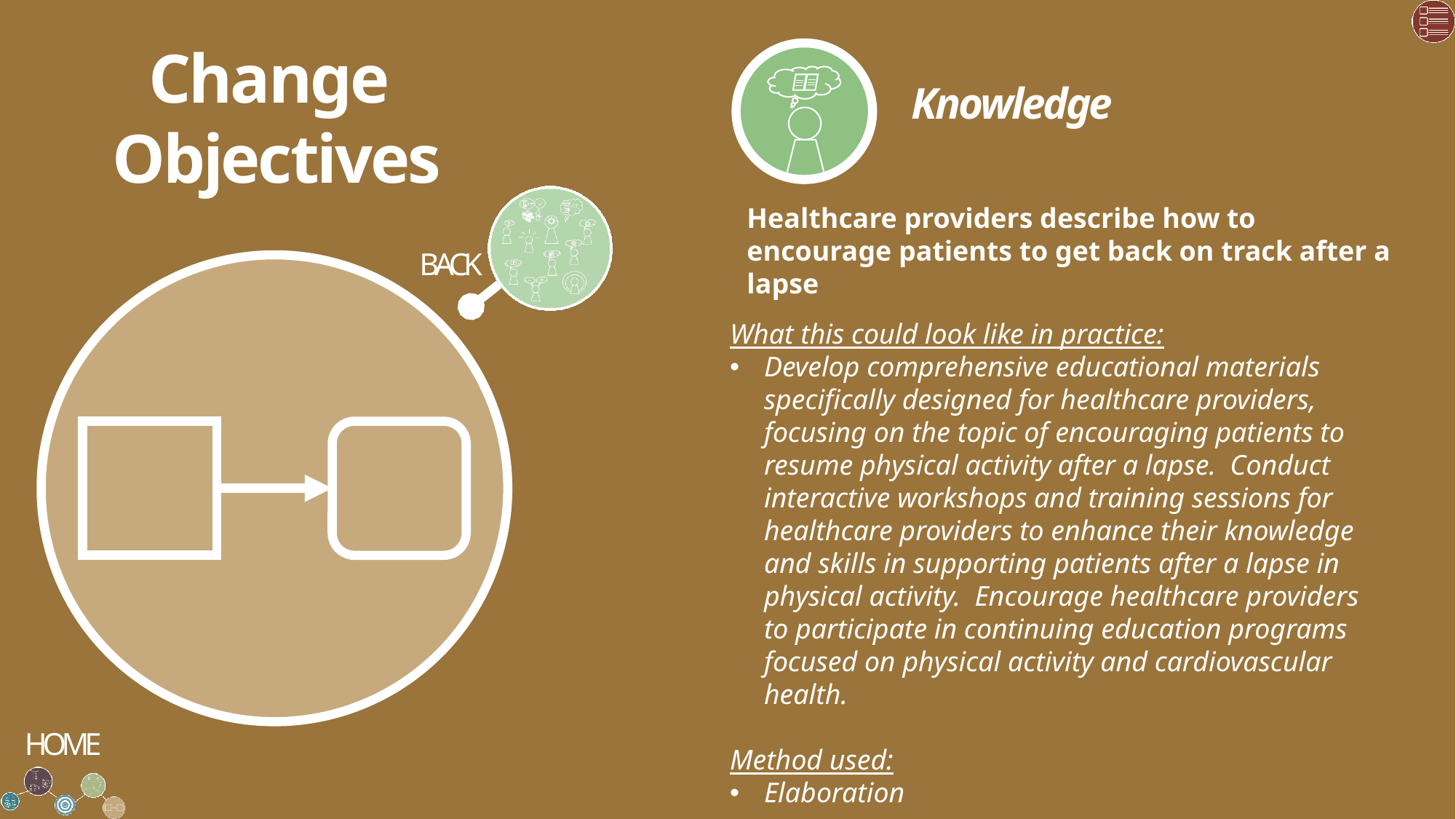

PO6 K Change objectives for PO6 for healthcare providers
Change
Objectives
Knowledge
Healthcare providers describe how to encourage patients to get back on track after a lapse
BACK
What this could look like in practice:
Develop comprehensive educational materials specifically designed for healthcare providers, focusing on the topic of encouraging patients to resume physical activity after a lapse. Conduct interactive workshops and training sessions for healthcare providers to enhance their knowledge and skills in supporting patients after a lapse in physical activity. Encourage healthcare providers to participate in continuing education programs focused on physical activity and cardiovascular health.
Method used:
Elaboration
HOME

## Slide 69
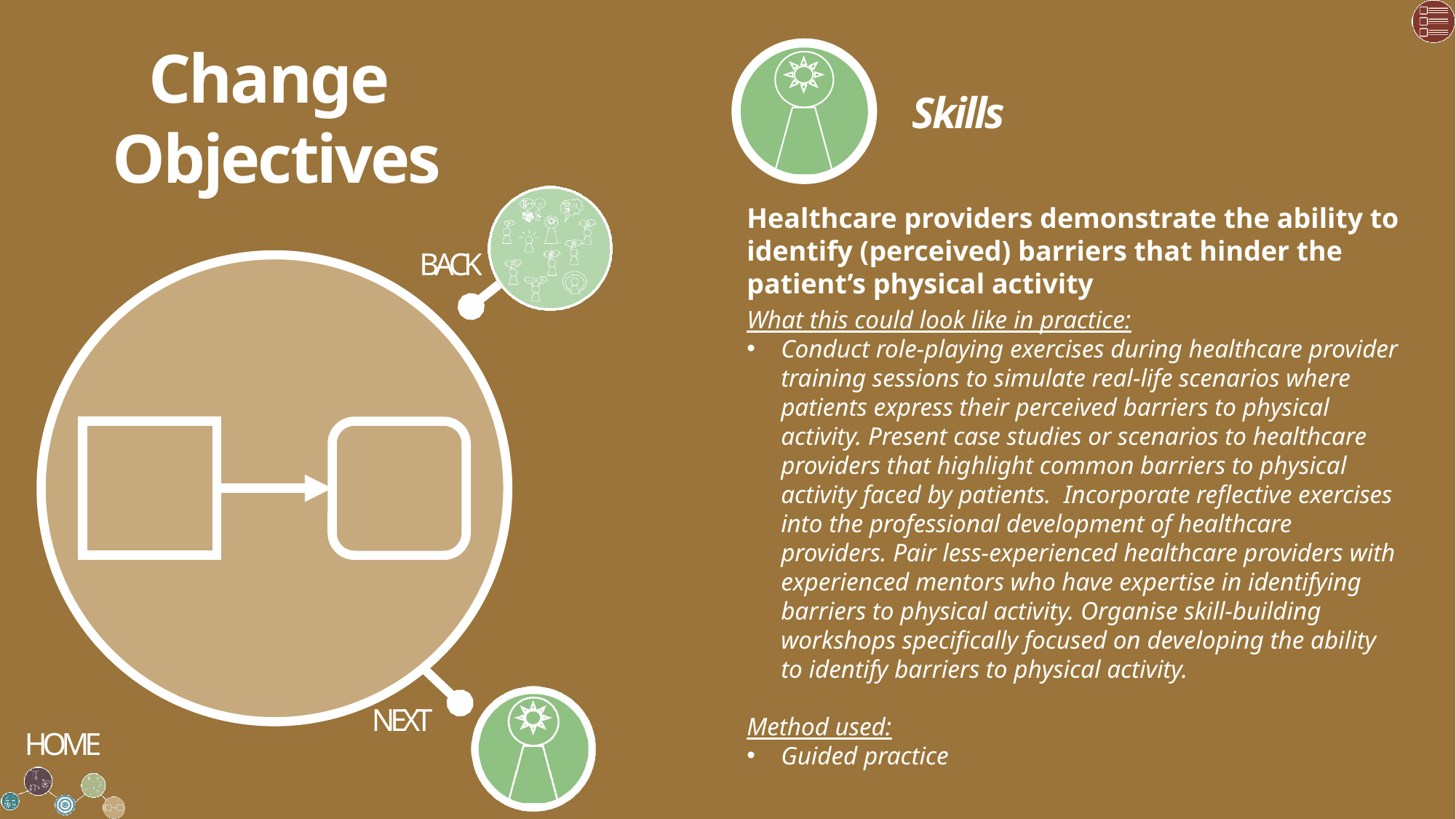

PO6 S Change objectives for PO6 for healthcare providers
Change
Objectives
Skills
Healthcare providers demonstrate the ability to identify (perceived) barriers that hinder the patient’s physical activity
BACK
What this could look like in practice:
Conduct role-playing exercises during healthcare provider training sessions to simulate real-life scenarios where patients express their perceived barriers to physical activity. Present case studies or scenarios to healthcare providers that highlight common barriers to physical activity faced by patients. Incorporate reflective exercises into the professional development of healthcare providers. Pair less-experienced healthcare providers with experienced mentors who have expertise in identifying barriers to physical activity. Organise skill-building workshops specifically focused on developing the ability to identify barriers to physical activity.
Method used:
Guided practice
NEXT
HOME

## Slide 70
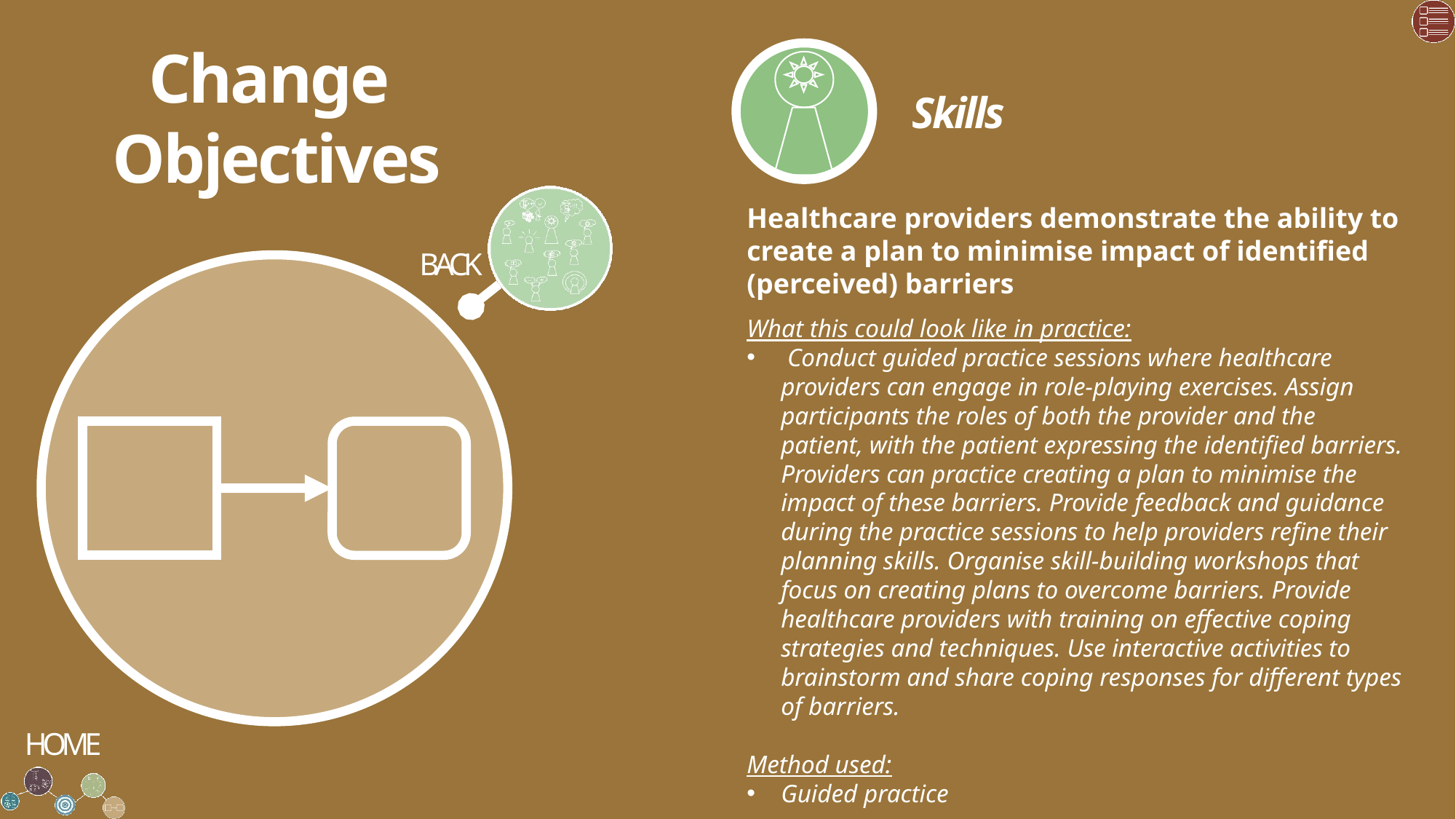

PO6 S Change objectives for PO6 for healthcare providers
Change
Objectives
Skills
Healthcare providers demonstrate the ability to create a plan to minimise impact of identified (perceived) barriers
BACK
What this could look like in practice:
 Conduct guided practice sessions where healthcare providers can engage in role-playing exercises. Assign participants the roles of both the provider and the patient, with the patient expressing the identified barriers. Providers can practice creating a plan to minimise the impact of these barriers. Provide feedback and guidance during the practice sessions to help providers refine their planning skills. Organise skill-building workshops that focus on creating plans to overcome barriers. Provide healthcare providers with training on effective coping strategies and techniques. Use interactive activities to brainstorm and share coping responses for different types of barriers.
Method used:
Guided practice
HOME

## Slide 71
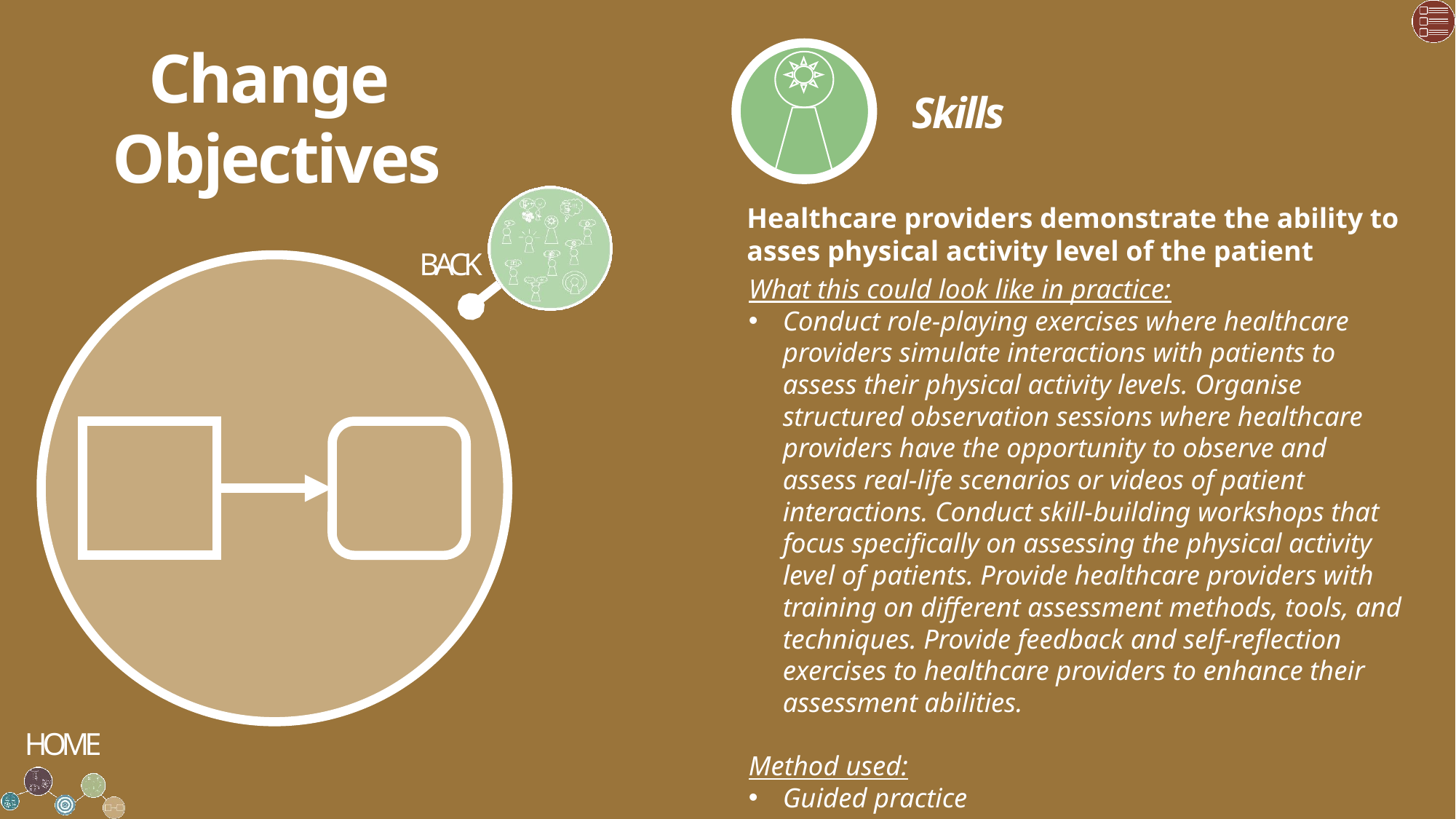

PO7 S Change objectives for healthcare providers
Change
Objectives
Skills
Healthcare providers demonstrate the ability to asses physical activity level of the patient
BACK
What this could look like in practice:
Conduct role-playing exercises where healthcare providers simulate interactions with patients to assess their physical activity levels. Organise structured observation sessions where healthcare providers have the opportunity to observe and assess real-life scenarios or videos of patient interactions. Conduct skill-building workshops that focus specifically on assessing the physical activity level of patients. Provide healthcare providers with training on different assessment methods, tools, and techniques. Provide feedback and self-reflection exercises to healthcare providers to enhance their assessment abilities.
Method used:
Guided practice
HOME

## Slide 72
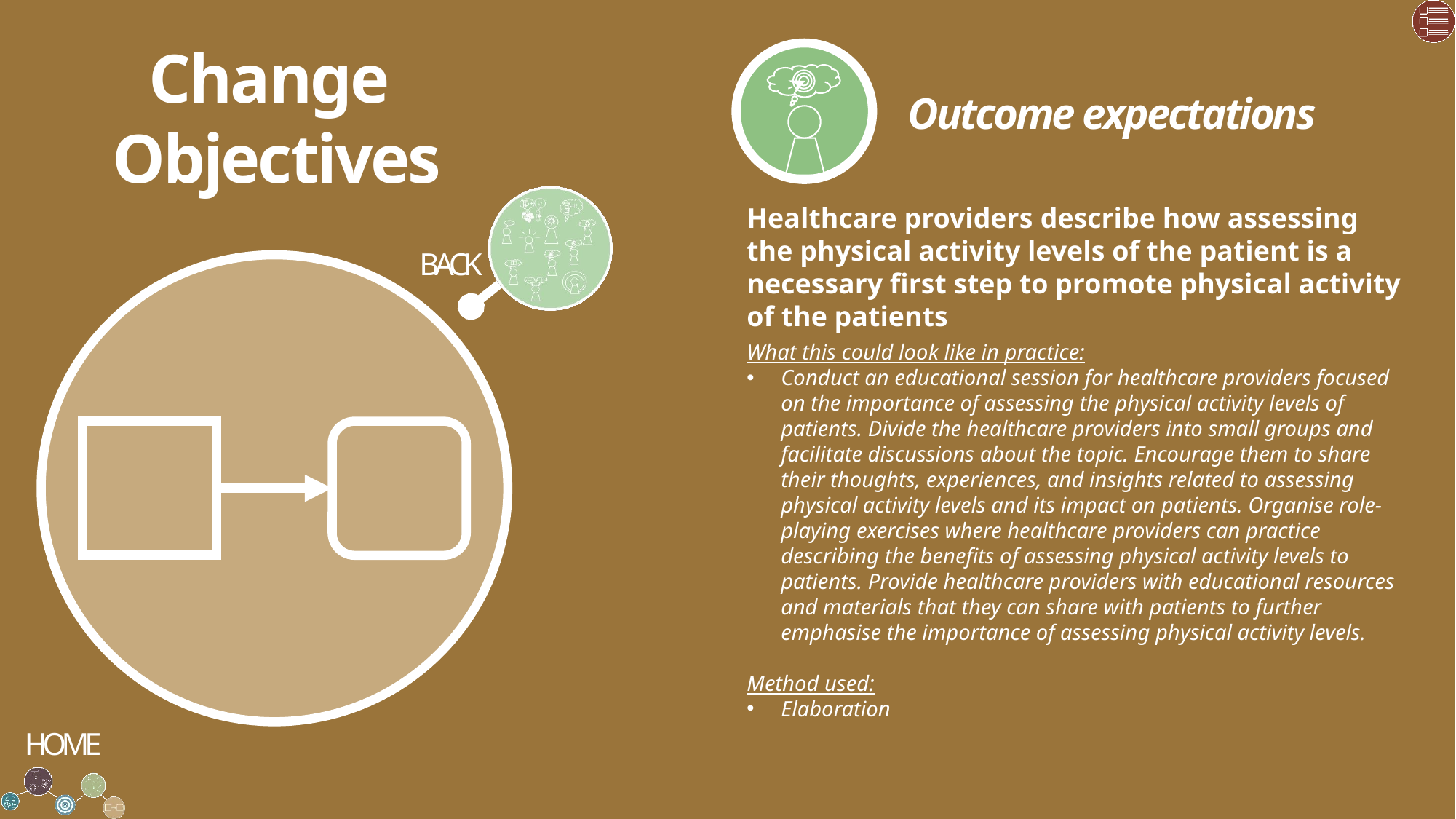

PO7 OE Change objectives for healthcare providers
Change
Objectives
Outcome expectations
Healthcare providers describe how assessing the physical activity levels of the patient is a necessary first step to promote physical activity of the patients
BACK
What this could look like in practice:
Conduct an educational session for healthcare providers focused on the importance of assessing the physical activity levels of patients. Divide the healthcare providers into small groups and facilitate discussions about the topic. Encourage them to share their thoughts, experiences, and insights related to assessing physical activity levels and its impact on patients. Organise role-playing exercises where healthcare providers can practice describing the benefits of assessing physical activity levels to patients. Provide healthcare providers with educational resources and materials that they can share with patients to further emphasise the importance of assessing physical activity levels.
Method used:
Elaboration
HOME

## Slide 73
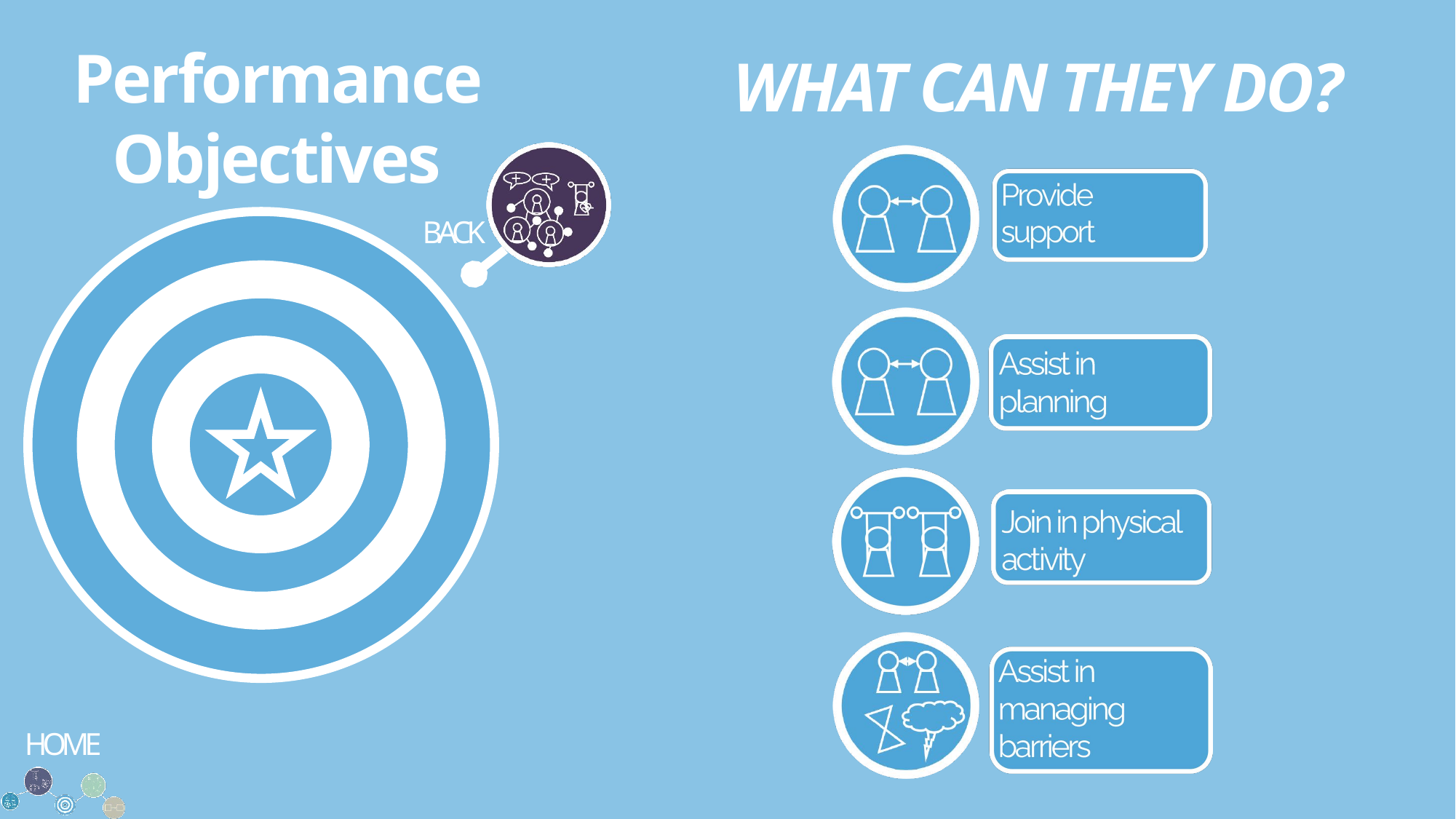

Performance Objectives for people in the patient’s interpersonal environment
Performance Objectives
WHAT CAN THEY DO?
BACK
HOME

## Slide 74
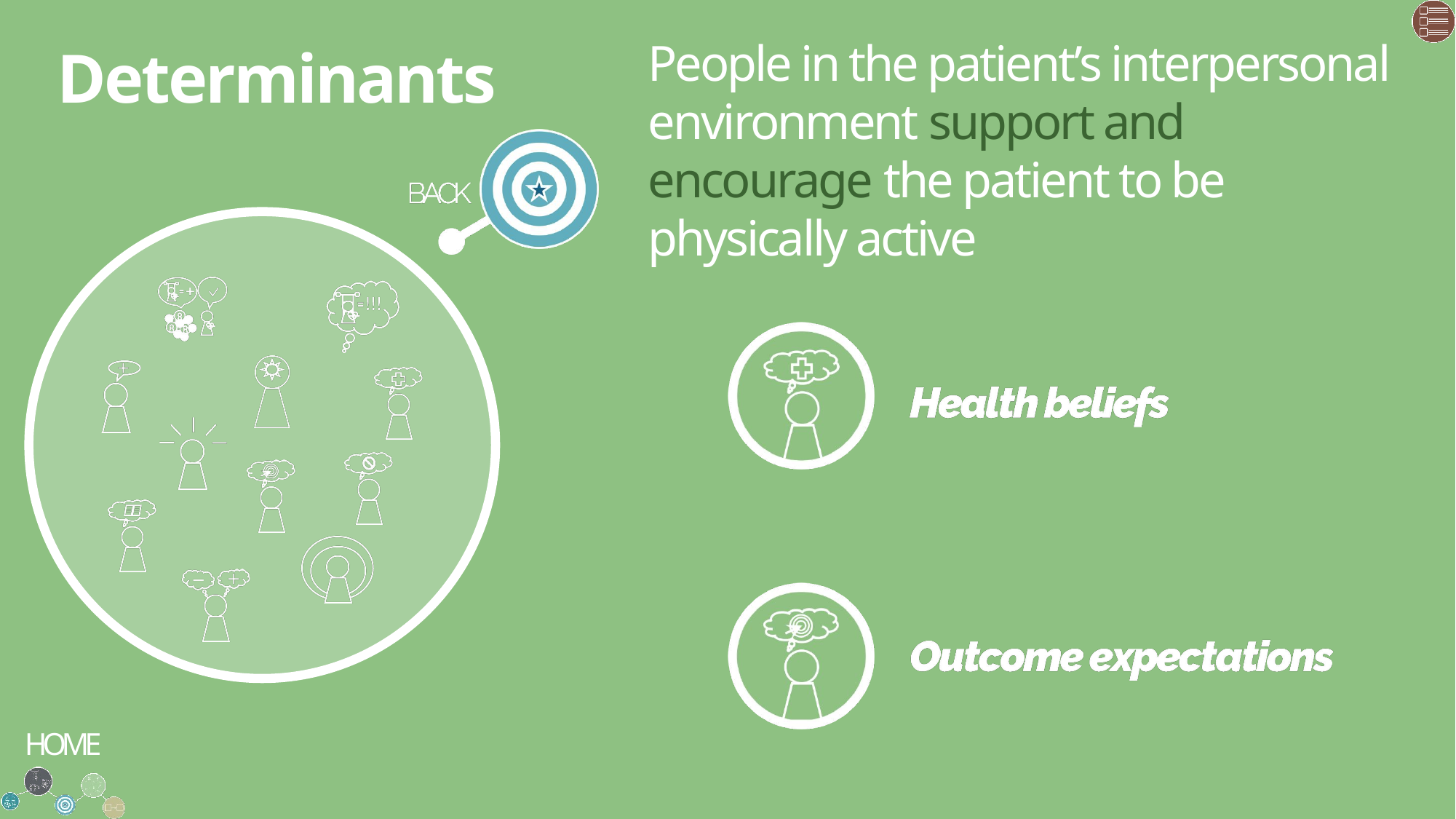

PO1 Determinants for PO1 for people in the patient’s interpersonal environment
People in the patient’s interpersonal environment support and encourage the patient to be physically active
Determinants
HOME

## Slide 75
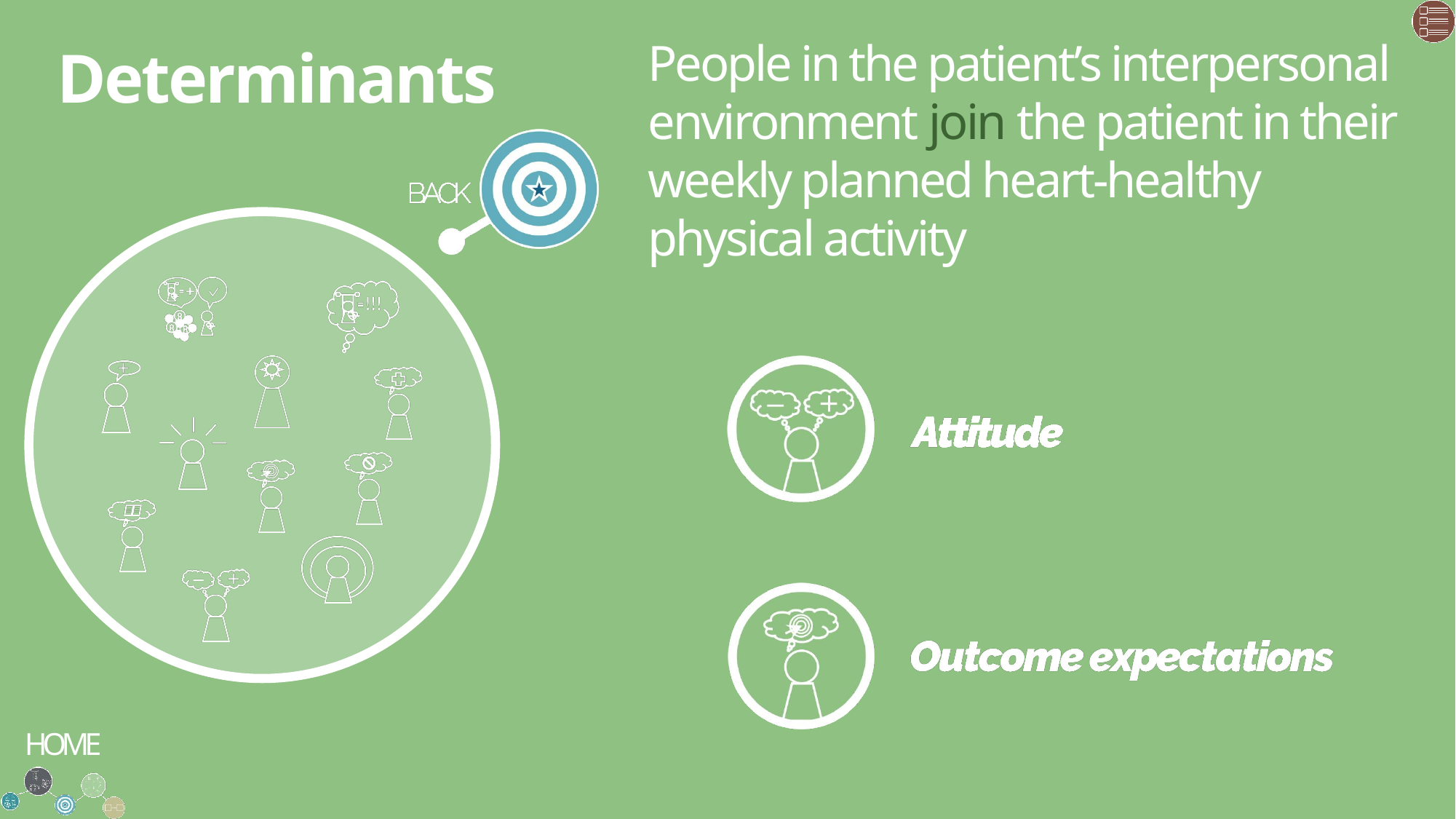

PO2 Determinants for PO2 for people in the patient’s interpersonal environment
People in the patient’s interpersonal environment join the patient in their weekly planned heart-healthy physical activity
Determinants
HOME

## Slide 76
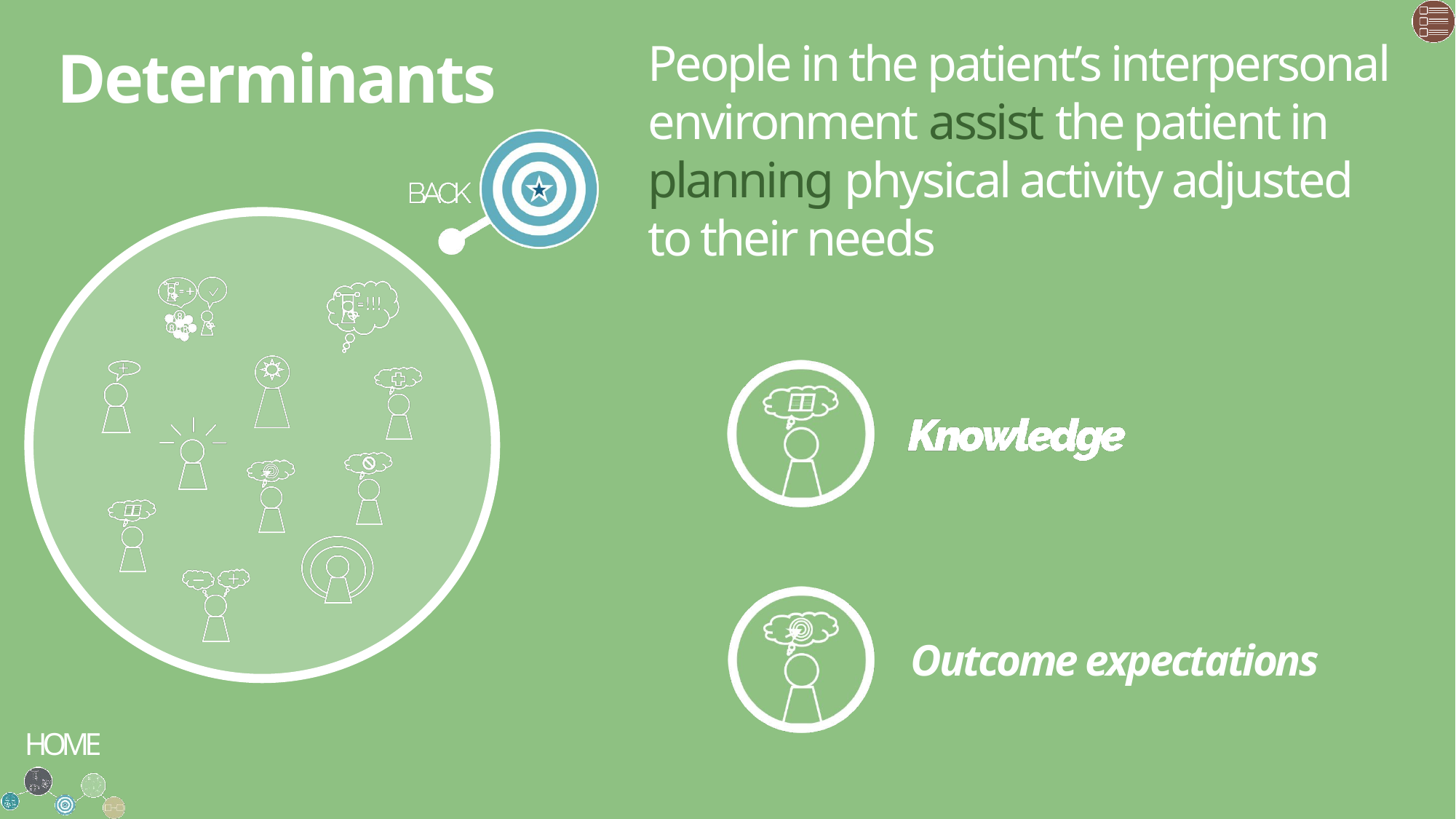

PO3 Determinants for PO3 for people in the patient’s interpersonal environment
People in the patient’s interpersonal environment assist the patient in planning physical activity adjusted to their needs
Determinants
Outcome expectations
HOME

## Slide 77
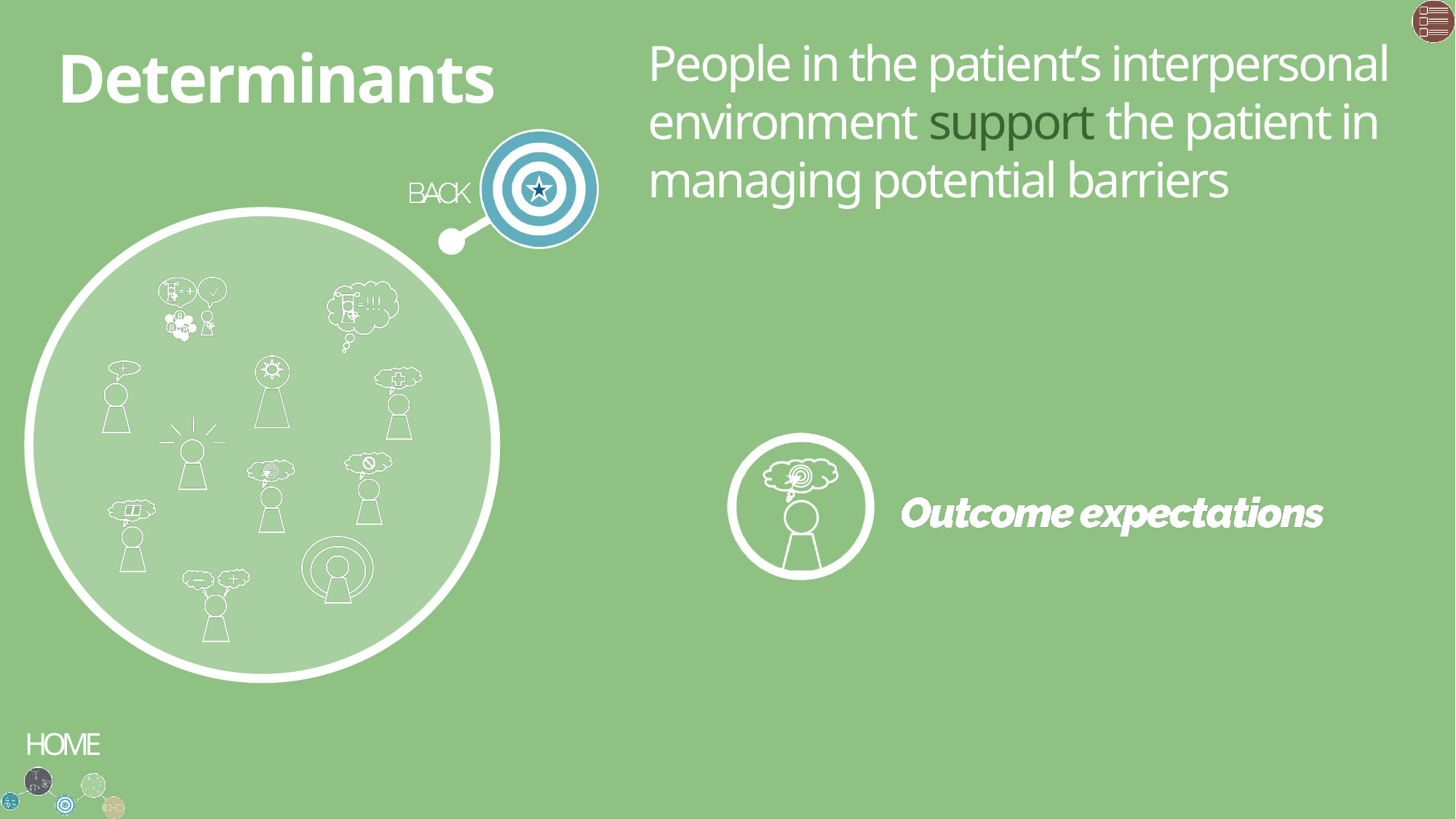

PO4 Determinants for PO4 for people in the patient’s interpersonal environment
People in the patient’s interpersonal environment support the patient in managing potential barriers
Determinants
HOME

## Slide 78
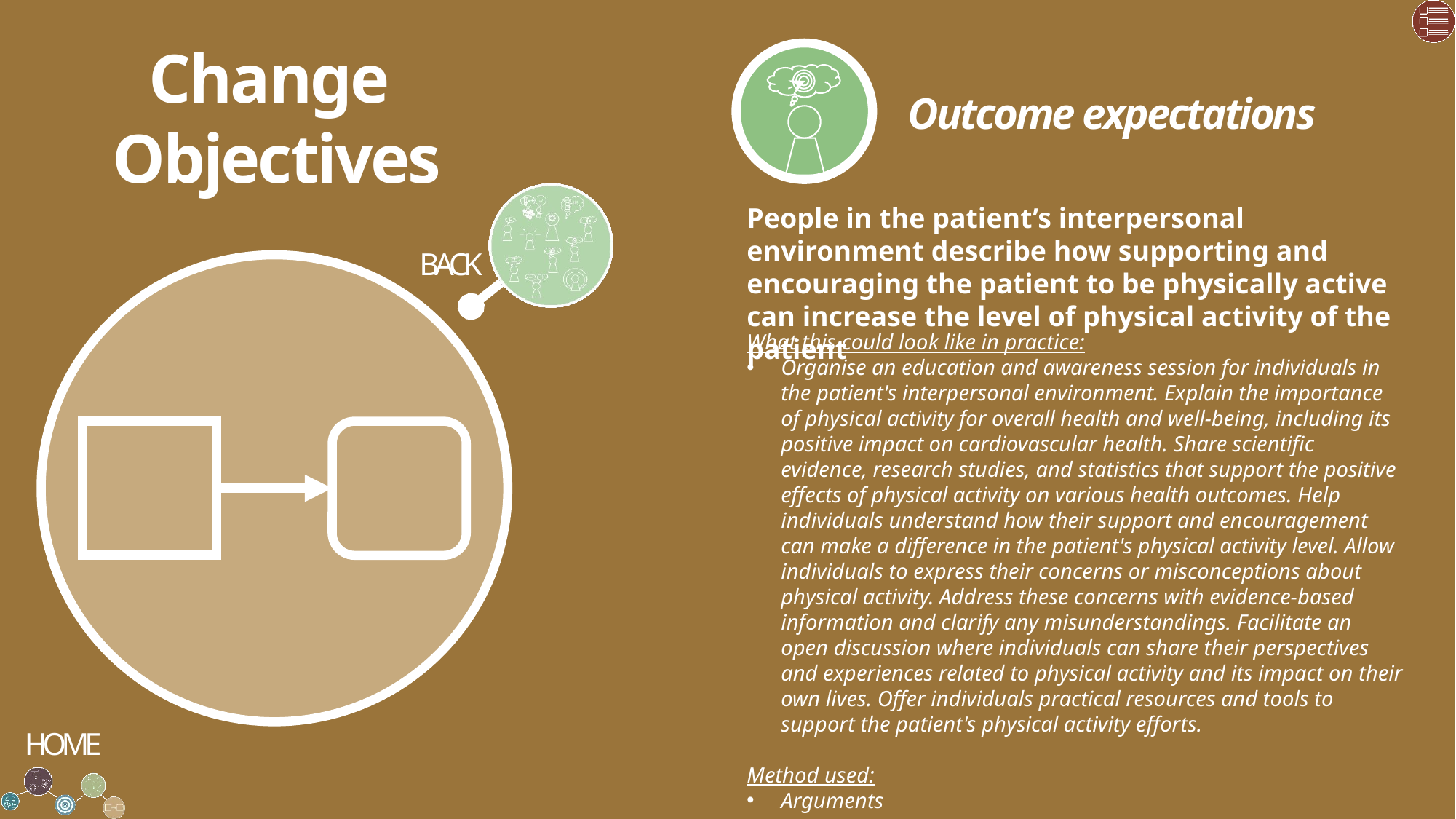

PO1 OE Change Objectives for PO1 for people in the patient’s interpersonal environment
Change
Objectives
Outcome expectations
People in the patient’s interpersonal environment describe how supporting and encouraging the patient to be physically active can increase the level of physical activity of the patient
BACK
What this could look like in practice:
Organise an education and awareness session for individuals in the patient's interpersonal environment. Explain the importance of physical activity for overall health and well-being, including its positive impact on cardiovascular health. Share scientific evidence, research studies, and statistics that support the positive effects of physical activity on various health outcomes. Help individuals understand how their support and encouragement can make a difference in the patient's physical activity level. Allow individuals to express their concerns or misconceptions about physical activity. Address these concerns with evidence-based information and clarify any misunderstandings. Facilitate an open discussion where individuals can share their perspectives and experiences related to physical activity and its impact on their own lives. Offer individuals practical resources and tools to support the patient's physical activity efforts.
Method used:
Arguments
HOME

## Slide 79
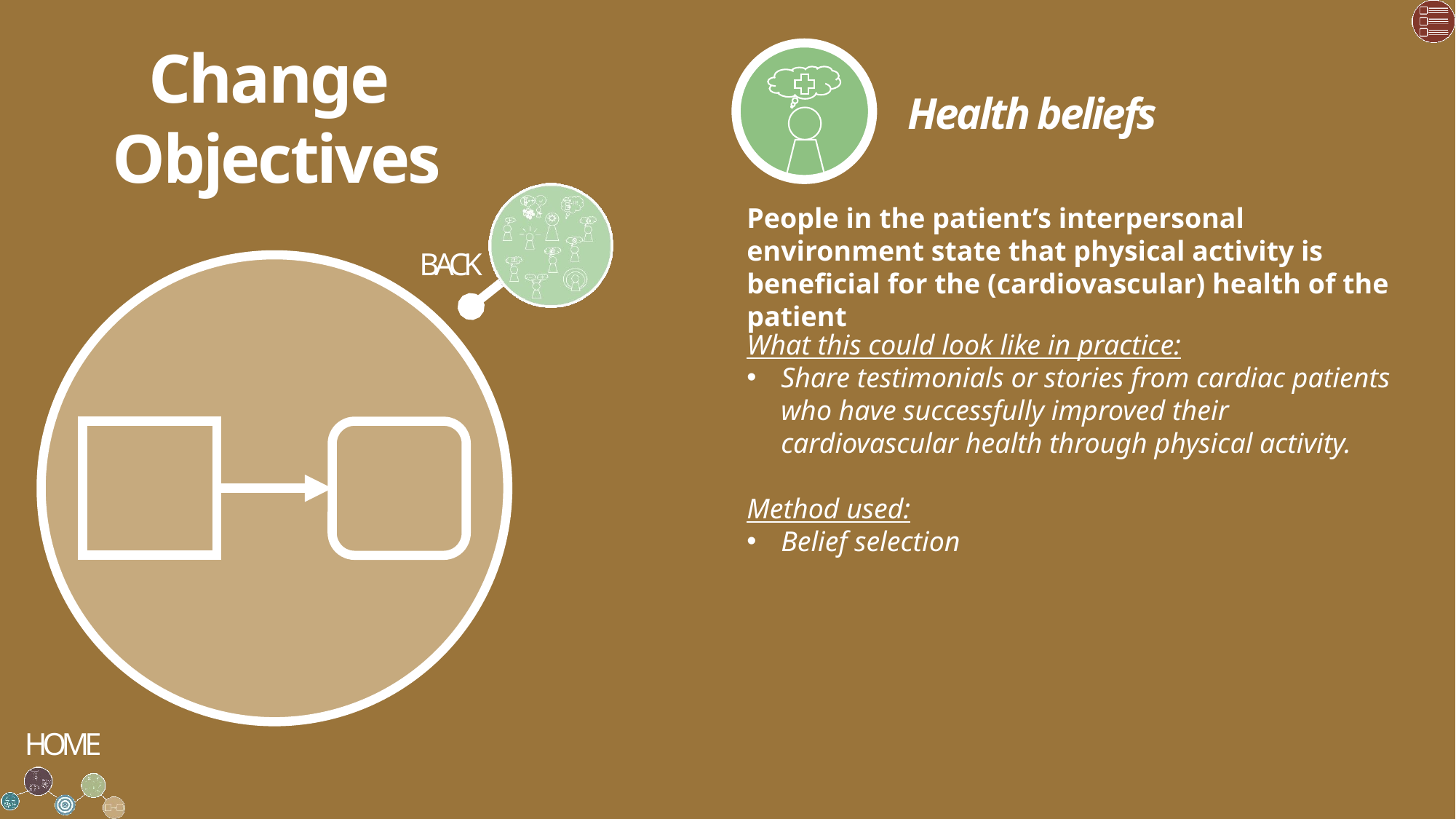

PO1 HB Change Objectives for PO1 for people in the patient’s interpersonal environment
Change
Objectives
Health beliefs
People in the patient’s interpersonal environment state that physical activity is beneficial for the (cardiovascular) health of the patient
BACK
What this could look like in practice:
Share testimonials or stories from cardiac patients who have successfully improved their cardiovascular health through physical activity.
Method used:
Belief selection
HOME

## Slide 80
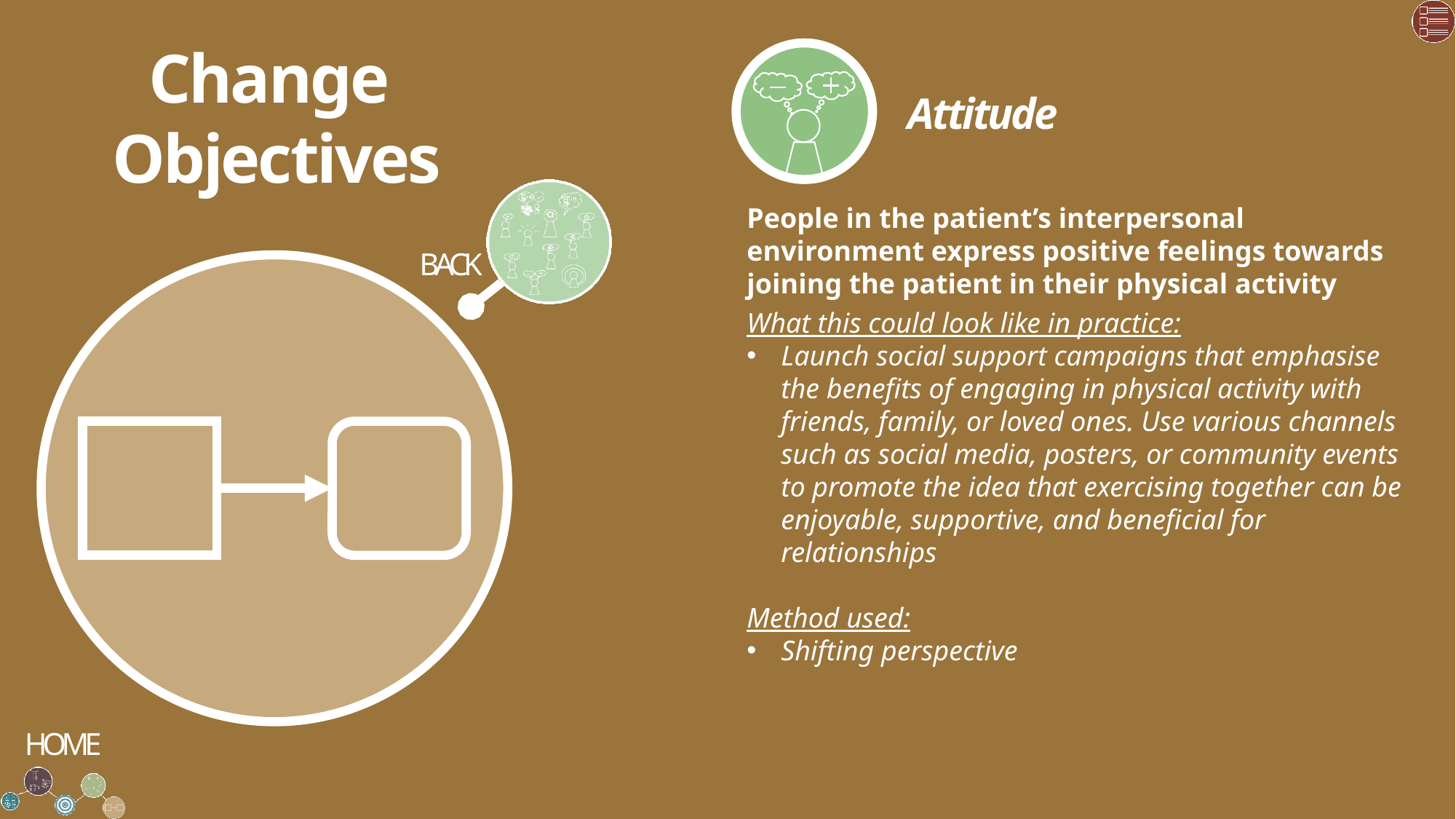

PO2 Att Change Objectives for PO2 for people in the patient’s interpersonal environment
Change
Objectives
Attitude
People in the patient’s interpersonal environment express positive feelings towards joining the patient in their physical activity
BACK
What this could look like in practice:
Launch social support campaigns that emphasise the benefits of engaging in physical activity with friends, family, or loved ones. Use various channels such as social media, posters, or community events to promote the idea that exercising together can be enjoyable, supportive, and beneficial for relationships
Method used:
Shifting perspective
HOME

## Slide 81
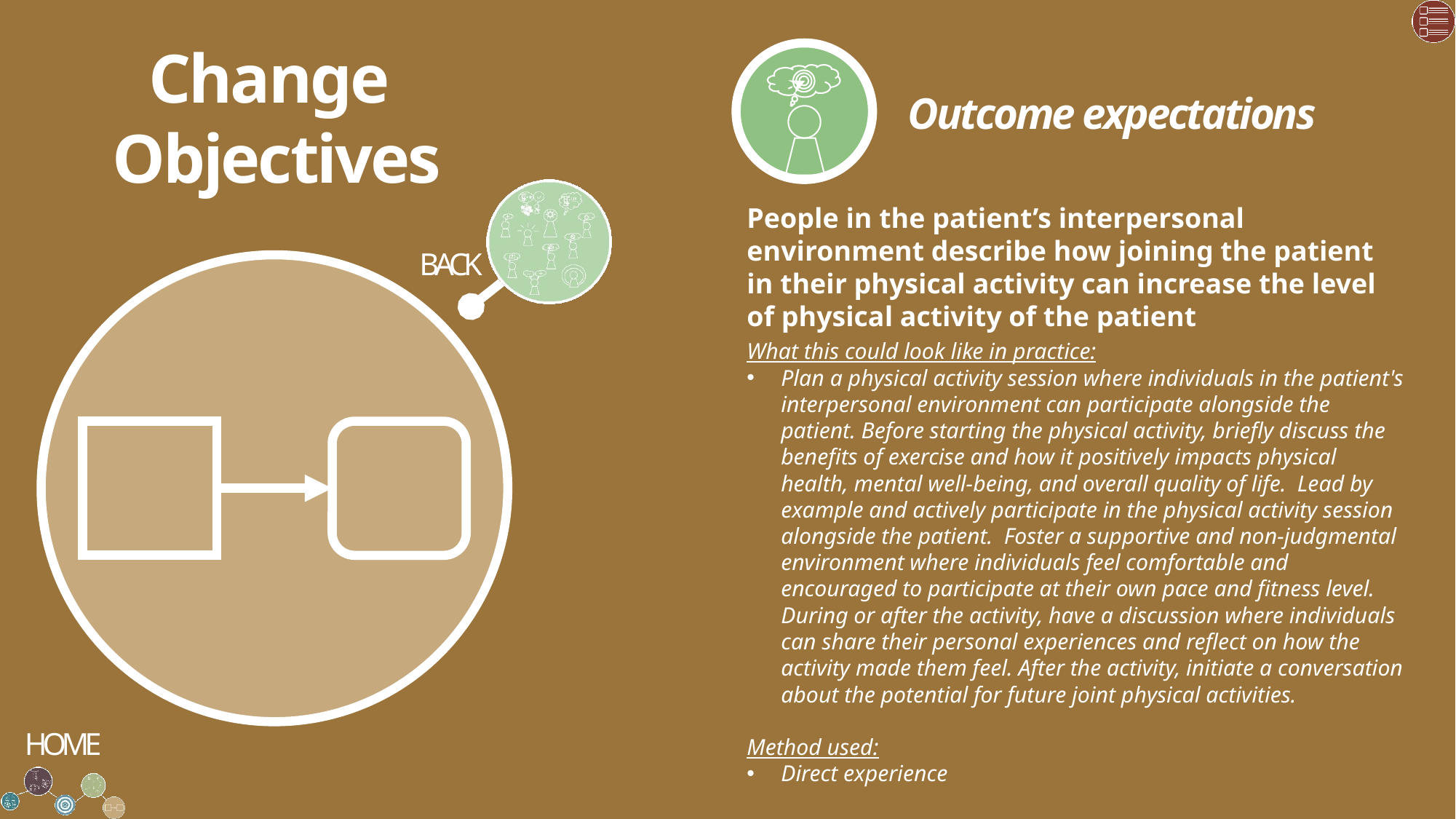

PO2 OE Change Objectives for PO2 for people in the patient’s interpersonal environment
Change
Objectives
Outcome expectations
People in the patient’s interpersonal environment describe how joining the patient in their physical activity can increase the level of physical activity of the patient
BACK
What this could look like in practice:
Plan a physical activity session where individuals in the patient's interpersonal environment can participate alongside the patient. Before starting the physical activity, briefly discuss the benefits of exercise and how it positively impacts physical health, mental well-being, and overall quality of life. Lead by example and actively participate in the physical activity session alongside the patient. Foster a supportive and non-judgmental environment where individuals feel comfortable and encouraged to participate at their own pace and fitness level. During or after the activity, have a discussion where individuals can share their personal experiences and reflect on how the activity made them feel. After the activity, initiate a conversation about the potential for future joint physical activities.
Method used:
Direct experience
HOME

## Slide 82
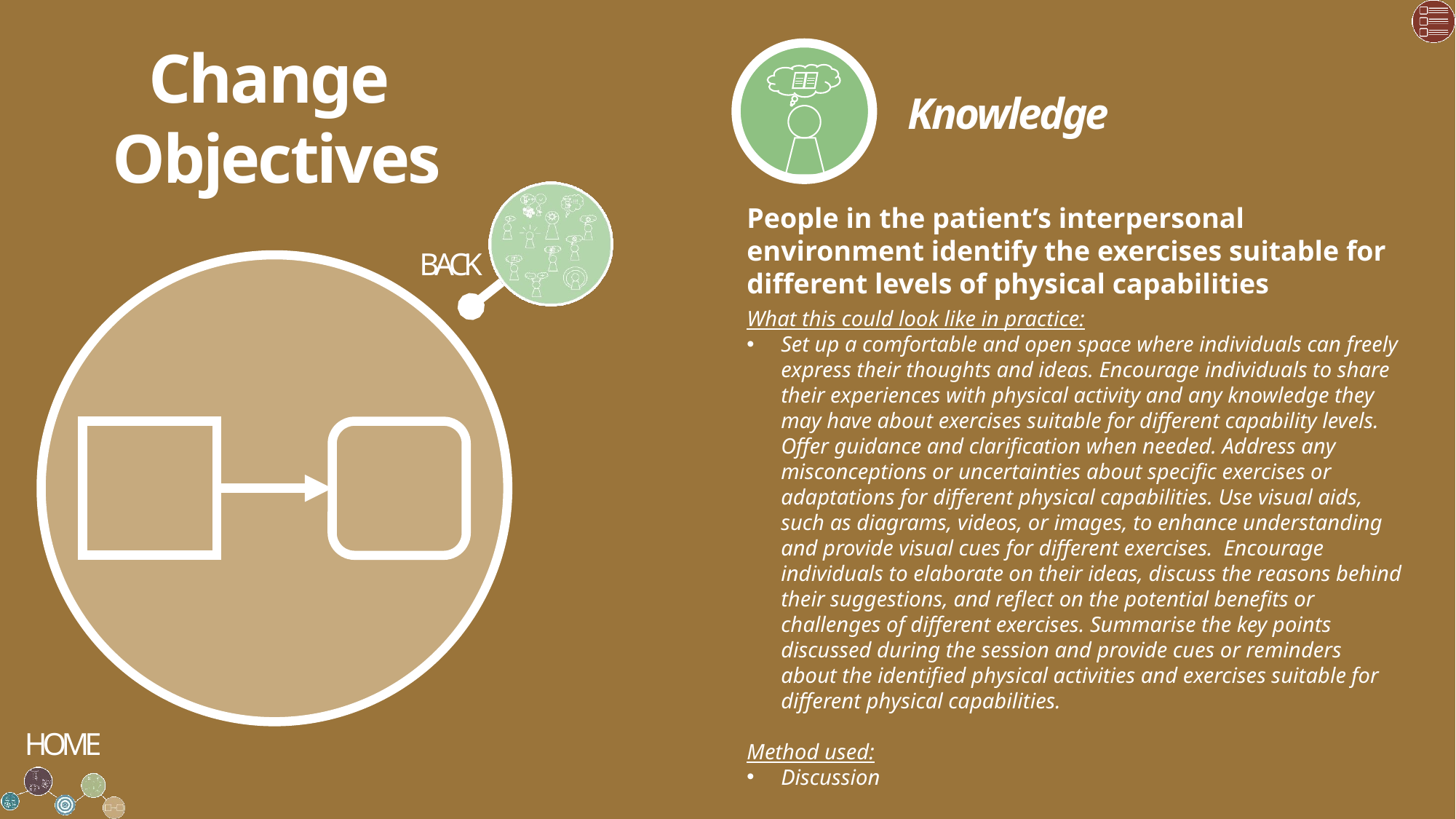

PO3 K Change Objectives for people in the patient’s interpersonal environment
Change
Objectives
Knowledge
People in the patient’s interpersonal environment identify the exercises suitable for different levels of physical capabilities
BACK
What this could look like in practice:
Set up a comfortable and open space where individuals can freely express their thoughts and ideas. Encourage individuals to share their experiences with physical activity and any knowledge they may have about exercises suitable for different capability levels. Offer guidance and clarification when needed. Address any misconceptions or uncertainties about specific exercises or adaptations for different physical capabilities. Use visual aids, such as diagrams, videos, or images, to enhance understanding and provide visual cues for different exercises. Encourage individuals to elaborate on their ideas, discuss the reasons behind their suggestions, and reflect on the potential benefits or challenges of different exercises. Summarise the key points discussed during the session and provide cues or reminders about the identified physical activities and exercises suitable for different physical capabilities.
Method used:
Discussion
HOME

## Slide 83
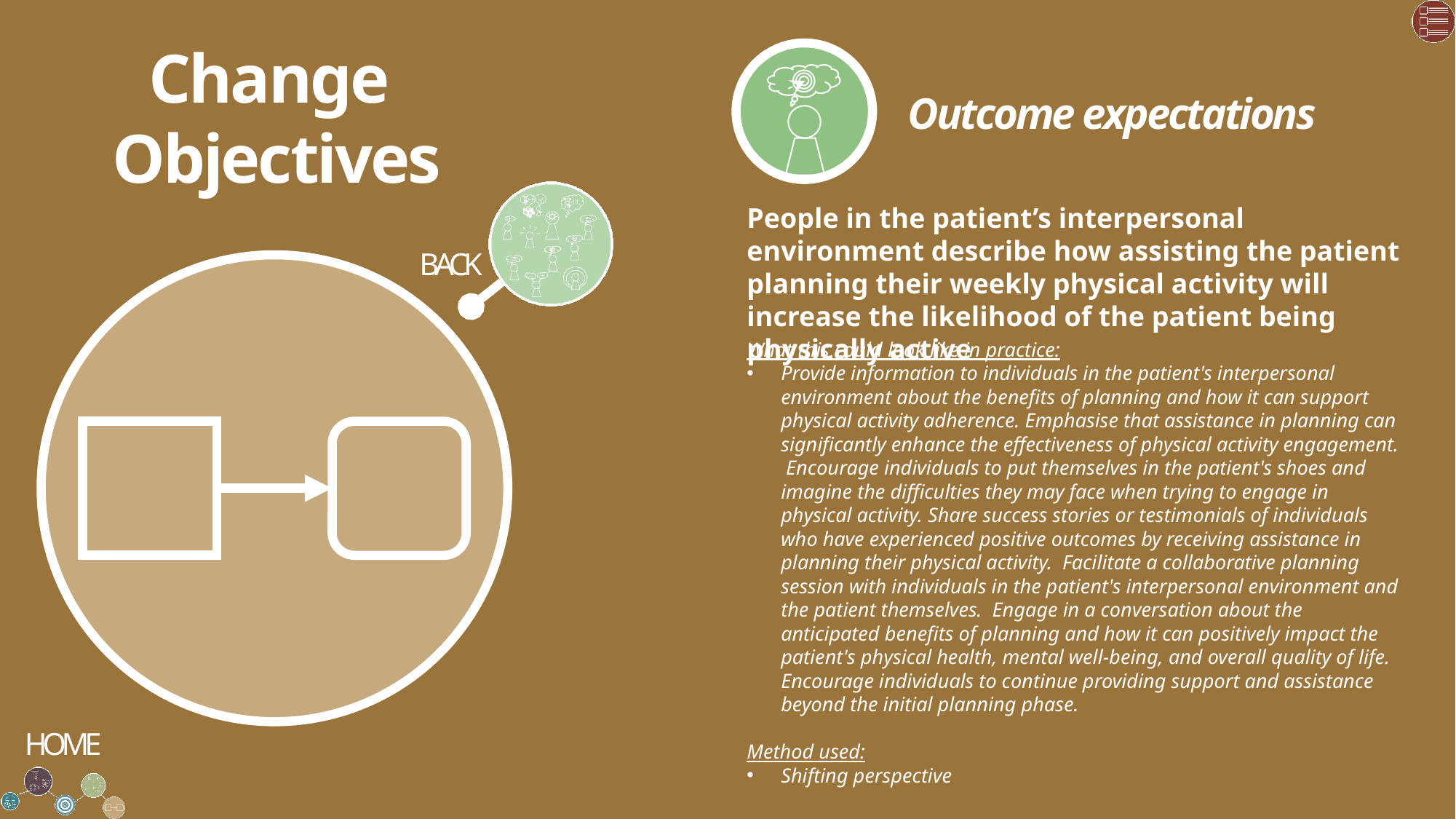

PO3 OE Change Objectives for people in the patient’s interpersonal environment
Change
Objectives
Outcome expectations
People in the patient’s interpersonal environment describe how assisting the patient planning their weekly physical activity will increase the likelihood of the patient being physically active
BACK
What this could look like in practice:
Provide information to individuals in the patient's interpersonal environment about the benefits of planning and how it can support physical activity adherence. Emphasise that assistance in planning can significantly enhance the effectiveness of physical activity engagement. Encourage individuals to put themselves in the patient's shoes and imagine the difficulties they may face when trying to engage in physical activity. Share success stories or testimonials of individuals who have experienced positive outcomes by receiving assistance in planning their physical activity. Facilitate a collaborative planning session with individuals in the patient's interpersonal environment and the patient themselves. Engage in a conversation about the anticipated benefits of planning and how it can positively impact the patient's physical health, mental well-being, and overall quality of life. Encourage individuals to continue providing support and assistance beyond the initial planning phase.
Method used:
Shifting perspective
HOME

## Slide 84
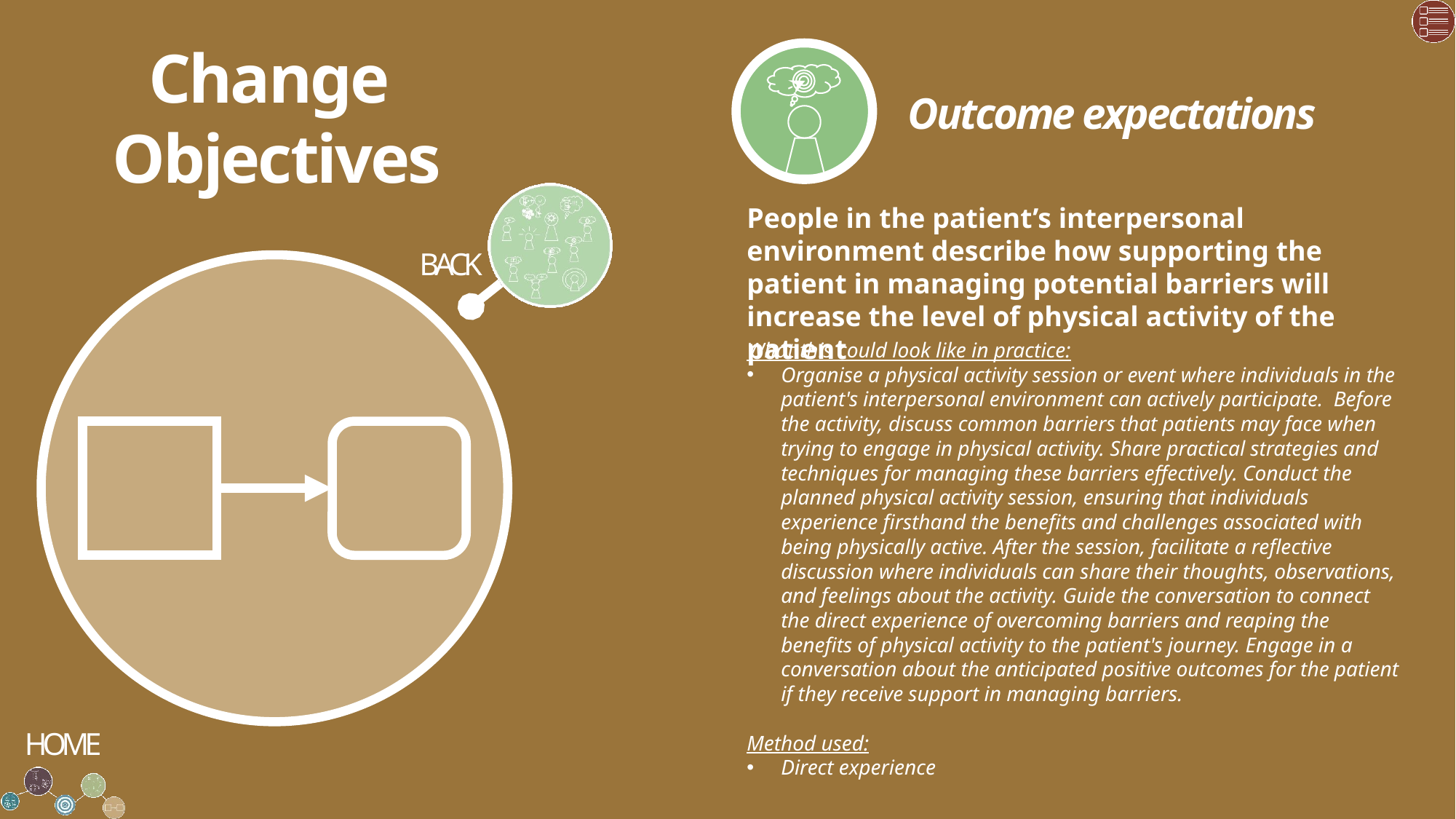

PO4 OE Change Objectives for people in the patient’s interpersonal environment
Change
Objectives
Outcome expectations
People in the patient’s interpersonal environment describe how supporting the patient in managing potential barriers will increase the level of physical activity of the patient
BACK
What this could look like in practice:
Organise a physical activity session or event where individuals in the patient's interpersonal environment can actively participate. Before the activity, discuss common barriers that patients may face when trying to engage in physical activity. Share practical strategies and techniques for managing these barriers effectively. Conduct the planned physical activity session, ensuring that individuals experience firsthand the benefits and challenges associated with being physically active. After the session, facilitate a reflective discussion where individuals can share their thoughts, observations, and feelings about the activity. Guide the conversation to connect the direct experience of overcoming barriers and reaping the benefits of physical activity to the patient's journey. Engage in a conversation about the anticipated positive outcomes for the patient if they receive support in managing barriers.
Method used:
Direct experience
HOME

## Slide 85
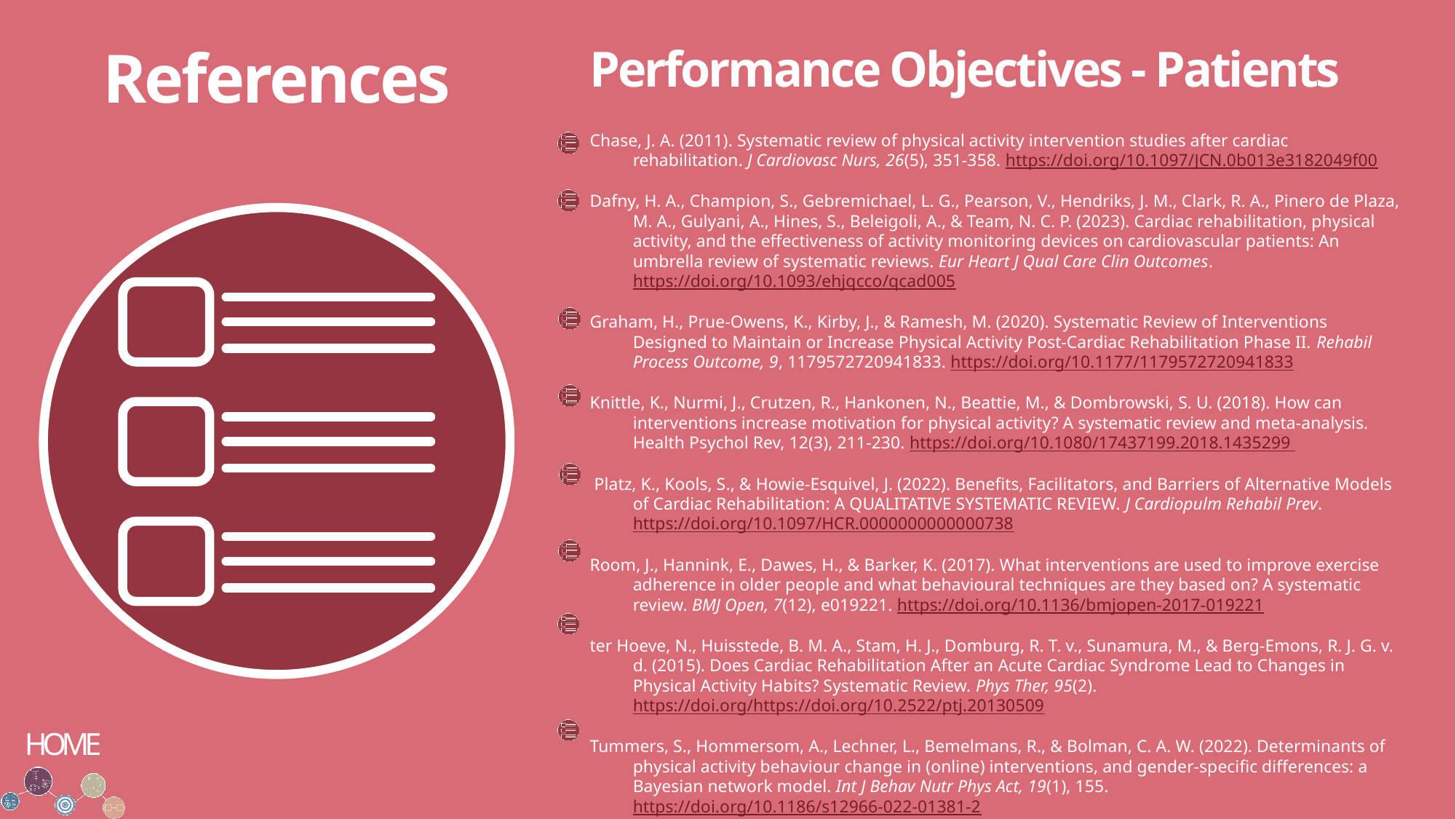

Reference list POs for patients
References
Performance Objectives - Patients
Chase, J. A. (2011). Systematic review of physical activity intervention studies after cardiac rehabilitation. J Cardiovasc Nurs, 26(5), 351-358. https://doi.org/10.1097/JCN.0b013e3182049f00
Dafny, H. A., Champion, S., Gebremichael, L. G., Pearson, V., Hendriks, J. M., Clark, R. A., Pinero de Plaza, M. A., Gulyani, A., Hines, S., Beleigoli, A., & Team, N. C. P. (2023). Cardiac rehabilitation, physical activity, and the effectiveness of activity monitoring devices on cardiovascular patients: An umbrella review of systematic reviews. Eur Heart J Qual Care Clin Outcomes. https://doi.org/10.1093/ehjqcco/qcad005
Graham, H., Prue-Owens, K., Kirby, J., & Ramesh, M. (2020). Systematic Review of Interventions Designed to Maintain or Increase Physical Activity Post-Cardiac Rehabilitation Phase II. Rehabil Process Outcome, 9, 1179572720941833. https://doi.org/10.1177/1179572720941833
Knittle, K., Nurmi, J., Crutzen, R., Hankonen, N., Beattie, M., & Dombrowski, S. U. (2018). How can interventions increase motivation for physical activity? A systematic review and meta-analysis. Health Psychol Rev, 12(3), 211-230. https://doi.org/10.1080/17437199.2018.1435299
 Platz, K., Kools, S., & Howie-Esquivel, J. (2022). Benefits, Facilitators, and Barriers of Alternative Models of Cardiac Rehabilitation: A QUALITATIVE SYSTEMATIC REVIEW. J Cardiopulm Rehabil Prev. https://doi.org/10.1097/HCR.0000000000000738
Room, J., Hannink, E., Dawes, H., & Barker, K. (2017). What interventions are used to improve exercise adherence in older people and what behavioural techniques are they based on? A systematic review. BMJ Open, 7(12), e019221. https://doi.org/10.1136/bmjopen-2017-019221
ter Hoeve, N., Huisstede, B. M. A., Stam, H. J., Domburg, R. T. v., Sunamura, M., & Berg-Emons, R. J. G. v. d. (2015). Does Cardiac Rehabilitation After an Acute Cardiac Syndrome Lead to Changes in Physical Activity Habits? Systematic Review. Phys Ther, 95(2). https://doi.org/https://doi.org/10.2522/ptj.20130509
Tummers, S., Hommersom, A., Lechner, L., Bemelmans, R., & Bolman, C. A. W. (2022). Determinants of physical activity behaviour change in (online) interventions, and gender-specific differences: a Bayesian network model. Int J Behav Nutr Phys Act, 19(1), 155. https://doi.org/10.1186/s12966-022-01381-2
HOME

## Slide 86
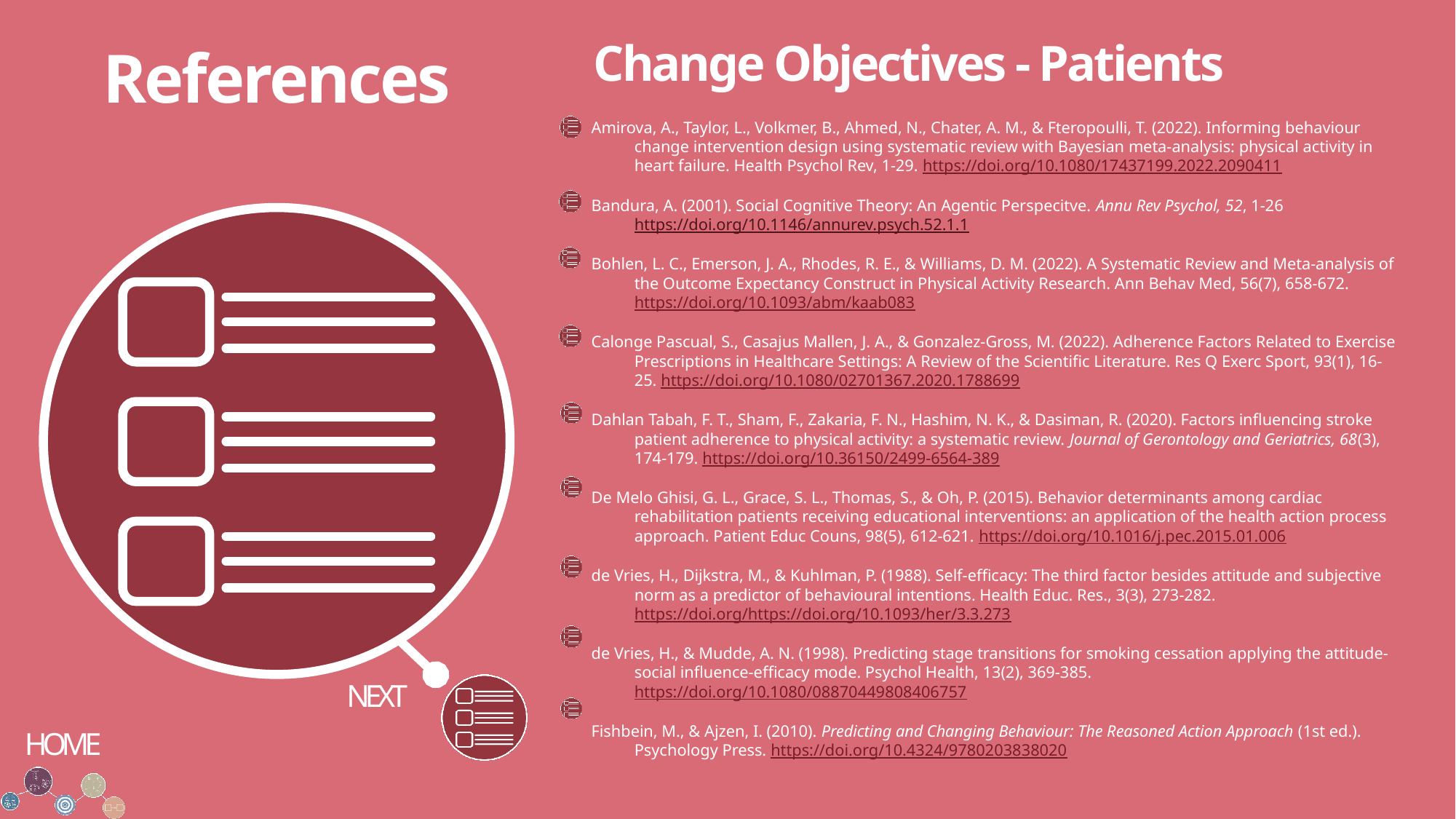

Reference list COs for patients
Change Objectives - Patients
References
Amirova, A., Taylor, L., Volkmer, B., Ahmed, N., Chater, A. M., & Fteropoulli, T. (2022). Informing behaviour change intervention design using systematic review with Bayesian meta-analysis: physical activity in heart failure. Health Psychol Rev, 1-29. https://doi.org/10.1080/17437199.2022.2090411
Bandura, A. (2001). Social Cognitive Theory: An Agentic Perspecitve. Annu Rev Psychol, 52, 1-26 https://doi.org/10.1146/annurev.psych.52.1.1
Bohlen, L. C., Emerson, J. A., Rhodes, R. E., & Williams, D. M. (2022). A Systematic Review and Meta-analysis of the Outcome Expectancy Construct in Physical Activity Research. Ann Behav Med, 56(7), 658-672. https://doi.org/10.1093/abm/kaab083
Calonge Pascual, S., Casajus Mallen, J. A., & Gonzalez-Gross, M. (2022). Adherence Factors Related to Exercise Prescriptions in Healthcare Settings: A Review of the Scientific Literature. Res Q Exerc Sport, 93(1), 16-25. https://doi.org/10.1080/02701367.2020.1788699
Dahlan Tabah, F. T., Sham, F., Zakaria, F. N., Hashim, N. K., & Dasiman, R. (2020). Factors influencing stroke patient adherence to physical activity: a systematic review. Journal of Gerontology and Geriatrics, 68(3), 174-179. https://doi.org/10.36150/2499-6564-389
De Melo Ghisi, G. L., Grace, S. L., Thomas, S., & Oh, P. (2015). Behavior determinants among cardiac rehabilitation patients receiving educational interventions: an application of the health action process approach. Patient Educ Couns, 98(5), 612-621. https://doi.org/10.1016/j.pec.2015.01.006
de Vries, H., Dijkstra, M., & Kuhlman, P. (1988). Self-efficacy: The third factor besides attitude and subjective norm as a predictor of behavioural intentions. Health Educ. Res., 3(3), 273-282. https://doi.org/https://doi.org/10.1093/her/3.3.273
de Vries, H., & Mudde, A. N. (1998). Predicting stage transitions for smoking cessation applying the attitude-social influence-efficacy mode. Psychol Health, 13(2), 369-385. https://doi.org/10.1080/08870449808406757
Fishbein, M., & Ajzen, I. (2010). Predicting and Changing Behaviour: The Reasoned Action Approach (1st ed.). Psychology Press. https://doi.org/10.4324/9780203838020
NEXT
HOME

## Slide 87
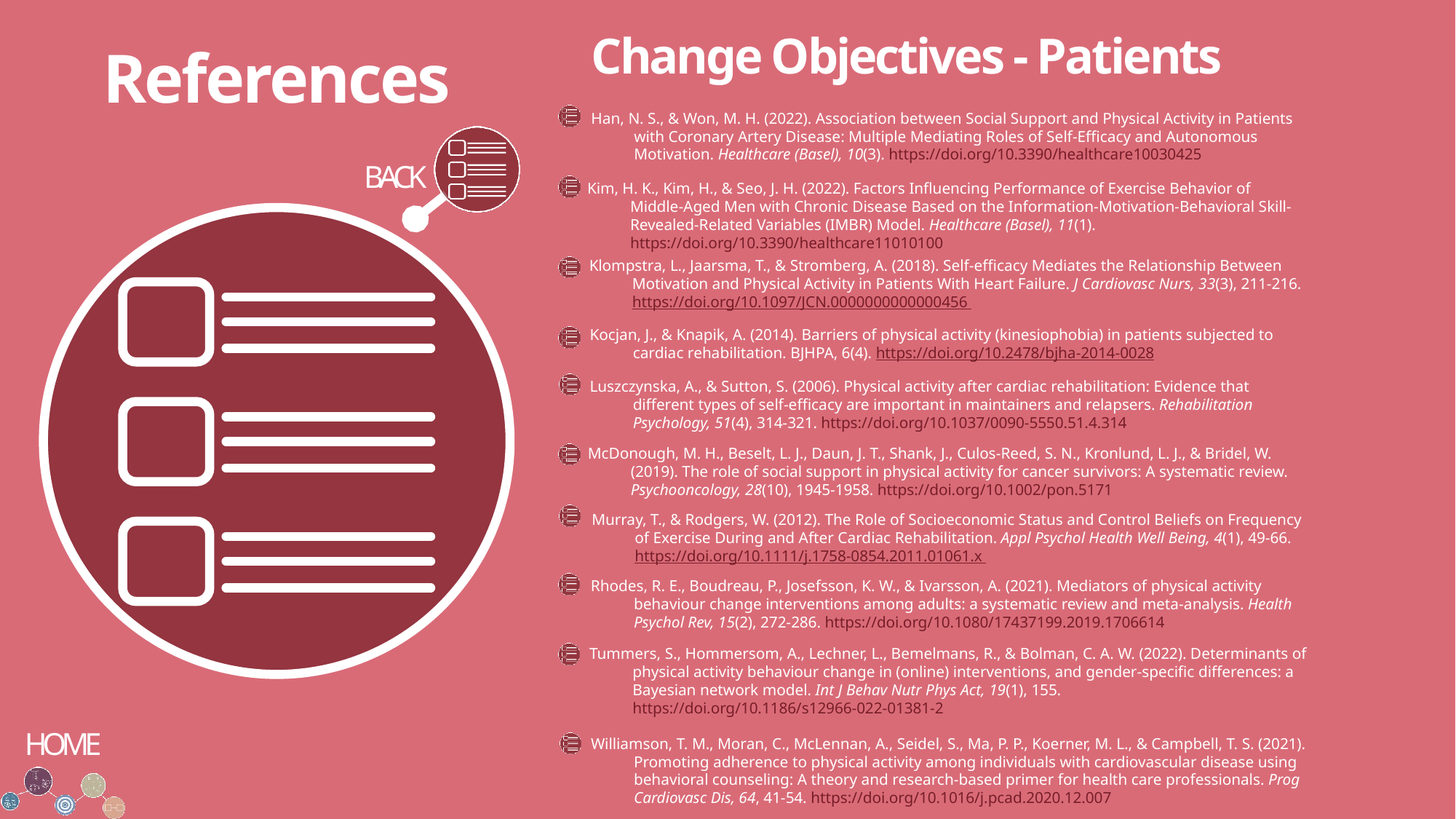

Reference list COs for patients
Change Objectives - Patients
References
Han, N. S., & Won, M. H. (2022). Association between Social Support and Physical Activity in Patients with Coronary Artery Disease: Multiple Mediating Roles of Self-Efficacy and Autonomous Motivation. Healthcare (Basel), 10(3). https://doi.org/10.3390/healthcare10030425
BACK
Kim, H. K., Kim, H., & Seo, J. H. (2022). Factors Influencing Performance of Exercise Behavior of Middle-Aged Men with Chronic Disease Based on the Information-Motivation-Behavioral Skill-Revealed-Related Variables (IMBR) Model. Healthcare (Basel), 11(1). https://doi.org/10.3390/healthcare11010100
Klompstra, L., Jaarsma, T., & Stromberg, A. (2018). Self-efficacy Mediates the Relationship Between Motivation and Physical Activity in Patients With Heart Failure. J Cardiovasc Nurs, 33(3), 211-216. https://doi.org/10.1097/JCN.0000000000000456
Kocjan, J., & Knapik, A. (2014). Barriers of physical activity (kinesiophobia) in patients subjected to cardiac rehabilitation. BJHPA, 6(4). https://doi.org/10.2478/bjha-2014-0028
Luszczynska, A., & Sutton, S. (2006). Physical activity after cardiac rehabilitation: Evidence that different types of self-efficacy are important in maintainers and relapsers. Rehabilitation Psychology, 51(4), 314-321. https://doi.org/10.1037/0090-5550.51.4.314
McDonough, M. H., Beselt, L. J., Daun, J. T., Shank, J., Culos-Reed, S. N., Kronlund, L. J., & Bridel, W. (2019). The role of social support in physical activity for cancer survivors: A systematic review. Psychooncology, 28(10), 1945-1958. https://doi.org/10.1002/pon.5171
Murray, T., & Rodgers, W. (2012). The Role of Socioeconomic Status and Control Beliefs on Frequency of Exercise During and After Cardiac Rehabilitation. Appl Psychol Health Well Being, 4(1), 49-66. https://doi.org/10.1111/j.1758-0854.2011.01061.x
Rhodes, R. E., Boudreau, P., Josefsson, K. W., & Ivarsson, A. (2021). Mediators of physical activity behaviour change interventions among adults: a systematic review and meta-analysis. Health Psychol Rev, 15(2), 272-286. https://doi.org/10.1080/17437199.2019.1706614
Tummers, S., Hommersom, A., Lechner, L., Bemelmans, R., & Bolman, C. A. W. (2022). Determinants of physical activity behaviour change in (online) interventions, and gender-specific differences: a Bayesian network model. Int J Behav Nutr Phys Act, 19(1), 155. https://doi.org/10.1186/s12966-022-01381-2
HOME
Williamson, T. M., Moran, C., McLennan, A., Seidel, S., Ma, P. P., Koerner, M. L., & Campbell, T. S. (2021). Promoting adherence to physical activity among individuals with cardiovascular disease using behavioral counseling: A theory and research-based primer for health care professionals. Prog Cardiovasc Dis, 64, 41-54. https://doi.org/10.1016/j.pcad.2020.12.007

## Slide 88
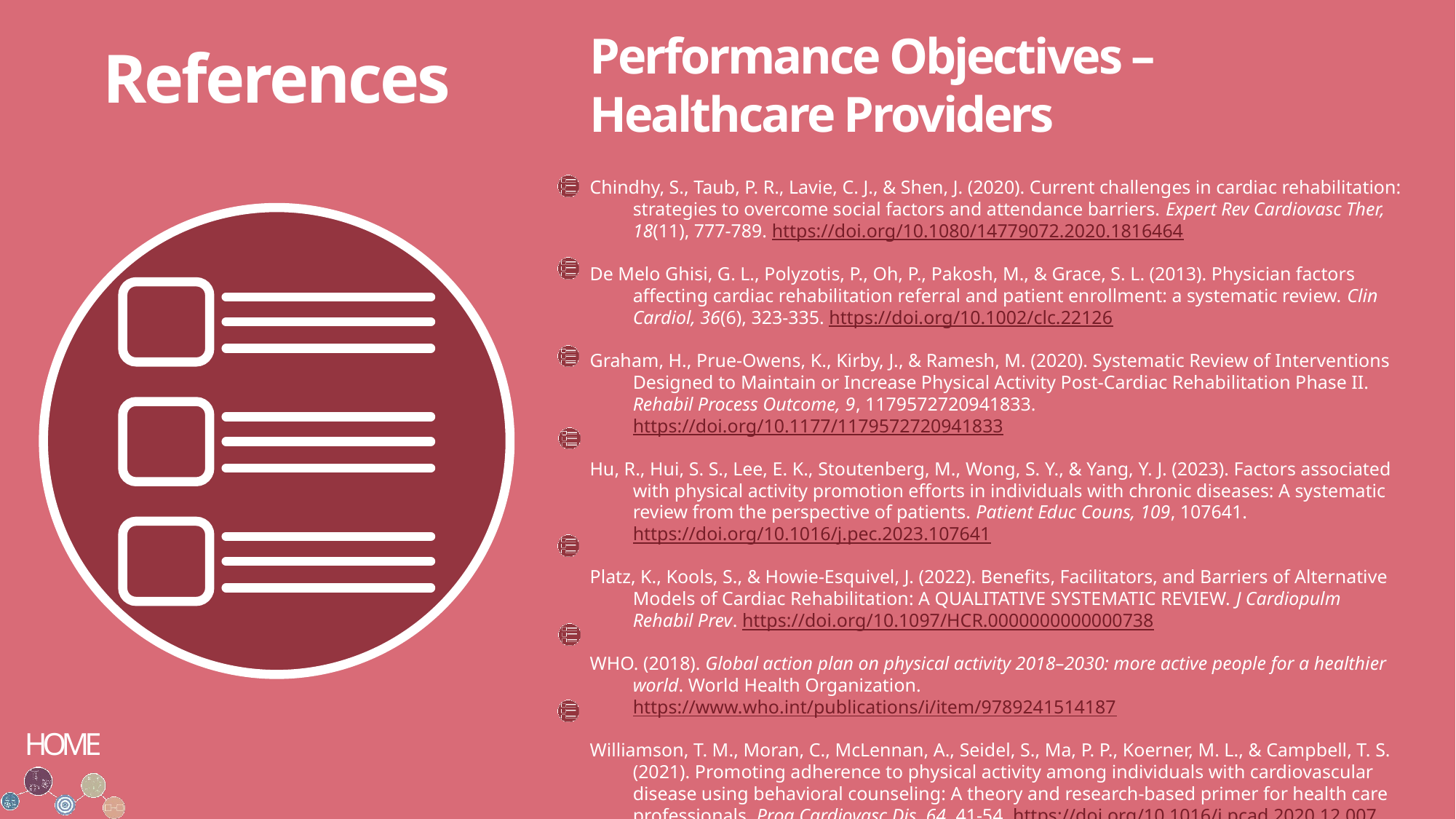

Reference list POs for HCP
Performance Objectives –
Healthcare Providers
References
Chindhy, S., Taub, P. R., Lavie, C. J., & Shen, J. (2020). Current challenges in cardiac rehabilitation: strategies to overcome social factors and attendance barriers. Expert Rev Cardiovasc Ther, 18(11), 777-789. https://doi.org/10.1080/14779072.2020.1816464
De Melo Ghisi, G. L., Polyzotis, P., Oh, P., Pakosh, M., & Grace, S. L. (2013). Physician factors affecting cardiac rehabilitation referral and patient enrollment: a systematic review. Clin Cardiol, 36(6), 323-335. https://doi.org/10.1002/clc.22126
Graham, H., Prue-Owens, K., Kirby, J., & Ramesh, M. (2020). Systematic Review of Interventions Designed to Maintain or Increase Physical Activity Post-Cardiac Rehabilitation Phase II. Rehabil Process Outcome, 9, 1179572720941833. https://doi.org/10.1177/1179572720941833
Hu, R., Hui, S. S., Lee, E. K., Stoutenberg, M., Wong, S. Y., & Yang, Y. J. (2023). Factors associated with physical activity promotion efforts in individuals with chronic diseases: A systematic review from the perspective of patients. Patient Educ Couns, 109, 107641. https://doi.org/10.1016/j.pec.2023.107641
Platz, K., Kools, S., & Howie-Esquivel, J. (2022). Benefits, Facilitators, and Barriers of Alternative Models of Cardiac Rehabilitation: A QUALITATIVE SYSTEMATIC REVIEW. J Cardiopulm Rehabil Prev. https://doi.org/10.1097/HCR.0000000000000738
WHO. (2018). Global action plan on physical activity 2018–2030: more active people for a healthier world. World Health Organization. https://www.who.int/publications/i/item/9789241514187
Williamson, T. M., Moran, C., McLennan, A., Seidel, S., Ma, P. P., Koerner, M. L., & Campbell, T. S. (2021). Promoting adherence to physical activity among individuals with cardiovascular disease using behavioral counseling: A theory and research-based primer for health care professionals. Prog Cardiovasc Dis, 64, 41-54. https://doi.org/10.1016/j.pcad.2020.12.007
HOME

## Slide 89
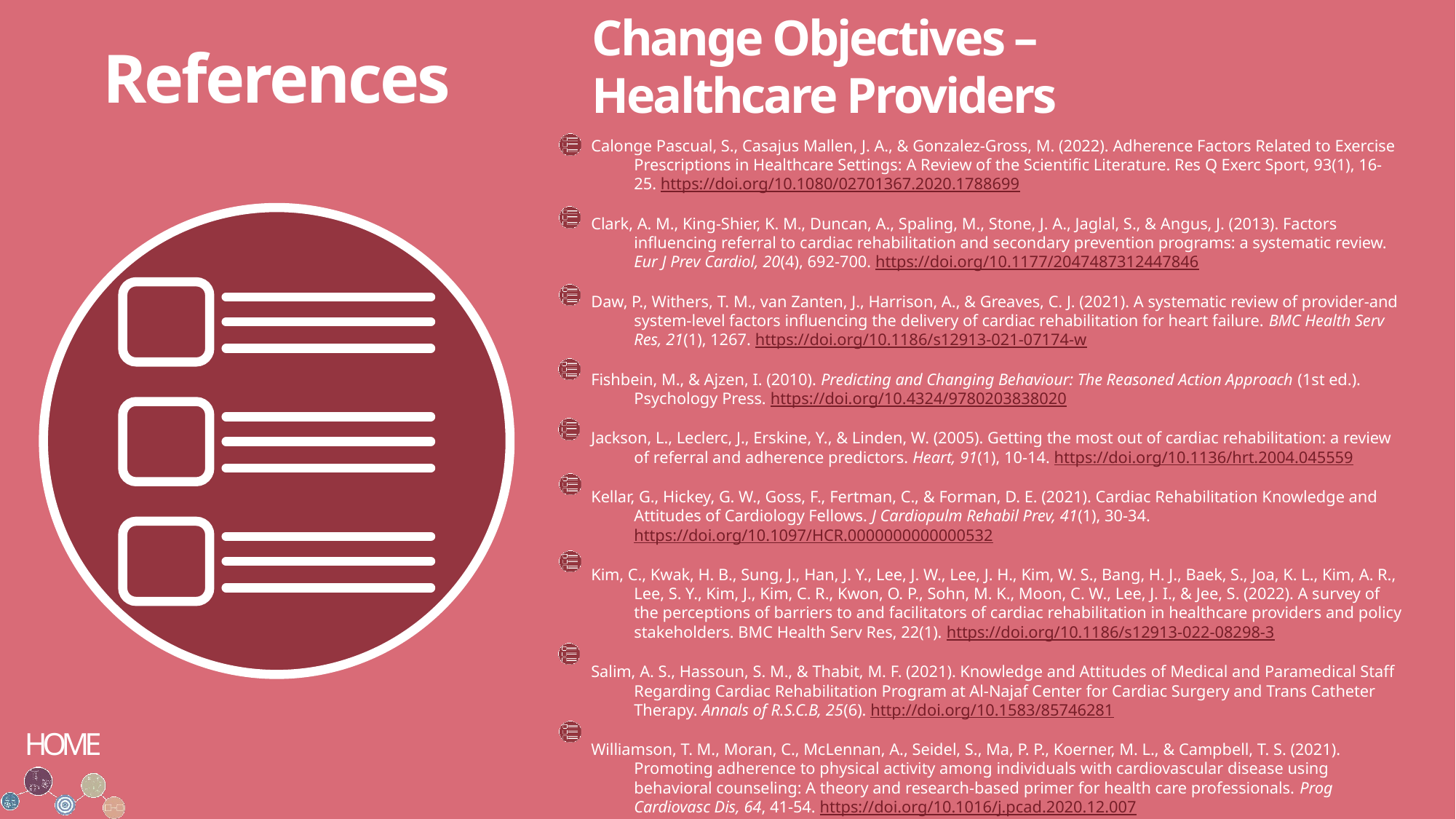

Reference List COs for HCP
Change Objectives –
Healthcare Providers
References
Calonge Pascual, S., Casajus Mallen, J. A., & Gonzalez-Gross, M. (2022). Adherence Factors Related to Exercise Prescriptions in Healthcare Settings: A Review of the Scientific Literature. Res Q Exerc Sport, 93(1), 16-25. https://doi.org/10.1080/02701367.2020.1788699
Clark, A. M., King-Shier, K. M., Duncan, A., Spaling, M., Stone, J. A., Jaglal, S., & Angus, J. (2013). Factors influencing referral to cardiac rehabilitation and secondary prevention programs: a systematic review. Eur J Prev Cardiol, 20(4), 692-700. https://doi.org/10.1177/2047487312447846
Daw, P., Withers, T. M., van Zanten, J., Harrison, A., & Greaves, C. J. (2021). A systematic review of provider-and system-level factors influencing the delivery of cardiac rehabilitation for heart failure. BMC Health Serv Res, 21(1), 1267. https://doi.org/10.1186/s12913-021-07174-w
Fishbein, M., & Ajzen, I. (2010). Predicting and Changing Behaviour: The Reasoned Action Approach (1st ed.). Psychology Press. https://doi.org/10.4324/9780203838020
Jackson, L., Leclerc, J., Erskine, Y., & Linden, W. (2005). Getting the most out of cardiac rehabilitation: a review of referral and adherence predictors. Heart, 91(1), 10-14. https://doi.org/10.1136/hrt.2004.045559
Kellar, G., Hickey, G. W., Goss, F., Fertman, C., & Forman, D. E. (2021). Cardiac Rehabilitation Knowledge and Attitudes of Cardiology Fellows. J Cardiopulm Rehabil Prev, 41(1), 30-34. https://doi.org/10.1097/HCR.0000000000000532
Kim, C., Kwak, H. B., Sung, J., Han, J. Y., Lee, J. W., Lee, J. H., Kim, W. S., Bang, H. J., Baek, S., Joa, K. L., Kim, A. R., Lee, S. Y., Kim, J., Kim, C. R., Kwon, O. P., Sohn, M. K., Moon, C. W., Lee, J. I., & Jee, S. (2022). A survey of the perceptions of barriers to and facilitators of cardiac rehabilitation in healthcare providers and policy stakeholders. BMC Health Serv Res, 22(1). https://doi.org/10.1186/s12913-022-08298-3
Salim, A. S., Hassoun, S. M., & Thabit, M. F. (2021). Knowledge and Attitudes of Medical and Paramedical Staff Regarding Cardiac Rehabilitation Program at Al-Najaf Center for Cardiac Surgery and Trans Catheter Therapy. Annals of R.S.C.B, 25(6). http://doi.org/10.1583/85746281
Williamson, T. M., Moran, C., McLennan, A., Seidel, S., Ma, P. P., Koerner, M. L., & Campbell, T. S. (2021). Promoting adherence to physical activity among individuals with cardiovascular disease using behavioral counseling: A theory and research-based primer for health care professionals. Prog Cardiovasc Dis, 64, 41-54. https://doi.org/10.1016/j.pcad.2020.12.007
HOME

## Slide 90
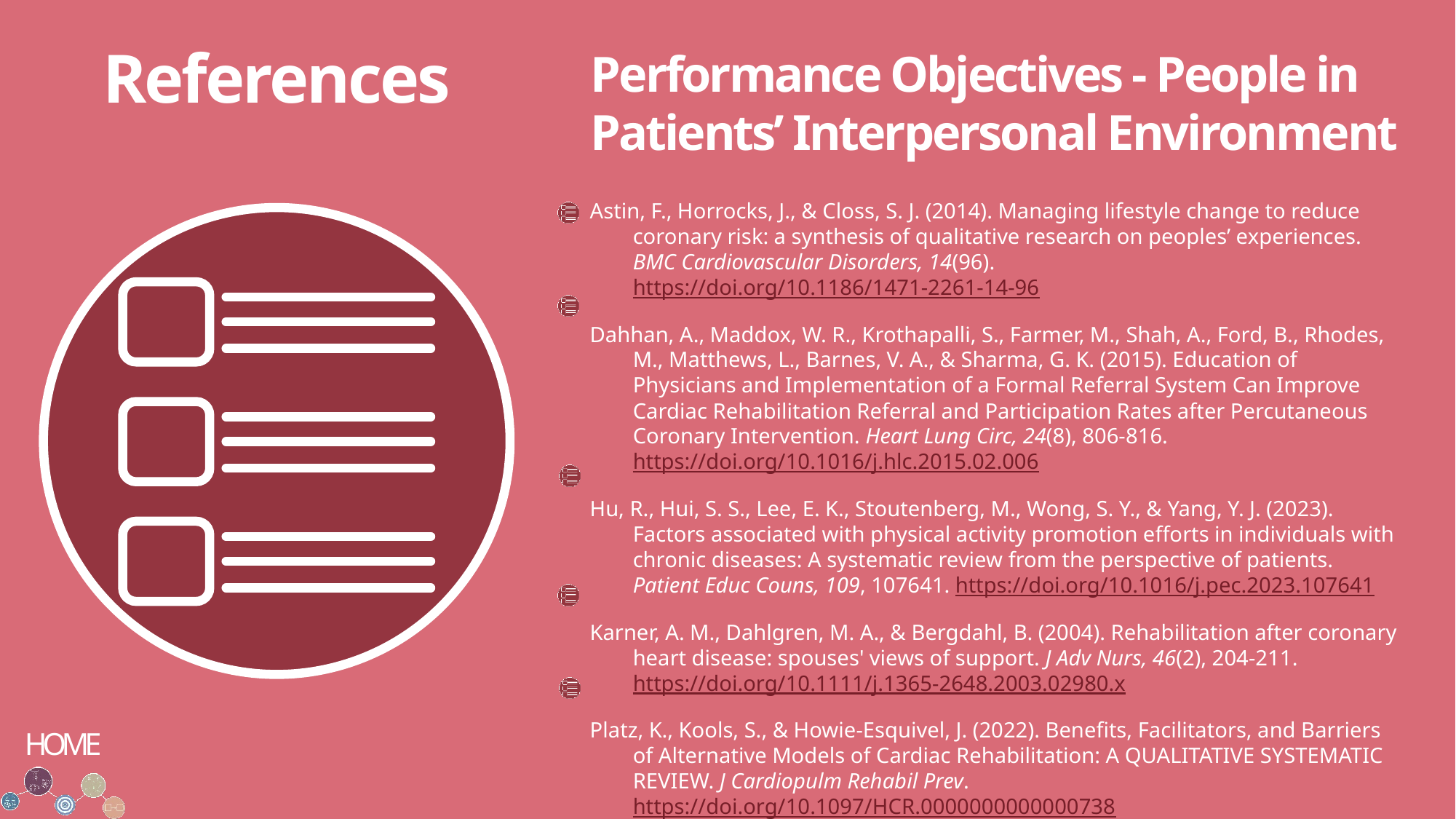

Reference list POs for people in the patient’s interpersonal environment
References
Performance Objectives - People in Patients’ Interpersonal Environment
Astin, F., Horrocks, J., & Closs, S. J. (2014). Managing lifestyle change to reduce coronary risk: a synthesis of qualitative research on peoples’ experiences. BMC Cardiovascular Disorders, 14(96). https://doi.org/10.1186/1471-2261-14-96
Dahhan, A., Maddox, W. R., Krothapalli, S., Farmer, M., Shah, A., Ford, B., Rhodes, M., Matthews, L., Barnes, V. A., & Sharma, G. K. (2015). Education of Physicians and Implementation of a Formal Referral System Can Improve Cardiac Rehabilitation Referral and Participation Rates after Percutaneous Coronary Intervention. Heart Lung Circ, 24(8), 806-816. https://doi.org/10.1016/j.hlc.2015.02.006
Hu, R., Hui, S. S., Lee, E. K., Stoutenberg, M., Wong, S. Y., & Yang, Y. J. (2023). Factors associated with physical activity promotion efforts in individuals with chronic diseases: A systematic review from the perspective of patients. Patient Educ Couns, 109, 107641. https://doi.org/10.1016/j.pec.2023.107641
Karner, A. M., Dahlgren, M. A., & Bergdahl, B. (2004). Rehabilitation after coronary heart disease: spouses' views of support. J Adv Nurs, 46(2), 204-211. https://doi.org/10.1111/j.1365-2648.2003.02980.x
Platz, K., Kools, S., & Howie-Esquivel, J. (2022). Benefits, Facilitators, and Barriers of Alternative Models of Cardiac Rehabilitation: A QUALITATIVE SYSTEMATIC REVIEW. J Cardiopulm Rehabil Prev. https://doi.org/10.1097/HCR.0000000000000738
HOME

## Slide 91
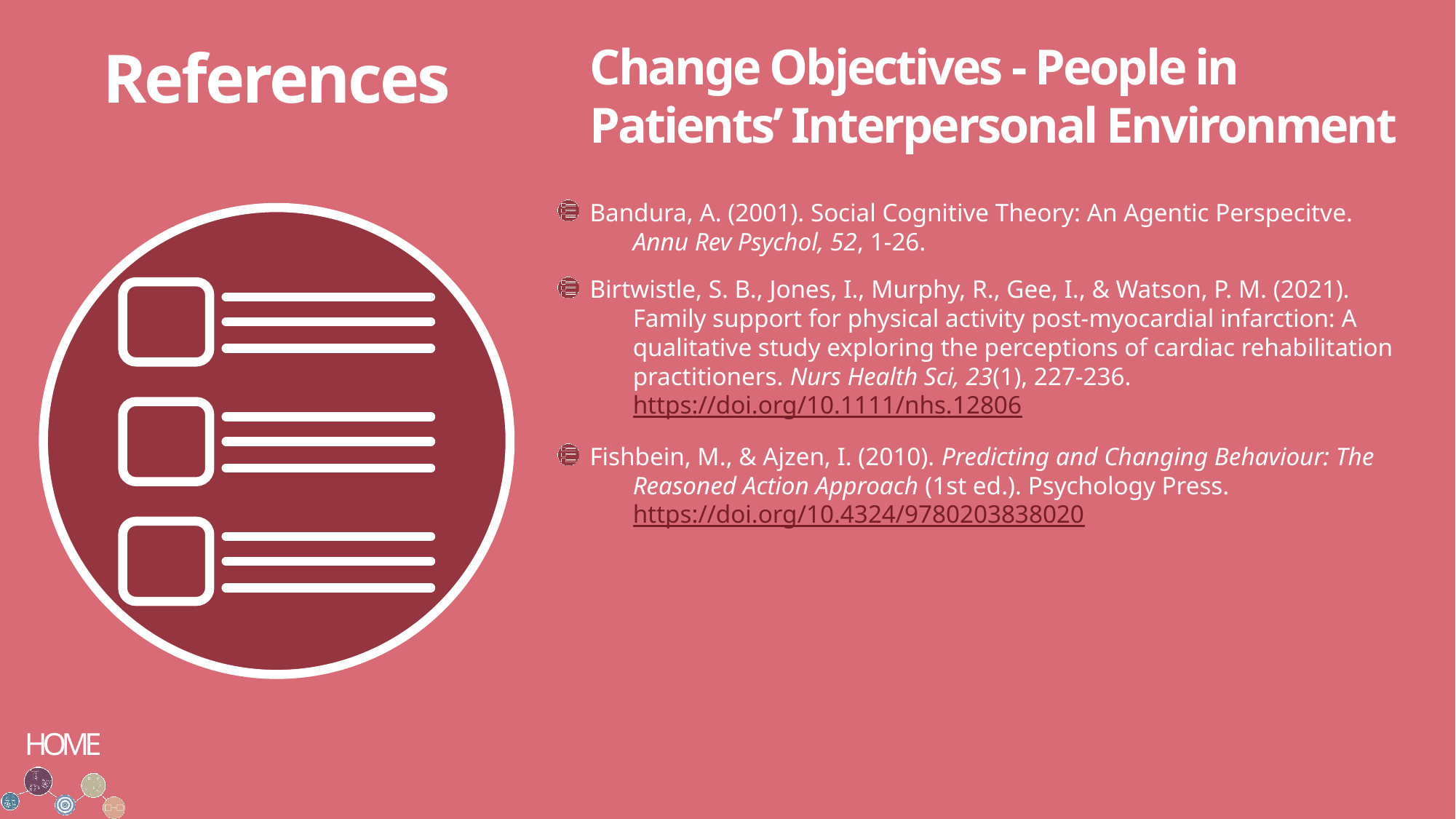

Reference list COs for people in the patient’s interpersonal environment
References
Change Objectives - People in Patients’ Interpersonal Environment
Bandura, A. (2001). Social Cognitive Theory: An Agentic Perspecitve. Annu Rev Psychol, 52, 1-26.
Birtwistle, S. B., Jones, I., Murphy, R., Gee, I., & Watson, P. M. (2021). Family support for physical activity post-myocardial infarction: A qualitative study exploring the perceptions of cardiac rehabilitation practitioners. Nurs Health Sci, 23(1), 227-236. https://doi.org/10.1111/nhs.12806
Fishbein, M., & Ajzen, I. (2010). Predicting and Changing Behaviour: The Reasoned Action Approach (1st ed.). Psychology Press. https://doi.org/10.4324/9780203838020
HOME

## Slide 92
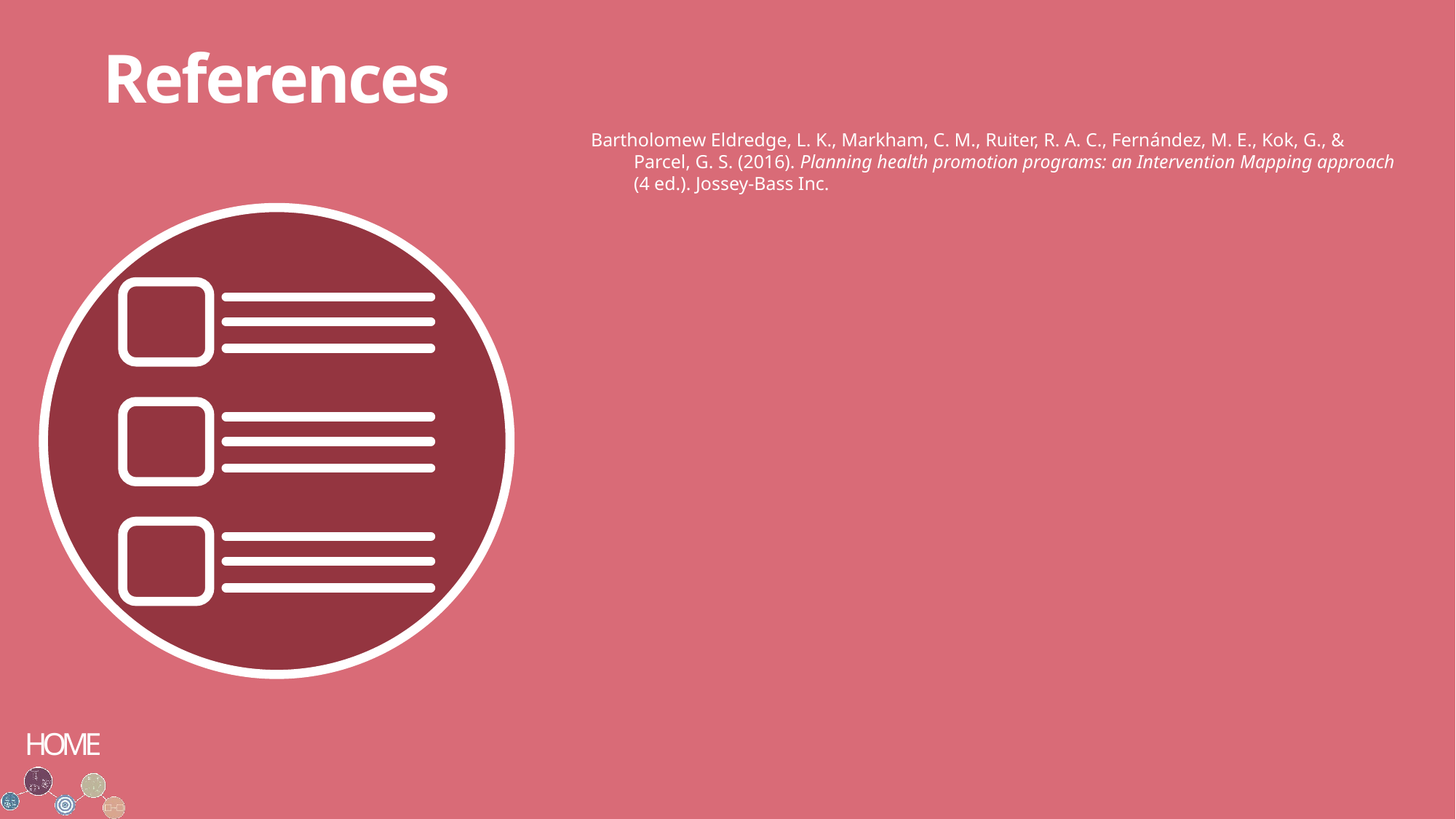

Reference list miscellaneous
References
Bartholomew Eldredge, L. K., Markham, C. M., Ruiter, R. A. C., Fernández, M. E., Kok, G., & Parcel, G. S. (2016). Planning health promotion programs: an Intervention Mapping approach (4 ed.). Jossey-Bass Inc.
HOME
