# Supplementary material for: Physical activity interventions for the cardiac population: A generic logic model based on intervention mapping
Source: PLoS One. 2025 May 14;20(5):e0322807. doi: 10.1371/journal.pone.0322807 (PMC12077670; doi:10.1371/journal.pone.0322807)
Supplement: Attachment — Submitted filename: Response to Reviewers1.docx [file pone.0322807.s004.docx]

**Journal Requirements**

1. *When submitting your revision, we need you to address these additional requirements. Please ensure that your manuscript meets PLOS ONE's style requirements, including those for file naming. The PLOS ONE style templates can be found at*
   [*https://journals.plos.org/plosone/s/file?id=wjVg/PLOSOne_formatting_sample_main_body.pdf*](https://journals.plos.org/plosone/s/file?id=wjVg/PLOSOne_formatting_sample_main_body.pdf) *and* 
   [*https://journals.plos.org/plosone/s/file?id=ba62/PLOSOne_formatting_sample_title_authors_affiliations.pdf*](https://journals.plos.org/plosone/s/file?id=ba62/PLOSOne_formatting_sample_title_authors_affiliations.pdf)*.*

AUTHOR RESPONSE: We have amended the manuscript to fit the PLOS One style requirements. The figures and supplementary files are now named appropriately (e.g. S1 File). We also have clarified the author contributions according to the PLOS ONE guidelines.

2. *We note that you have indicated that there are restrictions to data sharing for this study. PLOS only allows data to be available upon request if there are legal or ethical restrictions on sharing data publicly. For more information on unacceptable data access restrictions, please see* [*http://journals.plos.org/plosone/s/data-availability#loc-unacceptable-data-access-restrictions*](http://journals.plos.org/plosone/s/data-availability#loc-unacceptable-data-access-restrictions)*.

   Before we proceed with your manuscript, please address the following prompts:*
   a. *If there are ethical or legal restrictions on sharing a de-identified data set, please explain them in detail (e.g., data contain potentially identifying or sensitive patient information, data are owned by a third-party organization, etc.) and who has imposed them (e.g., a Research Ethics Committee or Institutional Review Board, etc.). Please also provide contact information for a data access committee, ethics committee, or other institutional body to which data requests may be sent.*

AUTHOR RESPONSE: Thank you for clarifying PLOS' data sharing policy. We apologize for any confusion caused by the earlier indication of data sharing restrictions. We would like to clarify that our study is a theoretical one based on existing published research, we did not collect any data ourselves. Therefore, there are no data associated with this manuscript that require sharing.

**Editor Comments:**

1. *Your manuscript title should include the type of the study.*

AUTHOR RESPONSE: We have altered the title of our manuscript to include the type of study. Our manuscript is now titled: *Physical activity interventions for the cardiac population: a generic logic model based on Intervention Mapping.*

**Reviewers’ comments to the authors:**

**Reviewer #1**

1. *The generic model must be translated into different languages.*

AUTHOR RESPONSE: Thank you for the suggestion. We agree that translating the generic logic model into different languages could significantly enhance accessibility and broaden its reach to researchers and practitioners worldwide. However, we believe a translation of the logic model falls outside of the scope of the current manuscript, especially given the novelty of the concept. Please note, however, that in terms of content, it can be used by intervention developers across the globe.

2. *The authors did not mention the number of cases they applied the model on.*

AUTHOR RESPONSE: Thank you for your suggestion. Within the discussion under the *solidifying existing interventions* section, we have included a reference in which the generic logic model is used. On page 15, lines 279 - 281 we now state:

*“This was demonstrated by Marcos et al. [48], who used the generic logic model developed in this study, to map the underlying rationale behind the aktivplan programme.”*

3. *The model must be of reliability and validity tests.*

AUTHOR RESPONSE: Thank you for the feedback. We would like to clarify that the focus of the current manuscript is the development of a logic model upon which interventions for the maintenance of physical activity can be developed. This manuscript itself does not report on the development of an intervention using the logic model. Furthermore, while the use of a logic model itself promotes the development of reliable and valid interventions, it is important to clarify that the logic model itself is not directly subjected to traditional reliability and validity testing. That being said, when applied in a specific context, the next step would then be to evaluate the intervention in that context.

4. *The references must be in the same form .....doi ..................*

AUTHOR RESPONSE: Thank you for your attention to detail. We have reviewed our references and ensured consistency in their format.

**Reviewer #2**

1. *Please add few examples how logic models have previously been used and successful in sustainability of the behavior.*

AUTHOR RESPONSE: Thank you for the suggestions. Intervention Mapping, and with it logic models, have been shown to develop effective interventions. Within the introduction of our manuscript, we have now included a reference that further elaborates on the effectiveness including practical examples. On pages 5 and 6, lines 98 - 107, we now state the following:

*“**Logic models provide a transparent map of an intervention, explicitly uncovering its underlying assumptions and its proposed or hypothesized mechanisms of action. Methodological guidance for the development and evaluation of behavioural interventions (Template for Intervention Description and Replication (TIDieR) checklist [**10**], United Kingdom Medical Research Council’s framework for developing and evaluating complex interventions [**11**], Workgroup for Intervention Development and Evaluation Research (WIDER) [**12**]) recommends the use of logic models. Therefore, developers and investigators of behaviour change interventions are urged to – and often do – report logic models (e.g., [**13**]). Despite that, these are usually specific to the intervention, they intend to convey a comprehensive understanding of that specific intervention.”*

2. *Need more clarification on how the core processes embedded in IM were used to formulate the POs and COs and uncover the determinants.*

AUTHOR RESPONSE: Thank you for the insightful feedback regarding the need for further clarification on how the core processes were used. We have addressed this by elaborating on the specific contribution of each core process. This explanation is now included on page 10, lines 203 - 215, of the manuscript, and reads as follows:

*“After posing the questions, a brainstorm using free association and the researchers’ expertise yielded an initial list of potential answers. For example, the question “What do people who had an acute cardiac event need to do to increase and maintain their heart-healthy physical activity?” was answered by “Adjusting exercises to their physical capabilities”. The subsequent core process in which existing research and literature was reviewed, validated or disregarded these answers. In the next core process, to provide a more complete answer to the questions, general behaviour theories such as, but not limited to, The Reasoned Action Approach [38], the Health Action Process Approach [39], the I-Change Model [40, 41] and the Social-Ecological model [42] were consulted. Research gaps were identified hereafter, and a final list of answers was established. These processes were then repeated to formulate and uncover the other POs, COs, and determinants.”*

3. *Results need further elaboration with practical examples.*

AUTHOR RESPONSE: Thank you for your feedback. Within our discussion section we have now referred to a recent publication in which the generic logic model is applied to the aktivplan intervention (page 15, lines 279 - 281). Furthermore, the supplementary files S1 File and S2 File include several practical examples. The screenshots below show an excerpt from the interactive S2 File.

Figure 1a depicts one of the POs developed to increase and maintain the physical activity levels of the cardiac population, namely the action of planning weekly physical activity. Below the PO, several relevant determinants are listed. When one clicks on one of the determinants in the supplementary interactive file S2 File, they will be taken to a screen similar to Figure 1b. Figure 1b depicts the CO connected to the self-efficacy determinant for the aforementioned PO. Figure 1b further shows a suggestion on how to target this specific determinant in an intervention and the method used to target the determinant. In the interactive S2 File, such practical examples are provided for all COs and their respective determinants contained in the generic logic model.

Fig 1a	Fig 1b
