# Supplementary material for: Physical activity interventions for the cardiac population: A generic logic model based on intervention mapping
Source: PLoS One. 2025 May 14;20(5):e0322807. doi: 10.1371/journal.pone.0322807 (PMC12077670; doi:10.1371/journal.pone.0322807)
Supplement: Attachment — Submitted filename: Response to reviewers.docx [file pone.0322807.s005.docx]

**Journal Requirements**

1. *Please review your reference list to ensure that it is complete and correct. If you have cited papers that have been retracted, please include the rationale for doing so in the manuscript text, or remove these references and replace them with relevant current references. Any changes to the reference list should be mentioned in the rebuttal letter that accompanies your revised manuscript. If you need to cite a retracted article, indicate the article’s retracted status in the References list and also include a citation and full reference for the retraction notice.*

AUTHOR RESPONSE: Thank you for the reminder to ensure compliance with the journal’s guidelines. We have carefully reviewed the list again to confirm all references are up-to-date. To our knowledge, the reference list is both complete and accurate, and none of the cited papers have been retracted.

**Reviewers’ comments to the authors:**
**Reviewer #3**

1. *This is a very complex piece of work, and (I appreciate) difficult to describe within the remit of a journal article. It explains a process which is rarely described in the literature, and I have appreciated the opportunity to read it. The initial reviewer comments have been addressed. Referring to the revised document, I have some minor comments:*

AUTHOR RESPONSE: Thank you very much for your kind words and constructive feedback. We have addressed all posed comments and made the corresponding revisions to our manuscript. Please find a detailed description of our alterations below.

2. *Line 93 'a range of factors' would be a better phrase than 'various factors' (which makes this sentence feel vague).*

AUTHOR RESPONSE: Thank you for this suggestion. We have replaced the phrase ‘various factors’ with ‘a range of factors’ to enhance clarity. (page 5, line 88)

3. *Line 128 Could you say 'the individual patient' rather than 'the individual' - to be specific (as the patient is another 'agent')?*

AUTHOR RESPONSE: We have revised the term as suggested, adding ‘patient’ to improve specificity for the reader. (page 7, line 123)

4. *Lines 141-5 I had to re-read this paragraph more than once to understand it. You finish the first sentence by specifying a population, and begin the following sentence with a reference to 'their influence' (the patients?). If you put the subject of the following sentence first - 'People in the patient's interpersonal environment...because of their influence on the patient's behaviour and social environment' - this will read grammatically.*

AUTHOR RESPONSE: Thank you for highlighting this issue. We have restructured the sentence as suggested. It now reads:

*“Healthcare providers (e.g. doctors, nurses, sports scientists, physiotherapists) [**15**] and people in the patient's interpersonal environment (e.g. spouse, other family members, friends) [**16**,* *17**] were chosen as two key environmental agents, due to their influence on the behaviour and (social) environment of the patients.”* (page 7, lines 136-141)

5. *My main comment is that I was expecting to see a figure showing the logic model of change (the solution-oriented model you would apply in intervention development) rather than (or as well as) the logic model of the problem. This appears in the Marcos et al paper you've now cited. Table two does outline the constructs associated with change, but it would be very helpful to the reader to have a concise diagrammatic representation of the change model (many readers might not open your supplementary files).*

AUTHOR RESPONSE: We appreciate this comment. To address it, we have added a figure depicting the logic model of change. As there are numerous change objectives, performance objectives, and determinants, we opted to focus on one performance objective per agent in this figure to maintain clarity and provide a concise overview (page 9, line 179; also depicted on the page below)
